# Integrating multiomic resources and gene expression studies to identify candidates for engineering climate-resilient *Brassica*

**DOI:** 10.3389/fpls.2026.1747148

**Published:** 2026-07-07

**Authors:** Rafaqat Ali Gill, Md Mostofa Uddin Helal, Qian Xing, Skhawat Ali, Weijun Zhou, Ralf Müller-Xing

**Affiliations:** 1 Jiangxi Provincial Key Laboratory of Plant Germplasm Innovation and Genetic Improvement, Lushan Botanical Garden, Chinese Academy of Sciences, Jiujiang, China; 2 Plant Epigenetics and Development, Lushan Botanical Garden, Chinese Academy of Sciences, Nanchang, China; 3 College of Life Science, Nanchang University, Nanchang, China; 4 Plant Molecular Biotechnology Laboratory,Department of Biochemistry and Molecular Biology,University of Rajshahi, Rajshahi, Bangladesh; 5 Institute of Crop Science, Ministry of Agriculture and Rural Affairs Key Laboratory of Spectroscopy Sensing, Zhejiang University, Hangzhou, China

**Keywords:** abiotic stress tolerance, *Brassica napus* L., climate-resilient breeding, gene overexpression, QTL and GWAS integration, transcriptomics

## Abstract

Climate-related stresses, including drought, salinity, temperature extremes (cold and heat), and waterlogging, substantially constrain *Brassica napus* productivity, particularly when they occur during reproductive development or as compound stresses. Because *B. napus* is an allotetraploid species, stress-resilience traits are shaped by polygenic inheritance, gene redundancy, and subgenome-specific regulation. This review integrates QTL mapping, GWAS, transcriptomic evidence, and functional studies to prioritize candidate genes and pathway-level modules associated with climate-resilience. Drought and salinity candidates converge on ABA signaling, osmotic adjustment, proline biosynthesis, aquaporin-mediated water transport, and ion-homeostasis pathways, including SOS and NHX-related components. Temperature resilience is associated with *CBF*/*DREB*-mediated cold acclimation and HSF-HSP-DREB2A-linked proteostasis under heat stress. Waterlogging tolerance is linked to hypoxia and ethylene signaling, redox protection, and CIPK15/SnRK1-related energy regulation. We distinguish positional candidates from expression-supported and experimentally validated genes and discuss how these targets can be used in MAS, genomic selection, allele pyramiding, and genome editing. Current evidence supports pathway-level convergence, but causal validation of individual *B. napus* gene copies and evaluation of yield trade-offs remain major priorities.

## 1 Background

*Brassica napus* L. (rapeseed/canola), is a major oilseed crop used for edible oil, animal feed, and biofuel (Borges et al., 2023; Janni et al., 2024; Pandey et al., 2025). Its productivity is increasingly constrained by drought stress (DS), heatwaves (heat stress, HS), chilling (freezing/cold stress, CS), flooding (waterlogging stress, WS), and salinity stress (SS) (Lohani et al., 2020; Borges et al., 2023; Secchi et al., 2023; Mohan et al., 2025). DS and SS mainly impair water balance, osmotic adjustment, and ion homeostasis, whereas HS and CS disrupt reproductive development, membrane stability, and photosynthetic performance (Lohani et al., 2020; Borges et al., 2023; Raman et al., 2023; Secchi et al., 2023; Le Roux et al., 2025). WS imposes root-zone hypoxia and reoxygenation-associated oxidative stress, which disrupt energy metabolism and redox homeostasis (Li et al., 2022; Zhang et al., 2025c). Because stress tolerance in *B*. *napus* is quantitatively inherited and influenced by developmental stage and environment, genetic improvement requires integration of QTL mapping, GWAS, transcriptomics, and functional validation rather than reliance on single-marker associations (Li et al., 2018; Ding et al., 2020a; Raman et al., 2023; Salami et al., 2024a).

*B. napus* is particularly sensitive to fluctuations in temperature and erratic precipitation, both of which negatively affect its physiological processes, growth stages, and reproductive success (Borges et al., 2023; Batool et al., 2025). For instance, temperatures exceeding 27 °C can cause floral abortion and reproductive sterility (Young et al., 2004), with the period from early flowering to pod filling being especially vulnerable to HS (Le Roux et al., 2025). These vulnerabilities make *B. napus* particularly sensitive to ongoing climate shifts, with consequences for yield stability and production (Janni et al., 2024; Pandey et al., 2025). Given its role as a major source of edible oil, protein-rich animal feed, and renewable biofuel, enhancing the climate resilience of *B. napus* is crucial. This can be achieved through targeted breeding, genomic integration, and genome-editing strategies to improve its tolerance to these stresses.

A central component of stress adaptation in *B. napus* is the regulation of reactive oxygen species (ROS) production, signaling, and detoxification. The interplay between ROS, acting simultaneously as damaging agents and critical signaling molecules, and intrinsic antioxidant networks directly dictates the plant’s overall resilience (Kesawat et al., 2023; Mohan et al., 2025; Rao et al., 2025). In response to DS and SS, ROS mainly trigger antioxidant systems to combat osmotic and ionic stress (Dai et al., 2025; Rao et al., 2025). In temperature-related stresses like HS and CS, ROS help modulate reproductive development and photosynthesis, essential processes for maintaining productivity under extreme temperatures (Sharma et al., 2021; Xu et al., 2024; Khan et al., 2025). Waterlogging, which induces hypoxia (a lack of oxygen), exacerbates ROS accumulation, further challenging the plant’s resilience (El-Khateeb et al., 2023; Zhang et al., 2025c). These ROS-mediated pathways work in tandem with other complex signaling networks, such as calcium ions (Ca^2+^) and abscisic acid (ABA), which coordinate the plant’s responses to various stresses (Tong et al., 2021; Dousti et al., 2025; Luo et al., 2025). Collectively, understanding the role of ROS and how they interact with these complex signaling networks is critical for improving stress tolerance in *B. napus*.

Most climate-resilience traits in crops are quantitatively inherited and controlled by multiple loci with environment-dependent effects. The polyploid nature of *B. napus* complicates the genetic dissection of stress tolerance traits, making the integration of Quantitative Trait Loci (QTL) mapping and Genome-Wide Association Studies (GWAS) essential to identify causal genes. Combining QTL mapping with GWAS improves the resolution of stress-associated genomic regions, but causal genes usually require transcriptomic, and functional evidence. In *B. napus*, several key traits have been identified for climate-related stress tolerance through QTL and GWAS studies. For DS tolerance, traits such as water management, osmotic adjustment, yield, antioxidant activity, and phenological traits have been studied (Mickelbart et al., 2015; Raman et al., 2023; Salami et al., 2024a). For SS tolerance, important traits include root and shoot growth, ion homeostasis, chlorophyll content, and germination vigor (Wan et al., 2018; Wu et al., 2019; Wassan et al., 2021; Zhang et al., 2024). CS tolerance is associated with traits like freezing/frost damage score, relative electrolyte leakage (REL), chlorophyll fluorescence (Fv/Fo and Fv/Fm), and frost damage index (FDI) (Horvath et al., 2020; Chao et al., 2021; Qin et al., 2022; Zheng et al., 2025). HS tolerance is linked to pod abortion, pollen sterility, heat susceptibility index (HSI), and plant height (Rahaman et al., 2017; 2018). WS tolerance is assessed through traits such as shoot elongation, aerenchyma formation, root and hypocotyl length, root fresh weight (RFW), and survival/seedling death rate (Mickelbart et al., 2015; Voesenek and Bailey‐Serres, 2015; Ding et al., 2020a; Wang X, et al., 2020b).

For the precise identification of candidate genes related to complex climate-resilience traits, integrating QTL/GWAS-detected genomic regions with gene expression analyses (e.g., transcriptomics, RNA-Seq, and Weighted Gene Co-expression Network Analysis, WGCNA) offers a promising strategy to overcome the limitations inherent in QTL and GWAS alone (Li et al., 2018; Guo et al., 2019). Although QTL and GWAS studies identify broad genomic regions associated with traits, pinpointing the functional genes requires complementary transcriptomic evidence. While QTL and GWAS identify broad genomic regions, integrated multi-omics and functional validation are required to pinpoint causal genes and define functional modules. Consequently, throughout this review, candidate genes are strictly prioritized based on convergence of mapping, expression, and functional evidence.

QTL-detected regions are often large, but when integrated with gene expression data, specifically differentially expressed genes (DEGs) between control and treated plants, this approach helps narrow down the list of candidate genes (Ding et al., 2020a). For example, combining GWAS with transcriptomic studies has successfully identified genes involved in lignin biosynthesis in *B. napus* (Wei et al., 2017b). Additionally, GWAS coupled with WGCNA is particularly valuable, as co-expression analyses uncover complex correlation patterns between genes, offering insights into gene networks rather than focusing solely on individual genes (Li et al., 2018). These approaches have prioritized several promising candidate genes, such as *ESK1/TRICHOME BIREFRINGENCE-LIKE 29* (*ESKIMO1*), *CELLULOSE SYNTHASE 6* (*CESA6*), and *FRAGILE FIBER 8* (*FRA8*), which are involved in lodging resistance in *B. napus* (Li et al., 2018).

This review synthesizes QTL and GWAS findings on climate-resilience (DS, SS, HS, CS, and WS)-related traits in *B. napus* and integrates multi-omics and functional validation evidence to highlight high-confidence candidate genes. By combining insights from QTL/GWAS, multi-omics, and gene-validation studies, functional overexpression experiments in both *B. napus* and the model plant *Arabidopsis thaliana*, this study aims to enhance our understanding of climate-resilience mechanisms in *B. napus*.

## 2 QTL mapping and GWAS studies identify significant stress-responsive loci in *B. napus*

To accurately prioritize climate-stress-related candidate genes, they must be systematically categorized based on the robustness of their supporting evidence. In this review, ‘predicted candidate genes’ refer to genes located within QTL or GWAS intervals without direct functional validation in *B*. *napus*. ‘Expression-supported candidates’ refer to genes that co-localize with stress-associated loci and are differentially expressed under respective stress conditions. ‘Experimentally supported or validated candidates’ refer to genes whose function has been tested through overexpression, gene editing, mutant analysis, allele analysis, or physiological validation. This distinction is crucial because not all genes located inside a stress-associated interval should be treated as causal. The strongest candidates are those where genetic mapping, expression response, pathway function, and phenotype-level evidence converge. A comprehensive list of all QTLs and SNP markers identified across these five stresses, including their genomic positions and target traits, is provided in **Supplementary Table 1**.

### 2.1 Drought stress

#### 2.1.1 QTL/GWAS findings

In field-grown rapeseed, DS tolerance is a complex quantitative trait as it is controlled by many loci of small-to-moderate effect, and its expression is profoundly influenced by the environment, including the timing, intensity, and duration of water deficit. In *B. napus*, both bi-parental QTL mapping and GWAS are therefore often used side-by-side (Khanzada et al., 2020; Gad et al., 2021).

At the early developmental stages, drought phenotyping commonly relies on germination and seedling traits that reflect dehydration avoidance and early vigor. Many studies summarize these traits using DS indices (DSI) or related indices, because they capture performance under stress relative to control conditions and help stabilize comparisons across environments (Khanzada et al., 2020; Gad et al., 2021). In a seed germination/seedling QTL study, 39 QTLs were reported and consolidated into 36 consensus QTLs, with several loci affecting DSI for key traits such as germination percentage (GP), root length (RL), shoot length (SL), root fresh weight (RFW), shoot fresh weight (SFW) and root-to-shoot ratio (R/S) (Zhang et al., 2015; Khanzada et al., 2020; Gad et al., 2021). Chromosome C01 emerged as a major contributor, including stable DSI-QTLs such as qDSI_RL-11–1 and qDSI_SL-11-3 (among several C01 DSI loci), reinforcing C01 as a recurrent genomic region for drought-related seedling performance in *B. napus* (Gad et al., 2021).

Beyond the initial seedling establishment phase, QTL mapping has identified several major effect loci governing plant architecture and drought escape. For instance, a notable QTL for root vigor (NRV) was localized on A01, explaining 16.3% of phenotypic variation (Arifuzzaman et al., 2016). Multi-environment QTL analyses have further delineated stable regions across contrasting water regimes. A major stable QTL on A09 has been identified that accounts for up to 17.81% of variance for multiple traits, including seed yield, flowering time, and carbon isotope discrimination (Δ13*C*). This suggests A09 is a candidate genomic region for further validation (Raman et al., 2023). Additionally, the plasticity of seed yield in response to water deficit is governed by specific QTL by environment (QE) interactions on A02 and C09, which exhibit opposite allelic effects under well-watered versus stressed conditions (Raman et al., 2023). Recent multi-environment analyses have also expanded the genomic landscape to include seed quality traits. A GWAS investigating oil and protein content under drought identified 38 significantly associated SNP loci, including a unique marker on C03 (Bn-scaff_16755_1-p1427195) that regulated both traits simultaneously (Hu et al., 2023).

GWAS at the seedling stage has significantly deepened the genomic landscape of drought tolerance in *B. napus* by utilizing high-density SNP arrays to identify novel marker-trait associations (Khanzada et al., 2020; Lu et al., 2024). Studies employing stress tolerance and susceptibility indices (STI/SSI) identified 314 SNPs across 19 chromosomes, with the highest concentration of associations, including for root fresh weight (RFW) and seedling vigor index (SVI), localized on C02 (Khanzada et al., 2020). This research further characterized ten hotspot or cluster regions (genomic windows carrying multiple significant associations) on A05, A08, C02, C03, C06, and C09, which are valuable for haplotype-based selection as they likely represent conserved regulatory modules for root growth and stress signaling hubs (Khanzada et al., 2020; Batool et al., 2023). Complementing these findings, the use of a composite D value as a comprehensive drought resilience indicator uncovered 37 significant SNP loci, with major association peaks forming on A01, A03, C06, and C08 (Lu et al., 2024).

Beyond seedling vigor, for drought adaptation, balance between drought escape and dehydration avoidance is crucial for maintaining water status through roots and stomata. GWAS has targeted physiological traits such as leaf water loss ratio (WLR) to evaluate dehydration avoidance (Shahzad et al., 2021). This approach identified 139 associated SNPs, the largest number of associations mapped to A10. Among 139, 13 SNPs were significantly associated with WLR, suggesting this region is critical for stomatal regulation and the preservation of leaf water status (Shahzad et al., 2021). Complementing WLR data, GWAS for water holding capacity (WHC) in natural-variation populations identified ten associated signals, with the most significant peak (S9_19159584) localized on A09 (Wang et al., 2024a). These high resolution maps are further supported by comparative findings in related species like *B*. *juncea*, where 77 SNPs (integrated into 27 QTLs) were identified for relative germination rate and seedling fresh weight, including a highly stable QTL on A04 detected across multiple years and environments (Zhang et al., 2025b). Recent association mapping has also identified a novel, major signal for FT on C02, which harbored over 52% of the trait-associated SNP in diverse populations (Salami et al., 2024a). Early flowering can reduce the exposure of reproductive tissues to terminal drought, which helps explain why FT loci often co-localize with DS response loci. A field QTL study under irrigated versus rainfed conditions showed that FT and root-related traits can be genetically correlated and constrained by co-localized QTLs, meaning that improving one strategy can unintentionally shift the other unless loci are carefully managed in breeding (Fletcher et al., 2014). These GWAS discoveries are supported by bi-parental QTL mapping, which identified a significant locus for NRV on A01, as well as distinct FT loci (DTF1 and DTF2) on C08 and C04 (Arifuzzaman et al., 2016). Collectively, these findings provide a high-resolution map of the genetic architecture governing the balance between root capacity, leaf physiology, and phenological adaptation to drought (Fletcher et al., 2014; Raman et al., 2023).

**Figure 1:**
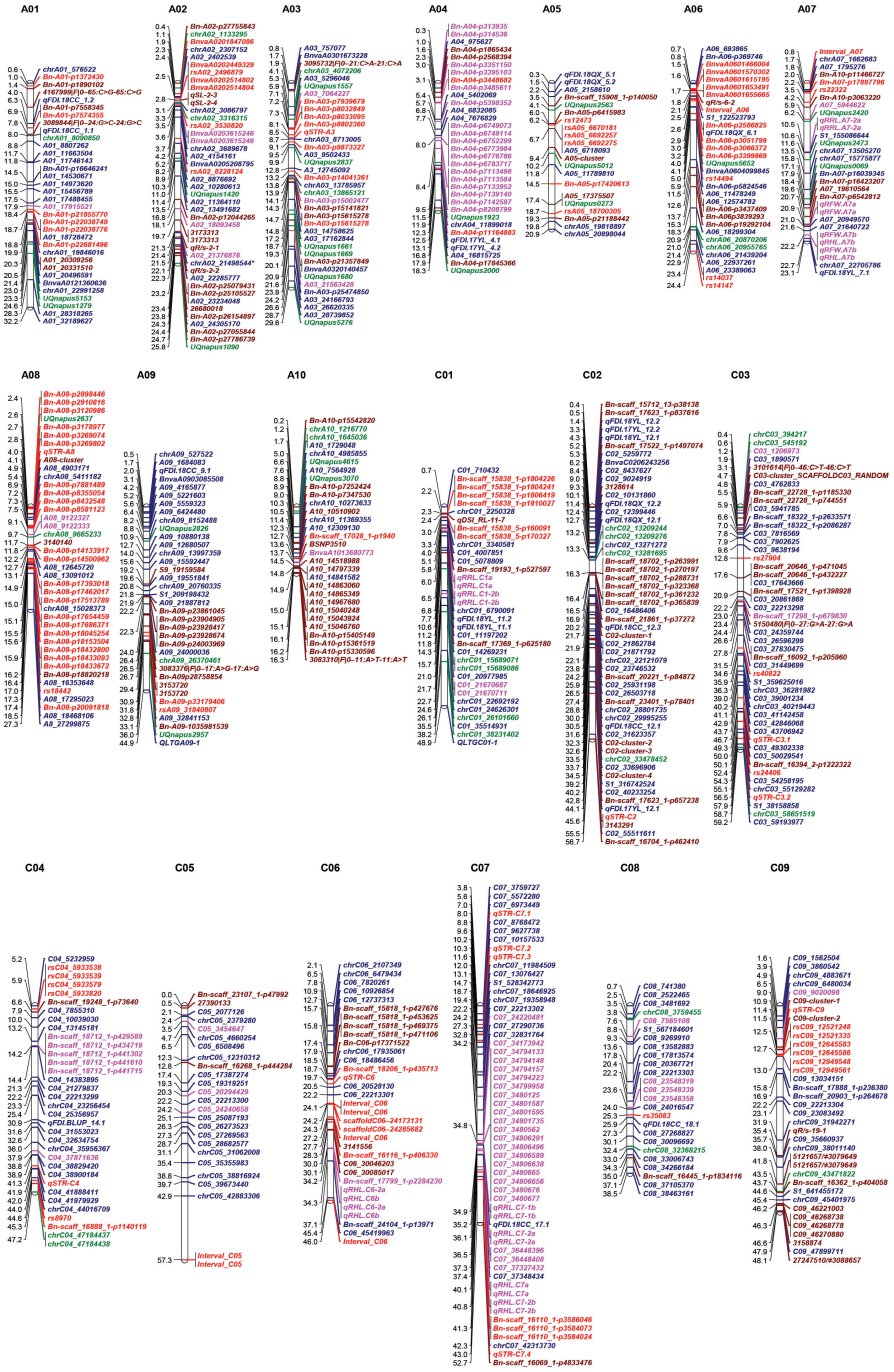
Genetic/genomic landscape of climate-related stresses in *B. napus*. Based on a total of 21 studies using QTL and GWAS techniques, 368 significant QTL/SNP marker positions were identified across 19 chromosomes, targeting drought (96), heat (27), cold/freezing (97), salinity (88) and waterlogging (60). Brown represents drought, green heat/high-temperature, red salt/salinity, navy blue cold/freezing/low-temperature, and pink waterlogging/submergence stress. To visualize all QTL positions across the 19 chromosomes, we used MapChart version 2.3 (Voorrips, 2002; https://doi.org/10.1093/jhered/93.1.77). For detailed information on each QTL/SNP position, including the targeting phenotypic traits and citation, refer to supplementary **Supplementary Table 1**.

Overall, drought tolerance in *B. napus* is genetically anchored by early-stage vigor (C01) and seedling vigor hotspots (C02), while field resilience reflects a critical trade-off between drought escape through earlier flowering and dehydration avoidance (root investment on A10) (Fletcher et al., 2016; Khanzada et al., 2020; Gad et al., 2021). Stable pleiotropic hubs on A09 successfully unify phenology and WUE (e.g., Δ13*C*) across contrasting moisture conditions (Raman et al., 2023). Because drought responses are highly plastic and environment-dependent, validating QTL stability and QE interactions across diverse seasons and soil-moisture gradients is essential to maximize utility of MAS (Raman et al., 2023) (**Figure 1**, colored brown).

#### 2.1.2 Key candidate genes

ABA-centered dehydration response and stress-transcription “switches” (seedling survival): In seedling-stage mapping DSI for germination and vigor, chromosome C01 contains recurrent seedling-stage drought loci, indicating a reproducible genomic region for early dehydration response. Within stable C01 DSI-QTL intervals, genes such as *BnaC01g35030D* (cellular response to ABA) and *BnaC01g30750D* (an ICE1 ortholog involved in ABA signaling) have been identified as key contributors to ABA/ROS-mediated seedling protection (Gad et al., 2021). A major GWAS peak on A01 for composite DSI identified *BnNCED3* (*BnaA01g29390D*), which encodes 9-cis-epoxycarotenoid dioxygenase (Endo et al., 2008; Lu et al., 2024). As a rate-limiting enzyme in ABA biosynthesis, *BnaNCED3* is significantly upregulated in drought-resistant accessions during germination, effectively triggering downstream adaptations to prevent desiccation (Endo et al., 2008). Thus, *BnaA01.NCED3* should be considered an expression-supported and mechanistically strong candidate rather than only a positional candidate. Its value comes from convergence between an A01 drought association peak, its role in ABA biosynthesis, and its stress-induced expression in drought-resistant accessions (Endo et al., 2008; Lu et al., 2024). However, direct functional validation in *B*. *napus* is still required to confirm whether natural allelic variation at this locus consistently improves drought tolerance without reducing growth. Complementing these findings, an ABA regulator-rich interval was reported on C03 (qR/S-13-1), highlighting *AFP4* (*BnaC03g32780D*), *DREB2* (*BnaC03g37030D*), *XERICO* (*BnaC03g44440D*), *RD29B* (*BnaC03g45915D*), and *ABR1* (*BnaC03g49530D*) as coherent candidates within a single block (Gad et al., 2021). Functionally, these “switches”, particularly those from the NAC, MYB, and AP2-EREBP families, are compelling because they coordinate metabolic reprogramming and stomatal behavior (Wang et al., 2019a; Batool et al., 2023; Villiers et al., 2024). By regulating cellular protection and downstream stress-gene activation, these loci directly determine the success of seedling growth and establishment under acute water deficit (Villiers et al., 2024). The C03 ABA regulator block should be presented as a predicted-to-expression-supported candidate cluster rather than as individually validated causal genes. *AFP4*, *DREB2*, *XERICO*, *RD29B*, and *ABR1* form a biologically coherent modules because they connect ABA sensitivity, dehydration-responsive transcription, and ROS-related protection (Shinozaki and Yamaguchi-Shinozaki, 2007; Gad et al., 2021; Kim et al., 2024). Their co-occurrence within the same drought-associated interval strengthens the locus-level evidence, but the causal contribution of each gene remains unresolved. This is a clear research gap for fine mapping, allele mining, and targeted validation.

Higher-order regulators controlling ABA balance and natural variation in drought performance: Natural variation in “top-level” regulators is now providing more direct mechanistic targets for breeding than long candidate gene lists alone (Wang et al., 2024a). One prominent example is *BnaA9.NF-YA7*, localized on chromosome A09 through a GWAS for water holding capacity (WHC) (Wang et al., 2024a). Researchers linked nonsynonymous polymorphism (M631) and a promoter variation (CCAAT-box deletion) directly to DS tolerance differences, tying the effect to the fine-tuning of ABA-related regulation and seedling survival (Wang et al., 2024a). Functionally, *BnaA9.NF-YA7* acts with *BnaABF3/4* in a negative feedback loop to prevent excessive ABA signaling, which effectively balances plant growth and stress responses (Wang et al., 2024a). Additionally, GWAS for leaf WLR identified *Bna.A10.PPD5* on A10, which modulates hydrogen peroxide (H_2_O_2_) accumulation in guard cells to facilitate ABA-mediated stomatal closure (Shahzad et al., 2021). A MATE transporter gene (*BnaC09.MATE*) was identified as a candidate for regulating the WLR through its role in the extrusion of toxic compounds and organic acids during DS (Shahzad et al., 2021). Together, these associations implicate TF-mediated regulation as a major control point in drought adaptation (Villiers et al., 2024; Wang et al., 2024a).

Among the drought candidates, *BnaA9.NF-YA7, BnaA10.PPD5*, and *BnaFLC.A10* represent higher-confidence regulatory nodes because they link natural variation to measurable physiological outcomes (Fletcher et al., 2016; Shahzad et al., 2021; Wang et al., 2024a). *BnaA9.NF-YA7* connects the A09 association signals for water-holding capacity with ABA feedback regulation and seedling survival through interaction with BnaABF3/4 (Wang et al., 2024a). *BnaA10.PPD5* links A10 WLR-associated SNPs with guard-cell H_2_O_2_ accumulation and ABA-mediated stomatal closure (Shahzad et al., 2021). Consequently, these loci emerge as convergent, high-priority targets for breeding, whereas neighboring genes remain predicted candidates pending functional validation (Fletcher et al., 2016; Shahzad et al., 2021; Wang et al., 2024a).

Root vigor, architecture, and early growth maintenance (avoidance through acquisition): In the A01 NRV QTL, the study highlighted genes consistent with growth control and development, including GIP1 (GBF Interacting Protein 1) and SAUR-like family proteins (Arifuzzaman et al., 2016). GIP1 acts as a nuclear co-activator that enhances the binding capability of root-specific TFs to promote later organ growth, while SAUR-like genes facilitate cell elongation in response to auxin (Lee et al., 2014). GWAS at the seedling stage highlighted a significant hotspot on C02 associated with RFW and the SVI, suggesting this region contains conserved regulatory modules for early vigor (Khanzada et al., 2020). Furthermore, integrated GWAS and transcriptomics identified *BnNCED3* on A01 as a positive regulator that significantly upregulated in resistant accessions to maintain seedling establishment under acute desiccation (Lu et al., 2024). In related species like *B. juncea*, the WRKY domain gene *BjuBO35910* was co-detected in the underground tissues at both the germination and seedling stages, reinforcing its role in growth maintenance (Zhang et al., 2025b). Identifying loci that frequently affect root vigor and depth provides “breeding leverage points” to sustain productivity when soil moisture becomes patchy or limited (Mickelbart et al., 2015; Siddiqui et al., 2021; Varshney et al., 2021).

The C02 seedling-vigor hotspot should be interpreted at the locus level rather than as a single-gene target. Because it contains multiple SNP associations for RFW and SVI, suggesting that this region may capture a regulatory block controlling early vigor, root growth, and stress signaling (Khanzada et al., 2020; Batool et al., 2023). Therefore, the C02 region is currently more suitable for MAS and haplotype analysis than for direct genome editing (Li et al., 2018; Guo et al., 2019; Khanzada et al., 2020; Ding et al., 2020a).

Water-saving traits that complement stomatal control: cuticle/wax and hydraulic regulation: Candidates govern biophysical barriers and bulk water transport necessary for maintaining plant hydration (Khanzada et al., 2020; Shahzad et al., 2021). Multi-layer GWAS identified *ABCG16*, a member of the ABC transporter G family, as a novel candidate on C04 for plant weight and seed yield plasticity under terminal drought conditions (Salami et al., 2024a). This gene is involved in the movement of wax precursors, which directly modulate cuticle permeability and leaf surface protection (Yadav et al., 2014; Salami et al., 2024a). Similarly, the genomic region on C03 associated with R/S under DS harbors *BnaC03g12050D*, an ortholog for the lipid transfer protein LTP3 (Zhang et al., 2015). LTP3 is significantly upregulated during dehydration and is essential for maintaining cuticle integrity and membrane stability (Zhang et al., 2015).

Hydraulic regulation is further anchored by aquaporin genes localized within significant marker-trait association intervals for seedling vigor and shoot length (Zhang et al., 2015; Khanzada et al., 2020). Orthologs of *BnPIP1* and *BnPIP2* (such as *BnaC06g14590D* and *BnaA03g27130D*) have been mapped to association peaks on chromosomes A03, A05, C01, C02, and C06 (Zhang et al., 2015; Khanzada et al., 2020). These genes regulate membrane water permeability and root axial hydraulic conductivity, allowing tolerant accessions to optimize water extraction and transport to expanding tissues (Khanzada et al., 2020; Tania et al., 2025). Complementing these transporters, *BnaC03g12400D* (an ortholog of Phospholipase C1) was identified within a C03 DSI-QTL, where it likely mediates the PLC-PA signaling pathway required for secondary structural adaptations to osmotic stress (Zhang et al., 2015; Chater et al., 2019; Gad et al., 2021).

This group of candidates provides a clear link between drought-associated loci and water-saving phenotypes because they govern biophysical barriers and bulk water transport required for maintaining plant hydration (Khanzada et al., 2020; Shahzad et al., 2021). *ABCG16* and *LTP3* represents structural water-conservation genes because *ABCG16* regulates wax-precursor transport and cuticle-related protection, while *LTP3* contributes to cuticle integrity and membrane stability under dehydration (Yadav et al., 2014; Zhang et al., 2015; Salami et al., 2024a). PIP1/PIP2 aquaporins represents hydraulic regulators because they regulate membrane water permeability, root axial hydraulic conductivity, and cellular water movement under drought conditions (Zhang et al., 2015; Khanzada et al., 2020; Tania et al., 2025). PLC1 represents a signaling node that may connect membrane lipid signaling with osmotic adjustment through PLC-PA signaling pathway (Zhang et al., 2015; Chater et al., 2019; Gad et al., 2021). Therefore, these candidates should be discussed as a coordinated water-conservation module rather than as independent genes.

Escape-avoidance balance: flowering-time control linked to drought performance: The genomic landscape of *B. napus* is characterized by major pleiotropic hubs that genetically link drought escape through earlier flowering with dehydration avoidance (root mass/root pulling force) (Fletcher et al., 2014, 2016). The *Bna.FLC.A10* pleiotropic hub acts as the primary genetic determinant for the trade-off between drought escape (flowering time) and dehydration avoidance (increased root mass). Natural variation at this locus, specifically a retro-element insertion, dictates whether the plant prioritizes a rapid life cycle to bypass terminal drought or invest in root architecture for water acquisition (Hou et al., 2012; Fletcher et al., 2016; Yin et al., 2020). In addition, GWAS have uncovered high-resolution signals for flowering time on C02 (Salami et al., 2024a). This region harbors *NCED9*, which links ABA biosynthesis to reproductive timing, and GDSL esterase/lipase genes that coordinate lipid metabolism during floral development under DS (Salami et al., 2024a). Furthermore, identification of MADS-box genes like *AGL15*, *AGL16*, and *AGL19* within significant drought-related QTLs on A03 and C03 provides evidence for a shared genetic network governing both floral transition and maturity in response to water deficit (Salami et al., 2024a). These findings highlight the specific alleles required to optimize the trade-off between escaping terminal drought and sustaining vegetative growth (Fletcher et al., 2014, 2016). Recently, GWAS findings utilizing a composite D value as drought resilient indicator have further identified *PHYB* (Phytochrome b) on A05 and *PSBY* (photosystem II core complex protein) on A06 are critical candidates (Lu et al., 2024). These genes play a dual role, as they may assist in accelerating the life cycle to achieve drought avoidance while simultaneously maintaining photosynthetic efficiency and alleviating oxidative damage to the photosynthetic machinery during water deficit (Lu et al., 2024). This underscore how light-signaling and photosystem-related proteins are integrated into the plant’s phenological strategy to ensure reproductive success under DS (Lu et al., 2024).

The above developmental module also explains why drought tolerance cannot be selected only by survival traits. Early flowering may help plants escape terminal drought, but it can reduce vegetative growth and limit yield potential if drought does not occur. Conversely, greater root investment may improve dehydration avoidance but can delay reproduction or reduce biomass allocation to seeds (Fletcher et al., 2014, 2016). Therefore, BnaFLC.A10, C02 FT-associated loci, *NCED9*, *PHYB*, *PSBY*, and MADS-box genes should be framed as trade-off genes. BnaFLC.A10 links flowering time with root investment, while C02 FT-associated loci, *NCED9*, and MADS-box genes connect reproductive timing with drought response. *PHYB* and *PSBY* further link light signaling and photosynthetic protection with drought-resilience phenotypes (Fletcher et al., 2014, 2016; Lu et al., 2024; Salami et al., 2024a). Their value depends on the target environment, drought timing, and breeding objective.

Metabolic and structural reprogramming: osmotic and quality resilience: Candidates in this category govern the synthesis of osmoprotectants and secondary metabolites that stabilize the plant’s internal environment and protect cellular integrity. Specifically on chromosomes A03, A05, C03, and C04, *BnP5CS1* (homolog of AtP5CS1) acts as the primary metabolic switch for osmotic adjustment. It encodes Δ^1^-pyrroline-5-carboxylate synthase, which catalyzes the rate-limiting step of proline biosynthesis. Under DS, its marked upregulation triggers an approximately 150-fold increase in proline accumulation in leaves, which is essential for ROS scavenging, preserving membrane integrity, and providing cellular turgor (Wang et al., 2019a). Furthermore the integration of GWAS and seed transcriptomics identified *ABCA9* transporter on A03 as a key candidate for managing changes in seed oil and protein contents under DS (Hu et al., 2023). *ABCA9* likely promotes drought resilience by regulating lipid transport and energy metabolism during the critical seed filling stage, thereby sustaining the accumulation of storage reserves even when carbon assimilation is limited by water scarcity (Kim et al., 2013). These metabolic regulators are essential for maintaining the nutritional quality and yield of industrial rapeseed varieties in the arid environment (Batool et al., 2023).

The seed-quality detected under drought add a breeding dimension beyond seedling survival and vegetative water status. A drought-linked C03 marker was associated with coordinated changes in oil and protein content, while the ABCA9 transporter candidate on A03 was identified through GWAS and seed transcriptomics integration for oil-content response under DS (Hu et al., 2023, 2023). ABCA9 is biologically plausible because the Arabidopsis ABCA9 transporter supplies fatty acids for lipid synthesis in the endoplasmic reticulum, supporting its role as a seed-quality resilience candidate rather than a confirmed drought-tolerance gene (Kim et al., 2013). Therefore, ABCA9 and related seed-quality loci should be described as expression-supported or marker-supported candidates until direct functional validation in *B*. *napus* confirms their effect on seed reserve accumulation under reproductive-stage drought.

Taken together, the drought candidate set can be prioritized into three evidence classes. Class I includes convergence candidates with mapping, expression, mechanistic, or functional support, such as *BnaA01.NCED3*, *Bna9.NF-YA7*, *BnaA10.PPD5*, *BnaFLC.A10*, *BnP5CS1*, and selected PIP aquaporins (Endo et al., 2008; Fletcher et al., 2016; Wang et al., 2019a; Khanzada et al., 2020; Shahzad et al., 2021; Lu et al., 2024; Wang et al., 2024a). These candidates connect mapping evidence with ABA regulation, stomatal control, phenology, osmotic adjustment, or hydraulic regulation. Class II includes locus-supported modules with strong biological plausibility but unresolved causality, such as the C03 ABA-regulatory block, C02 RFW/SVI hotspot, *ABCG16*/*LTP3* cuticle-related candidates, and *ABCA9*/seed-quality candidates (Zhang et al., 2015; Khanzada et al., 2020; Gad et al., 2021; Hu et al., 2023; Salami et al., 2024a). Class III includes interval-based predicted genes that useful for hypothesis generation but should not be presented as validated targets. This evidence-based classification supports marker-assisted selection, haplotype breeding, and targeted genome editing.

#### 2.1.3 Pathway-level insights

The genomic architecture of DS resilience in *B. napus* is highly complex, governed by integrated multigenic modules rather than isolated mechanisms. Instead, QTL/GWAS signals repeatedly converge on integrated modules that coordinate water acquisition (roots), conservation (stomata/cuticle), and cellular protection (Gad et al., 2021; Lu et al., 2024). A clearer drought framework therefore links QTL/GWAS loci to candidate genomic regions, candidate genes to functional modules, functional modules to adaptive phenotypes, and adaptive phenotypes to breeding value. In this model, C01 and C02 loci mainly contribute to early vigor and establishment because C01 carries stable DSI-QTLs such as qDSI_RL-11–1 and qDSI_SL-11-3, while C02 contains association for root RFW and SVI (Khanzada et al., 2020; Gad et al., 2021). A01 and A09 loci regulate ABA-mediated dehydration response through *BnNCED3* and *BnaA.NF-YA7*, respectively (Lu et al., 2024; Wang et al., 2024a). A10 links stomatal regulation and flowering-time trade-offs through *BnaA10.PPD5* and *BnaFLC.A10* (Fletcher et al., 2016; Shahzad et al., 2021). Finally, C03, C04, and A05 loci contribute to osmotic, lipid, and structural protection through candidates such as *P5CS1*, *LTP3*, *ABCG16*, PIP aquaporins, and *PLC1* (Zhang et al., 2015; Wang et al., 2019a; Khanzada et al., 2020; Salami et al., 2024a).

Integrated modules of drought resilience: The genetic architecture of DS tolerance in rapeseed is inherently multigenic, anchored by integrated modules that unify root vigor, stomatal control, and osmotic adjustment (Wang et al., 2019a; Lu et al., 2024). GWAS identifies “hotspot” regions, such as those on C01 and C02, where multiple associations for seedling vigor, germination percentage, and root length overlap (Gad et al., 2021; Lu et al., 2024). These hubs suggest that the plant coordinates early-stage establishment through shared regulatory networks rather than independent pathways (Gad et al., 2021; Lu et al., 2024). For instance, a major peak on C02 accounts for over 52% of associated SNPs for reproductive timing and vigor, effectively linking developmental process with stress resilience (Salami et al., 2024a). These modules interact rather than act independently. ABA biosynthesis through *NCED3* contributes to dehydration response by increasing ABA availability, which supports downstream stomatal regulation and stress adaptation. However, excessive ABA signaling can restrict growth, and *BnaA9.NF-YA7* acts with *BnaABF3*/*4* in a feedback loop that prevents uncontrolled ABA response and balance growth with stress tolerance (Wang et al., 2024a). *BnaA10.PPD5* links ABA signaling with ROS-mediated stomatal movement by modulating guard-cell H_2_O_2_ accumulation and ABA-mediated stomatal closure (Shahzad et al., 2021). PIP aquaporins then determine whether reduced water loss is matched by effective water uptake and transport through their roles in membrane water permeability, root hydraulic conductivity, and whole-plant water status (Yu et al., 2005; Zhang et al., 2015; Wang et al., 2019b; Khanzada et al., 2020). Thus, the drought phenotype depends on the coordination of ABA regulation, ROS signaling, hydraulic control, and osmotic adjustment, rather than on the strength of any single pathway (Wang et al., 2019a; Shahzad et al., 2021; Lu et al., 2024; Wang et al., 2024a).

The ecophysiological trade-off: escape vs avoidance: Field genetics makes the “escape vs. avoidance” paradigm concrete through stable pleiotropic hubs that couple flowering time and root investment (Fletcher et al., 2016; Raman et al., 2023). A critical QTL on A10 co-locates flowering time and root pulling force (root mass) (Mickelbart et al., 2015; Fletcher et al., 2016). On A09, a stable hub repeatedly regulated flowering time, seed yield, and Δ13*C* across diverse moisture conditions. This linkage suggests that improving WUE through Δ13*C* selection may unintentionally shift reproductive phenology unless breeders deliberately separate or pyramid complementary alleles (Raman et al., 2023). This trade-off has direct breeding consequences. In terminal drought regions, early-flowering alleles may be useful because they reduce exposure of reproductive tissues to later-season water deficit, but flowering time and root-related traits can be genetically correlated and constrained by co-localized QTLs (Fletcher et al., 2014, 2016). In environments where drought occurs early or intermittently, the same alleles may be undesirable if they reduce root development or yield potential. Because *Bna.FLC.A10* links reproductive transition with root mass and the drought escape versus dehydration-avoidance balance (Fletcher et al., 2016). Therefore, flowering-time loci such as *Bna.FLC.A10* and C02 flowering time-associated loci should be selected with root traits, WUE, and seed-yield plasticity rather than used as single-marker targets (Fletcher et al., 2014, 2016; Raman et al., 2023; Salami et al., 2024a).

ABA-centered signaling and redox protection: At the signaling level, drought associated regions often implicate ABA-centered networks, explaining the overlap between drought loci and genes for oxidative stress management (Lu et al., 2024; Wang et al., 2024a). Key genes such as *Bna.A01.NCED3*, are significantly upregulated in tolerant genotypes to trigger early adaptive defenses (Lu et al., 2024). For regulatory balance, natural variation in *Bna.A09.NF-YA7* provides a mechanistic route from sequence variation to altered ABA regulation (Wang et al., 2024a). It acts with *Bna.ABF3/4* in a negative feedback loop to prevent excessive ABA signaling layer links environmental perception to metabolic reprogramming, such as the induction of proline synthesis via *BnP5CS1/2* (Wang et al., 2019a, 2024). The ABA-ROS module should also be connected to osmolyte metabolism. ABA-responsive transcription can activate P5CS-mediated proline biosynthesis, while ROS signaling can both damage membranes and acts as a secondary signal for stress-gene activation (Dalal et al., 2009; Wang et al., 2019a; Sachdev et al., 2021; D’oria et al., 2022; Shahsavari et al., 2025). Proline accumulation, LEA proteins, lipid-transfer proteins, and aquaporins function downstream as cellular-protection and water-balance outcomes (Dalal et al., 2009; Zhang et al., 2015; Khanzada et al., 2020; Shahsavari et al., 2025). This gives a direct loci → pathway → phenotype chain. For instance, *NCED3*, *NF-YA7*, and *PPD5* regulate ABA and ROS-linked signaling (Shahzad et al., 2021; Lu et al., 2024; Wang et al., 2024a). P5CS, LEA, LTP, and PIP genes execute osmotic, structural, and hydraulic adaptation (Dalal et al., 2009; Zhang et al., 2015; Khanzada et al., 2020; Shahsavari et al., 2025). The phenotype appears as improved seedling vigor, reduced water loss, better WUE, or yield stability (Khanzada et al., 2020; Shahzad et al., 2021; Raman et al., 2023).

Structural and hydraulic “output layers”: Cuticle/wax and hydraulic pathways represent the practical ‘output layers’ of the drought response, providing traits that are both measurable and selectable traits for breeding (Guadarrama‐Escobar et al., 2024). For water conservation, *Bna.C04.ABCG16* is crucial for managing the transport of wax precursors to reduce non-stomatal water loss (Salami et al., 2024a). For hydraulic regulation, *Bna.C06.PIP1* and *Bna.A03.PIP2* detected at C06 and A03, respectively (Khanzada et al., 2020), tune water flow through membranes to maintain tissue turgor during acute dehydration (Chater et al., 2019; Guadarrama‐Escobar et al., 2024). These ‘output layers’ are also where trade-offs become visible. Increased wax or cuticle-related protection may reduce non-stomatal water loss, but these traits should be evaluated together with gas exchange, leaf expansion, and yield because cuticle modification can affect whole-plant water balance and growth (Yadav et al., 2014; Salami et al., 2024a). Altered aquaporin activity may improve water uptake and hydraulic conductivity under drought, but its benefit depends on coordination with stomatal conductance and whole-plant water status (Yu et al., 2005; Wang et al., 2019b; Khanzada et al., 2020; Tania et al., 2025). Therefore, structural and hydraulic genes should be evaluated together with stomatal conductance, photosynthesis, biomass, and seed yield.

Subgenome dynamics in a polyploid context: Because, *B napus* is an allopolyploid, pathway interpretation must remain “subgenome-aware”. Homologs from A and C subgenomes can diverge in expression and functional contribution (Bird et al., 2021; Ziegler et al., 2025). In the case of *NF-YA7*, significant natural variation associated with drought performance is localized on A09 (*Bna.A09.NF-YA7*), while other homologs on C05 (*Bna.C05.NF-YA7*) may remain monomorphic or contribute differently to signaling balance (Wang et al., 2024a). In the regulation of leaf wax, the ortholog *BnCER1–2* present on A subgenome is functional and induced upon DS. In contrast, its C subgenome counterpart *BnCER1-1*, has been identified as a pseudogene, demonstrating how one subgenome may lose a specific stress-response function over evolutionary time (Wang et al., 2020b). Similarly, genes for proline biosynthesis, such as *BnP5CS1/2* are identified on multiple genomic regions on both subgenomes, including A03, A05, C03, and C04 (Zhang et al., 2015; Wang et al., 2019a). This distribution allows for high levels of proline accumulation but complicates the identification of a single “master” locus. Lastly, the regulatory mechanism of guard cells exhibits correlated responses to ABA, specific members like *BnaC02g37590D* (*MYB30*) and *BnaC04g28450D* (*HSFA7A*) have evolved unique regulatory patterns in *B. napus* that differ from their Arabidopsis orthologs (Villiers et al., 2024). This subgenomic divergence and the resulting genetic redundancy explain why drought loci are frequently reproducible at the pathway or regional level (as “hotspots”) while pinpointing a single causal gene remains a significant challenge for molecular breeding.

Synthesizing these findings, drought adaptation in rapeseed operates as an integrated system governed by phenology, root acquisition, stomatal regulation, osmotic adjustment, ROS buffering, and seed-quality protection (Fletcher et al., 2014, 2016; Khanzada et al., 2020; Shahzad et al., 2021; Raman et al., 2023; Hu et al., 2023; Lu et al., 2024; Wang et al., 2024a). The potential breeding targets are not simply the most frequently detected genes, but the candidates where QTL/GWAS evidence, expression response, physiological function, and yield-related phenotypes converge (Li et al., 2018; Guo et al., 2019; Ding et al., 2020a). These data support *BnaA01.NCED3, BnaA9.NF-YA7, BnaA10.PPD5, Bna.FLC.A10, BnP5CS1*, and selected PIP aquaporins as priority candidates (Fletcher et al., 2016; Wang et al., 2019a; Khanzada et al., 2020; Shahzad et al., 2021; Lu et al., 2024; Wang et al., 2024a). At the same time, unresolved C02, C03, and C09 hotspots represent important research gaps for fine mapping, haplotype analysis, and validation across drought timing, soil type, and developmental stage (Khanzada et al., 2020; Gad et al., 2021; Raman et al., 2023).

### 2.2 Salt/salinity stress

#### 2.2.1 QTL/GWAS findings

Similar to DS, SS in *B. napus* is a quantitatively inherited trait regulated by multiple loci and strong environment effects. While salt tolerance primarily refers to plant responses to neutral salts such as NaCl, which impose osmotic stress and Na^+^/Cl^-^ toxicity that disrupt ion homeostasis and osmotic balance. Salt-alkali tolerance adds alkaline conditions that raise rhizosphere pH, impair nutrient availability, and exacerbate ionic toxicity. Although these traits are often genetically linked, they are regulated by partially distinct mechanisms, necessitating their separate yet integrated genetic analysis in *B. napus* (Wan et al., 2017). Thus, salt-related loci should be interpreted according to stress type, developmental stage, and target trait. Seedling-stage loci mainly capture germination, root elongation, and early biomass maintenance (Wan et al., 2017, 2018; Wassan et al., 2021; Zhang et al., 2022b). Whereas salt-alkali and field based-loci may reflect broader effects on nutrient balance, shoot growth (Zhang et al., 2022a, 2022, 2022). This distinction is crucial because a locus associated with early salt tolerance may not necessarily improve mature-plant performance under saline field conditions (Zhang et al., 2022b).

Because early stages (germination and seedling) are highly sensitive, many studies phenotype seedling establishment and summarize performance using stress indices, such as stress tolerance index (STI) or stress susceptibility index (SSI), that compare trait values under stress relative to control, which improves genotype ranking across environments and populations (Yong et al., 2015; Wan et al., 2017; Wassan et al., 2021). Bi-parental linkage mapping has identified several reproducible loci, larger-effect regions, especially when salt tolerance is scored using integrative physiological traits (Wassan et al., 2021; Zhang et al., 2024). A salt-tolerance QTL study reported up to 45 QTLs across 10 morphological and physiological indicators. This study highlighted a major locus, qSPAD5 region (SPAD is a proxy for leaf chlorophyll content), identified on chromosome A07 (also labeled LG5) across three replicates, which could explain a substantial fraction of variation, reported at approximately 37-51% depending on the replicate (Lang et al., 2017). For salt-alkali tolerance, reduced-representation approaches such as SLAF-Seq (Specific-Locus Amplified Fragment Sequencing) have expanded locus discovery (Zhang et al., 2022a). Mapping studies reported significant SNPs for salt-alkali tolerance and yield related traits, with A10 (and localized regions on chromosomes A03 and A07) emerging as a recurrent hotspot across traits and developmental stages, consistent with the idea that some genomic neighborhoods carry multi-trait, stress-relevant signals that can be captured across tests (Zhang et al., 2022a). More recently, QTL-seq/BSA-seq based studies using bulked extremes and sequencing have been used to accelerate detection of 12 major loci for SS resistance on chromosomes A03, A08, C02, C03, C04, C06, C07, and C09 (Zhang et al., 2024). Although SS tolerance exhibits a complex polygenic architecture, QTL analyses consistently identify a core set of stable consensus regions (e.g., qSPAD5-type signals and A10-centered hotspots) that are particularly valuable for marker development (Lang et al., 2017; Zhang et al., 2023b). To address the limitations of traditional scoring, high throughput phenotyping platforms have quantified over 2,100 image-based traits (i-traits) across multiple salt conditions. As a result, 928 high-quality traits associated with dynamic stress responses were identified in both natural populations and intervariatal substitution line populations (Zhang et al., 2023a). These platforms enable high resolution mapping of salt-alkali tolerance in the field, identifying extreme accessions like ‘X182’ and ‘X252’ and candidate regulators such as *BnABA4* and *BnBBX14* (Zhang et al., 2022a). These results also show that image-based phenotyping can capture salt responses that single end-point traits may miss. However, these i-traits should be treated as phenotyping-derived indicators until they are linked with stable loci, candidate gene expression, and field validation.

Notably, QTLs regulating shoot fresh weight under salinity (STI-SFW), which serves as a key indicator of early seedling vigor, included specific loci such as *Bn-A01-p21855770*, *Bn-A08-p20091818*, and *Bn-scaff_16116_1-p406330*. These loci are essential for maintaining above ground biomass, a trait often severely inhibited by the osmotic and ionic imbalances caused by SS (Wan et al., 2017). In addition, multiple loci controlling germination-related traits, including germination rate, radicle length, and seedling fresh weight, have been consistently reported, highlighting the importance of early-stage biomass and root development in establishing salinity tolerance. These early development traits are critical because seed germination is one of the most sensitive periods to SS, directly impacting final crop density and yield (Zhang et al., 2022b). Furthermore, high-resolution GWAS targeting relative germination rate under stringent salt concentrations (250 mM NaCl) identified 4,749 significant SNPs, with major clusters on chromosomes A03, A06, A07, and A09 (Yang et al., 2024). This study successfully validated two C06-based SNP markers (sacffoldC06–24173131 and scaffoldC06-24285682) that explained up to 9.2% of phenotypic variation and showed 100% consistency with salt-tolerant PGR phenotypes (Yang et al., 2024). Overall, 19 QTLs mapped to subgenome A and only six to subgenome C, suggesting a predominant contribution of subgenome A to early-stage salinity tolerance (Wan et al., 2017). This genetic bias is supported by epigenomic maps showing that the An subgenome typically possesses higher level of epigenetic marks (such as H3K4me3 and H3K27ac) and more active transcription, whereas Cn subgenome exhibits higher level of repressive marks like H3K9me2 and DNA methylation (Zhang et al., 2021). This asymmetry may reflect preferential retention of stress-adaptive regulatory genes or stronger selection pressure on subgenome A during domestication and improvement of rapeseed (Zhang et al., 2021; Kong et al., 2025).

GWAS has consistently reinforced the polygenic architecture of seedling-stage salt tolerance in *B. napus*, revealing that resistance is controlled by numerous small-to-moderate effect loci rather than a few major genes. In a seedling-stage GWAS using 368 accessions, 75 significant SNPs across 14 chromosomes were detected and consolidated into 25 QTLs, explaining 4.21-9.23% of phenotypic variation (Wan et al., 2017). Another comprehensive GWAS utilizing a panel of 228 accessions identified 142 significant SNPs distributed across all 19 chromosomes, with 78 SNPs in the C genome and 64 in the A genome, further supporting a highly polygenic architecture or “many-loci” model for salt tolerance. These markers explained an average of 13.1% of the phenotypic variation (Wassan et al., 2021). Taken together, GWAS expands the locus landscape by identifying many small-moderate effect associations that are essential for developing marker sets and haplotypes for genomic selection (Wan et al., 2018; Wassan et al., 2021). While individual associations may be environment-specific, the most promising breeding targets are those demonstrating cross-study support or biological enrichment, such as the SNPs on chromosome A10 which have been repeatedly linked to both seedling vigor and mature plant traits like height and yield under stress (Wan et al., 2017; Zhang et al., 2022c). These field discoveries are complemented by high-throughput QTL-Seq of extreme bulks, which identified 12 major QTLs for salt tolerance rating (STR) and successfully validated the marker SNP820 for the selection of salt-resistant genotypes (Zhang et al., 2024). Additionally, association mapping has revealed seven candidate genes that pleiotropically control both salt tolerance and yield components (e.g., silique length and dry weight per plant), offering a genetic route to alleviate yield penalties in saline environments (Zhang et al., 2022c). This distinction is important for breeding because salt tolerance can arise from different mechanisms. Some loci support early vigor under osmotic stress, including germination, radicle length, seedling fresh weight, and shoot fresh weight loci (Wan et al., 2017; Zhang et al., 2022b). Some loci regulate salt-alkali adaptation and yield-related traits, especially A10-centered and A03/A07-associated regions detected across developmental stages (Zhang et al., 2022a, 2022). Others may improve tissue tolerance through internal ion compartmentalization, osmotic adjustment, hydraulic regulation, and ROS protection, including SOS/NHX-related ion-homeostasis genes, P5CS-mediated proline biosynthesis, aquaporins, GPX/RABG3E, CDPK1, and related signaling candidates (Wan et al., 2017; Wassan et al., 2021; Zhang et al., 2022b, 2022, 2024) (Yang et al., 2024). Therefore, candidate loci should be prioritized when they combine stable marker-trait association, cross-stage relevance, and biological support from ion-homeostasis or stress response pathways (Wan et al., 2017; Wassan et al., 2021; Zhang et al., 2022b). Furthermore, research indicates that salt tolerance in *B. napus* is not primarily linked to sodium exclusion from shoots, suggesting that tissue tolerance and internal detoxification mechanisms are more critical breeding targets than low Na^+^ accumulation (Yong et al., 2015; Wan et al., 2017) (**Figure 1**, colored red).

Taken together, the genomic landscape of salinity tolerance supports a developmental staged framework in *B*. *napus*. Early-stage tolerance is anchored by germination, radicle growth, shoot fresh weight, seedling fresh weight, and seedling vigor loci, with repeated signals on A01, A03, A08, C06, and A10 (Wan et al., 2017, 2018; Zhang et al., 2022b). Salt-alkali and field-based tolerance add loci associated with plant height, yield components, and dynamic image-based traits, and salt tolerance rating, indicating that salinity resilience cannot be selected only from seedling survival (Zhang et al., 2022a, 2023, 2023, 2024). The strongest breeding targets are therefore not isolated SNPs, but recurrent genomic regions or validated markers that connect early vigor, ion-water balance, tissue tolerance, and yield stability. Resolving A10-centered hotspots, CO6 germination markers, and QTL-Seq STR loci remains a critical priority for future fine mapping and haplotype validation (Zhang et al., 2022a, 2022, 2022, 2024).

#### 2.2.2 Key candidate genes

Most salt-responsive QTLs in *B. napus* are enriched for genes regulating Na^+^/K^+^ homeostasis, osmotic adjustments, Ca^2+^ signaling, and ROS detoxification (Mickelbart et al., 2015).

Ion transport and Na+ detoxification: A core strategy in *B. napus* is to reduce cytosolic Na^+^ toxicity by restricting influx, exporting Na^+^, or compartmentalizing it into vacuoles (Mickelbart et al., 2015; Zhang et al., 2022b). Seedling stage GWAS/QTL regions are repeatedly enriched for transporter-related candidates, such as *HKT1* (high-affinity K^+^ transporters), Salt Overly Sensitive (SOS) pathway genes, and NHX (Na^+^/H^+^ exchanger) (Wassan et al., 2021; Zhang et al., 2022b). Components of SOS pathway, including *BnaC04g30550D* (*SOS4*), a pyridoxal kinase regulating Na^+^/K^+^ balance, and *BnaA10g29660D* (*SOS3*), are critical for maintaining ionic regulation under high salinity (Wassan et al., 2021; Zhang et al., 2022b, 2024). These work alongside *HKT1*-type transporters, which partition Na^+^ from root xylem vessels to parenchyma cells to minimize transpirational flux into shoots (Mickelbart et al., 2015). Functional evidence is strongest for NHX-mediated vacuolar Na+ sequestration, because overexpression of *AtNHX1* in *B*. *napus* enhanced salt tolerance, biomass accumulation, and yield components under salinity (Zhang et al., 2001, 2022, 2022). Therefore, *BnaC02g39600D* and other NHX-type candidates should be treated as mechanistically strong candidates, but individual Bna gene copies still require allele-level or transgenic validation in rapeseed. Additionally, the identification of *BnaA05g20580D* (*BnNHD1*) in association hotspots reinforces the importance of maintaining ion homeostasis via sodium export and vitamin B6-mediated regulation (Wassan et al., 2021; Zhang et al., 2022c).

Osmotic adjustment (compatible solutes): Salinity imposes an early shock, so loci tied to compatible solute accumulation, such as sucrose, proline, and glycine betaine, are frequently prioritized because they help maintain cellular water potential and protect proteins/membranes under ionic/osmotic stress (Wan et al., 2017). Proline pathway genes, specifically P5CS homologs (e.g., *BnaA03g18760D*), are consistently detected in major QTL intervals and are considered the primary contributors to proline accumulation under stress (Wan et al., 2017; Zhang et al., 2024). The role of P5CS1 under SS is distinct from its role in DS. While in DS it supports dehydration avoidance, under SS it mainly contributes to ion-induced osmotic buffering (Zhang et al., 2024). Other candidates include *BnaC02g31110D* (*BnCEST*), which enhances chloroplast protein stability to mitigate osmotic damage (Zhang et al., 2022c). Further regulation is provided by *bHLH112*, which binds to E-box and GCG-box elements to enhance proline accumulation and reduce ROS levels by regulating antioxidant genes (e.g., *SOD* and *POD*) (Sun et al., 2018; Yang et al., 2024).

Water transport (aquaporins): Aquaporins, notably PIP-type genes, often appear among GWAS candidates for salt tolerance (Wan et al., 2017, 2018; Wassan et al., 2021). Mechanistically, under SS they are discussed in terms of maintaining hydraulic conductivity and cellular water balance while the plant is simultaneously dealing with reduced external water potential and ion toxicity (Munns and Tester, 2008; Maurel et al., 2015). Stable QTLs have pinpoint *BnaC06g14590D* (*BnPIP2A*) and *BnaA09g44820D* (*BnTIP2*) as critical regulators of membrane water permeability during high salinity exposure (Wan et al., 2017; Wassan et al., 2021). Unlike its role in DS, where it supports dehydration avoidance, under SS it is a critical determinant of maintaining cellular turgor in the presence of excessive Na^+^.

ROS detoxification and Ca^2+^ signaling: SS-associated genomic regions are often enriched for antioxidant- and signaling related genes (Yang et al., 2024; Chen et al., 2025). This architecture fits the known salinity response where ROS, such as hydrogen peroxide (H_2_O_2_) and superoxides, accumulate downstream of osmotic and ionic stress and must be buffered to protect photosynthesis and cellular integrity/growth (Wan et al., 2017). Several candidates connect these redox defenses to Ca^2+^-linked signaling. For example, *BnaC06G0148800ZS* (*BnGPX*, AtGPX homolog), which catalyzes the reduction of toxic H_2_O_2_ to non-toxic hydroxyl compounds, while *BnaA01g26470D* (*BnRABG3E*) is a small GTPase that has been shown to reduce ROS accumulation during SS (Wan et al., 2017; Yang et al., 2024). In parallel, *C06g0171400ZS*, *AtGLR1.1* homolog is proposed to help stabilize intracellular Ca^2+^ homeostasis, and *BnaC08g36980D, AtCDPK1* homolog sits within a salinity QTL hotspot and likely links early Ca^2+^ signals to downstream adaptive responses (Vivek et al., 2013; Wassan et al., 2021; Yang et al., 2024). Finally, *BnCEST* has been described as a chlorophyll-associated factor that can enhance stress tolerance by protecting protein stability against oxidative damage (Wassan et al., 2021; Zhang et al., 2022c).

TF- and hormone-centered regulators: Multiple studies identify TF candidates particularly from the AP2/ERF, MYB, NAC, and bHLH families, in salinity-associated regions, reinforcing that salt tolerance is largely regulated at the genetic level (Wan et al., 2017; Zhang et al., 2022b). Key regulators include *BnaA01g02240D* is an AtHOS10 ortholog (an R2R3-type MYB) candidate in salt associated regions, and *BnaA03g12450D* (BnPYL8), an ABA receptor that interacts with protein phosphatases to activate stress signaling (Wan et al., 2017; Garcia-Maquilon et al., 2020; Zhang et al., 2022a). The salt-alkali and high-throughput phenotyping datasets also support *BnABA4* and *BnBBX14* as marker-supported regulatory candidates. *BnABA4* is linked to ABA-related regulation, while *BnBBX14* encodes a B-box zinc-finger regulator. Both should be described as candidate regulators from salt-alkali QTL/GWAS intervals rather than validated tolerance genes until direct functional evidence is available (Zhang et al., 2023a). Recent functional research highlights a critical stage-dependence in these regulators; for instance, the overexpression of *BnaA02g05340D* (*BnCKX5*), a cytokinin dehydrogenase, and *BnaA06g02670D* (*BnERF3*), an ethylene response factor, significantly increases sensitivity to salt and mannitol stresses specifically at the germination stage (Zhang et al., 2022b). This stage-specific response is a serious challenge for breeding and gene editing, because salt tolerance at the germination stage is poorly correlated with tolerance at the vegetative or reproductive stages (Zhang et al., 2022b). Consequently, a gene that improves seedling vigor may not necessarily enhance survival or yield if expressed prematurely or in the wrong tissue (Zhang et al., 2022b).

Post-transcriptional regulation: Natural variations in SS tolerance may involve post-transcriptional control. The RNA-binding protein Tudor-SN (TSN1) ortholog in *B. napus* (BnaaTSN1), exhibits loss-of-function polymorphisms (premature stop-codon/frameshift) in salt-susceptible lines, consistent with a role in regulating stress-adaptive gene expression at the RNA level (Dit Frey et al., 2010; Yong et al., 2015).

Overall, salinity candidate genes in *B. napus* should be selected as functional modules rather than as isolated genes. The first module includes ion-homeostasis candidates such as *SOS3*/*SOS4*, NHX-type antiporters, *NDH1*, and HKT-related transporters, which regulate Na^+^/K^+^ balance, Na^+^ export, or vacuolar sequestration (Doranie Uliaie et al., 2012; Wan et al., 2017; Wassan et al., 2021; Zhang et al., 2022b, 2022, 2024). The second module includes osmotic and hydraulic candidates such as *P5CS1*/*P5CS2* and PIP/TIP aquaporins, which maintain cellular turgor under osmotic and ionic stress (Wan et al., 2017; Wang et al., 2019a; Wassan et al., 2021; Zhang et al., 2024). The third module includes ROS, Ca^2+^, and transcriptional regulators such as *GPX*, *RABG3E*, *GLR1.1*, *CDPK1*, WRKY/MYB/NAC/bHLH genes, *BnPYL8*, *BnABA4*, and *BnBBX14*, which coordinate redox buffering, calcium signaling, hormone response and transcriptional reprogramming (Wan et al., 2017; Lohani et al., 2020; Wassan et al., 2021; Zhang et al., 2022a, 2023; Yang et al., 2024). Diagnostic markers such as the C06 germination markers and QTL-Seq STR loci are valuable for marker-assisted selection, but they should remain marker-supported targets until causal genes are resolved (Zhang et al., 2022b; Yang et al., 2024; Zhang et al., 2024).

#### 2.2.3 Pathway-level insights

At the pathway level, salinity tolerance in *B. napus* can be organized into an early osmotic phase and a later ionic-toxicity phase. An early osmotic phase characterized by a rapid response to reduced external water potential, followed by a later ionic phase in which toxic ion accumulation (mainly Na^+^ and Cl^-^) disrupts metabolism, K^+^ nutrition, and photosynthesis. This framing is explicitly supported by rapeseed salinity genetics, explaining why QTL/GWAS “hotspots” often harbor genes categorized into both water-status regulation and ion homeostasis (Munns and Tester, 2008; Wan et al., 2017). This pathway-level interpretation should be linked back to the staged QTL/GWAS framework. Early-stage loci mainly reflect osmotic adjustment, germination, root growth, and seedling biomass maintenance. Whereas salt-alkali and field-stage loci add ion-homeostasis, tissue tolerance, plant height, and yield-related responses. Therefore, salinity tolerance should be interpreted as a stage-dependent integration of ion-water balance, redox buffering, and transcriptional regulation rather than as a single Na^+^ exclusion trait (Wan et al., 2017; Wassan et al., 2021; Zhang et al., 2022a, 2022, 2022, 2024).

Stabilization of water relations (osmotic phase): Salinity imposes an immediate “water deficit” or “physiological drought” shock (Kong et al., 2025). To counteract this, rapeseed utilizes an integrated network of osmotic adjustments and aquaporin-mediated water flow (Wassan et al., 2021; Byrt et al., 2023). Loci such as *BnP5CS1* (a delta1-pyrroline-5-carboxylate synthase) are prioritized as they catalyze the rate-limiting step in proline biosynthesis, which is essential for maintaining cellular water potential (Zhang et al., 2024). PIP- and TIP-type aquaporins, such as *BnPIP2A* and *BnTIP2*, frequently appear among salinity candidates in GWAS results (Wan et al., 2017). Mechanistically, while aquaporins in drought scenarios are often associated with water uptake under limited supply, under salinity they are critical for maintaining hydraulic conductivity and cellular water movement despite the high external osmotic inhibition and internal ion toxicity (Munns and Tester, 2008; Wan et al., 2017).

Protection of ionic balance (ionic phase): A core strategy is limiting cytosolic Na^+^ levels to prevent enzymatic inhibition and cell membrane injury. Key genetic determinants include HKT1-type transporters that reduce Na^+^ delivery to shoots via xylem retrieval and NHX-type antiporters (e.g., *BnNHX1*) that support vacuolar Na^+^ sequestration and turgor maintenance (Zhang et al., 2001; Davenport et al., 2007). In *B. napus*, transgenic/functional studies using *AtNHX1* in rapeseed support the practical value of NHX-type vacuolar sequestration for salinity performance (Zhang et al., 2001). The repeated prioritization of SOS-pathway components (including *BnSOS4* locus) further links ion homeostasis to metabolic cofactor control (vitamin B6) (Shi et al., 2002b), consistent with candidate enrichment in rapeseed association intervals (Wan et al., 2017). These ion-homeostasis pathways potentially act together with the osmotic module rather than separately, because vacuolar Na^+^ sequestration, proline accumulation, and aquaporin-mediated water movement jointly maintain turgor under saline conditions (Wan et al., 2017; Zhang et al., 2024).

Prevention of secondary damage (redox and regulatory control): Downstream of osmotic and ionic stress, plants must buffer ROS accumulation that cause lipid peroxidation and membrane leakage (Shahzad et al., 2022). Candidate genes like *BnaC06G0148800ZS* (*BnGPX*, glutaredoxin/peroxidase) and *BnRABG3E* (a small GTPase) protect photosynthesis and cellular integrity by reducing superoxide and H_2_O_2_ levels (Wan et al., 2017; Paiva et al., 2019; Lin et al., 2023b; Yang et al., 2024). In addition, GWAS also consistently detects WRKY (e.g., WRKY33), NAC, and AP2/DREB family TFs in major QTL intervals. These TFs coordinate broad stress-responsive transcriptional reprogramming to prioritize stress defense over growth (Wan et al., 2017; Zhang et al., 2022b).

Epigenetic and subgenomic coordination: Recent work in rapeseed supports the idea that salinity responses also involve epigenomic level regulation, including histone marks and DNA methylation changes linked to stress-responsive transcription. Evidence suggests that the above pathways are governed by rapid epigenetic reprogramming. Chromatin accessibility and transcription change sharply within hours of salt exposure, often following a “chromatin-first, transcription later” model (Chen et al., 2025). The An subgenome typically exhibits higher levels of active epigenetic marks and more robust transcription of stress-adaptive genes compared to the Cn subgenome, which is predominantly influenced by repressive marks and transposable elements (TEs) (Gill et al., 2021; Zhang et al., 2021; Kong et al., 2025).

In summary, the repeated co-occurrence of transporter, aquaporin, antioxidant, and TF candidates across QTL/GWAS studies indicates that salinity tolerance in *B. napus* is a coordinated “ion-water-redox-regulatory” module rather than a single-gene trait (Wan et al., 2017; Wassan et al., 2021; Zhang et al., 2022b; Yang et al., 2024). The most defensible breeding targets are combinations of loci that jointly regulate ion homeostasis, osmotic adjustment, redox buffering, and developmental-stage-specific tolerance. Additional potential regulatory targets are hydraulic adjustment, including GPX/RABG3/CDPK-linked redox and Ca^2+^ signaling, and WRKY/MYB/NAC/AP2-DREB (Wan et al., 2017; Wassan et al., 2021; Zhang et al., 2022b; Yang et al., 2024; Zhang et al., 2024). However, the sucessful deployment of these targets necessitates a tissue- and developmental stage-aware approach, as germination-stage salinity tolerance is poorly correlated with tolerance at vegetative or reproductive stages, and some regulators such as *BnCKX5* and *BnERF3* can increase stress sensitivity when expressed at the wrong developmental stage (Zhang et al., 2022b). Thus, allele pyramiding, haplotype selection, and genome editing should prioritize candidates that combine stable marker-trait association, expression support, and functional relevance across developmental stages and saline field conditions.

### 2.3 Heat/high-temperature stress

#### 2.3.1 QTL/GWAS findings

*B. napus* is predominantly adapted to temperate climates and is highly sensitive to HS, particularly during reproductive development (Kourani et al., 2022; Borges et al., 2023). Elevated temperature can disrupt tapetal function, carbohydrate supply to developing pollen, and membrane stability, which together impaired pollen development and viability, increase pollen abortion, and abnormal ovule formation, leading to lower seed set and yield (Chen et al., 2021b; Mácová et al., 2022; Bakhsh et al., 2025).

Both QTL mapping and GWAS approaches have been employed to dissect the genetic basis of HS tolerance in *B. napus* under controlled and field environments. Under controlled temperatures reaching up to 35 °C during early flowering, researchers identified QTLs associated with reproductive HS tolerance traits, including 5, 8, and 7 loci for pollen sterility, sterile/aborted pods, and pod numbers on the main raceme, explaining 46.3%, 60.5%, 60.6% of phenotypic variation, respectively (Rahaman et al., 2018). In field trials under natural HS conditions (where air temperature reached approximately 35 °C), mapping of reproductive and architectural traits detected six QTLs for plant height, 11 for main raceme height, 11 for pod length, and seven for sterile/aborted pods, that together explained large proportion of variation (reported totals include 52.2% for plant height, 71.8% for main raceme height, 53.2% for pods on the main raceme, 73.5% for pod length, and 61.0% for sterile/aborted pods), highlighting that field loci capture strong environment × genotype effects during natural HS durations (Rahaman et al., 2017). Overall, loci detected under controlled conditions help reveal intrinsic reproductive thermotolerance, while field loci capture genotype-by-environment effects under fluctuating environments. Together, these studies identify yield-relevant regions can be tracked through MAS and further refined through transcriptome-supported candidate-gene analysis (Rahaman et al., 2017; 2018). These field-based insights are being further refined through associative transcriptomics (AT), which integrates marker-trait association with gene expression marker analysis to identify regulatory regions linked to reproductive resilience (Tidy et al., 2025). In addition, AT-based evidence also indentified seed-yield and seed-set associated loci on A04 and C04, including candidates near *RAX2*, *SAM1*, *GAMMA-TIP1*, and *ATPGP1*. A separate seed-yield recovery signal after transient heat was detected on C01, where marker Bo1g002670.1.2184.G occurred within a 1.02 Mb candidate interval. At present, these loci must be treated as marker-supported regions as they not yet functionally validated (Tidy et al., 2025).

In other *Brassica* crops, for instance, in *B. rapa*, a GWAS using 142 accessions exposed to 35/25 °C day/night temperatures for seven days during early flowering revealed 57 SNPs distributed across all 10 chromosomes associated with yield-related traits. Individual SNPs explained the varying degree of phenotypic variance, such as 19.06% for seed yield stress tolerance index (STI) and up to 23.43% for harvest index under control conditions (Chen et al., 2022b). Beyond, identifying discrete loci, high-throughput phenotyping reveals distinct physiological strategies between ecotypes. For example, WOR genotypes (such as BnA098 and BnA510) are frequently identified as highly heat-tolerant, maintaining seed yield and pod filling on the main raceme during stress (Tidy et al., 2025). In contrast, SOR genotypes often exhibit a greater reliance on an escape/recovery mechanism, expanding secondary racemes to compensate for initial floral abortion once cooler temperatures are re-established (Tidy et al., 2025). Similarly, in model plant, *Arabidopsis*, GWAS of more that 250 accessions subjected to a single day of 35 °C heat indentified four QTLs robustly associated with fertility reduction. These QTLs were found to be developmental-stage specific, with distinct loci identified for heat responses occurring before anthesis (meiosis stage) and after anthesis (fertilization and early embryo development). The total explained phenotypic variance of these four QTLs reached 35.7% (Bac-Molenaar et al., 2015) (**Figure 1**, colored green).

Collectively, HS QTL/GWAS findings support a staged framework in which controlled flowering-stage screens prioritize intrinsic reproductive thermotolerance, field studies capture architecture and pod-retention responses under fluctuating heat, and transcriptome-supported association approaches refine marker regions into candidate regulatory intervals. The promising breeding targets are therefore recurrent or expression-supported loci that connect pollen fertility, pod retention, seed-yield recovery, and source-sink stability, rather than isolated SNPs detected in a single environment (Bac-Molenaar et al., 2015; Rahaman et al., 2017; 2018; Tidy et al., 2025).

#### 2.3.2 Key candidate genes

Tapetum, anther development, and male fertility maintenance: Heat-associated fertility QTLs often point to genes that protect tapetal functions, regulate tapetal programmed cell death (PCD), maintain microsporogenesis, all of which are essential for viable pollen under HS. For example, *BnaA03g09160D* (a homolog of AtULI3 and located near marker *chrA03_4124353* associated with pod length), encodes a Cysteine/Histidine-rich C1 domain family protein and is linked with tapetal development, PCD regulation, and pollen sterility under heat (Zhang et al., 2014; Rahaman et al., 2017). *BnaC03g15870D* (at *chrC03_8.00* Mbp) has been associated with pollen abortion (Rahaman et al., 2017). *BnaA05g33770D* and *BnaA05g33780D* (5–6 Kb away from *chrA05_22801086*) co-localize with loci for pollen sterility and pod abortion (Rahaman et al., 2017). Notably, *BnaA05g33770D* encodes an F-box protein likely involved in ubiquitin-mediated protein degradation during heat-sensitive stage of microsporogenesis (Yang et al., 2006; Ariizumi et al., 2011; Saxena et al., 2023). *BnaC01g05710D* and *BnaC01g05800D* (near *chrC01_3055220*) belong to the protein kinase superfamily and are discussed as an important candidate genes because kinase-mediated signaling is widely implicated in pollen failure under stress (Radchuk et al., 2006). These candidates potentially regulate reproductive tissues rather involved in general HS tolerance. *BnaA03g09160D* is a stronger candidate because it is linked to tapetal development, PCD regulation, and pollen sterility near a heat-associated marker. However, *BnaC03g15870D*, *BnaA05g33770D*/*BnaA05g33780D*, and *BnaC01g05710D*/*BnaC01g05800D* are locus-supported candidate as their functional validation is required (Radchuk et al., 2006; Rahaman et al., 2017). In *Arabidopsis*, double mutant of *QUL1* and *QUL2* candidate genes exhibited enhanced sensitivity specifically to pre-anthesis HS, suggesting their role in early reproduction resilience (Bac-Molenaar et al., 2015).

Pod set, organ abortion, and embryo/seed development: A second group of candidates maps to loci controlling sterile/aborted pods, pod number, and seed set, reflecting heat impacts beyond pollen, especially fertilization success and early embryo development. For instance, *BnaC04g07360D* and *BnaC04g01250D* (flanking *chrC04_5456736* and *chrC04_rand_988002*), include candidates such as F-box family proteins and cyclic nucleotide-gated channels (CNGCs), consistent with roles in reproductive success and organ retention under stress (Yang et al., 2006; Ariizumi et al., 2011; Rahaman et al., 2017). *BnaA09g36330D* (near *chrA09_26370461*), encodes a basic helix-loop-helix (bHLH) DNA-binding protein linked to seed/pod development and dehiscence-related processes (Hudson and Hudson, 2015; Rahaman et al., 2017). *BnaC03g33590D* (pyruvate kinase family protein) is a plausible candidate where heat triggers abortion partly through disrupted energy supply during early seed/embryo development (Rahaman et al., 2017). Finally, *BnaA07g01710D* (noted from *B. rapa* near SNP *UQnapus2473*; ortholog context), is connected with embryo developmental processes that can become heat-sensitive (Chen et al., 2022b). In addition, in a combination of AT and QTL/GWAS, Tidy et al. (2025) identified several candidates associated with seed-yield and seed-set, including RAX2, SAM1, GAMMA-TIP1, and ATPGP1 on A04 and C04, and an in interval containing marker Bo1g002670.1.2184.G on C01 linked with seed-yield recovery (Tidy et al., 2025).

Heat sensing, Ca^2+^ signaling, and proteostasis protection: Heat tolerance loci also carry genes that help cells sense thermal stress and prevent protein damage through chaperones and protection of cellular proteins. For instance, *BnTR1* (membrane bound RING-type E3 ligase) is a mechanistically supported candidate because it can modulate cytosolic Ca^2+^ homeostasis and influences HSF-linked transcription, helping initiate protective heat-response mechanisms (Kourani et al., 2022; Bakhsh et al., 2025). *B. napus* contains 64 heat shock factor (HSF) genes, reflecting polyploid-driven expansion (Zhu et al., 2017). Among them, *HSFA1a* functions as a master regulator upstream of other HSFs, controlling the expression of heat shock proteins (HSPs), as well as broader stress responsive networks (including MYBs, and ABA-related genes) (Liang et al., 2019). Overall, a core HSF-HSP module underpins thermotolerance that maintains proteostasis under heat (Liang et al., 2019). For instance, *HSP18.2* plays a critical role in maintaining pollen viability and preventing growth reduction during HS (Rahaman et al., 2018), while *HSP70* family members that are markedly induced in siliques under HS, likely contribute to acquired thermotolerance by stabilizing proteins during reproductive-stage heat durations (Liang et al., 2019). *BnaA10g30100D*, a DnaJ/HSP co-chaperone with tetratricopeptide repeats, is a plausible “pod retention under heat” candidate because DnaJ/HSP systems buffer proteostasis during stress (Ma et al., 2015; Rahaman et al., 2017).

Stress signaling and hormonal control (ABA-linked regulation): Many HS phenotypes reflect not just damage, but how plants reprogram growth and reproduction under stress. This adjustment is often controlled through ABA-linked networks and phosphorylation/dephosphorylation switches. In *B. napus*, fourteen *Pyrabactin Resistance 1-Like* (*PYR1-like*) ABA receptors genes have been reported act upstream by sensing ABA and regulating downstream signaling, largely through PP2C-mediated control of kinase activity, which can reshape heat-responsive gene expression and stress acclimation (Di et al., 2018; Liao et al., 2025). In this context, *BnPYL1–2* and *BnPYL7–2* contribute to abiotic stress adaptation, including HS (Di et al., 2018). Downstream PP2C-type regulators (e.g., *BnaA07g30430D*, reported as a putative PP2C ortholog with higher expression in tolerant cultivars) are also strong candidates (Zheng et al., 2025), because PP2Cs are central tuning nodes in ABA-related signaling and wider stress-response regulation (Ghanizadeh et al., 2025).

Metabolic and circadian coordinators (source-sink, oils, and timing): Heat tolerance often correlates with the plant’s ability to maintain carbon allocation, reproductive timing, and lipid metabolism under stress. For example, *BnWRI1*, a key TF for fatty-acid biosynthesis, is often discussed because heat-driven changes in its expression can influence lipid accumulation and potentially membrane stability and seed oil traits (Rahaman et al., 2017; Ahmad et al., 2021; Kourani et al., 2022). *BnaC02g03470D* (*PRR7*) and *BnaC02g02380D* (*CHIL*) (circadian/favonoid-related candidates) are logical “buffering” genes because circadian and secondary-metabolic networks influence stress timing and ROS management (Grundy et al., 2015; Jiang et al., 2015; Qin et al., 2022). *FLC, AT5G10140* is best presented as a developmental integrator, was found on QTL6 (in *Arabidopsis* GWAS). Allelic variation in this floral integrator is genetically linked to pre-anthesis heat sensitivity, with late flowering alleles showing a significant reduction in silique length (Bac-Molenaar et al., 2015). *BnaC01g004357*, a SWEET sugar transporter, is also an important candidate in HS response, because SWEET-mediated sugar transport helps maintain source-sink balance. Under heat, disrupted sugar flux can directly limit pollen/embryo development and seed filling, making SWEET-like transporters plausible contributors to yield stability during thermal stress (Zhu et al., 2021).

Overall, HS-responsive candidates can be grouped into three evidence-based classes. Class I includes high-confidence candidates which are supported by mapping and expression dynamics, proteomics, or functional evidence. For instance, HSF-HSP-DREB2A network components, DnaJ/HSP40-type co-chaperones, *BnGLYI-3*, and selected source-sink regulators such as SWEET-type transporters (Ma et al., 2015; Rahaman et al., 2017; Zhu et al., 2017; Rahaman et al., 2018; Lohani et al., 2021; Zhu et al., 2021; Isoda et al., 2022; Kourani et al., 2022; Hu et al., 2024; Feng and Xia, 2025). Class II includes biologically strong reproductive candidates near HS-associated loci, such as *BnaA03g09160D*, *BnaC03g15870D*, *BnaA05g33770D*/*BnaA05g33780D*, *BnaC04g07360D*/*BnaC04g01250D*, and *BnaA09g36330D* (Rahaman et al., 2017; 2018; Chen et al., 2022a; Saxena et al., 2023). Class III includes marker-supported AT candidates such as *RAX2*, *SAM1*, *GAMMA-TIP1*, *ATPGP1*, and Bo1g002670.1.2184.G, which yet require functional validation (Tidy et al., 2025).

#### 2.3.3 Pathway-level insights

HS mapping studies indicate that “reproductive failure” is the primary genetic route to yield loss in *B*. *napus* (Ahmad et al., 2021; Bakhsh et al., 2025). QTLs detected under controlled flowering-stage heat are mainly associated with pollen sterility, sterile/aborted pods, and pod numbers on the main raceme (Rahaman et al., 2018). Whereas, field-detected loci also capture plant architecture, pod length, and genotypes × environment effects under fluctuating heat conditions (Rahaman et al., 2017). These findings suggest that HS-resilience relies on a conserved genetic core that protects male fertility and pod set while maintaining whole-plant source-sink dynamics (Huang et al., 2022; Kan et al., 2023; Kourani et al., 2025).

Reproductive protection is the dominant genetic mechanism (tapetum to pollen and pod set): Reproductive protection is the dominant genetic mechanism, governing the progression from tapetal development to pollen and pod set (Kourani et al., 2022; Khan et al., 2023). Several fertility-related QTLs, specifically those regulating tapetal PCD control (e.g., *BnaA03g09160D*), microsporogenesis, and protein turnover, fit into a broader thermotolerance model (Ahmad et al., 2021; Khan et al., 2023). In this model, heat disrupts tapetal function, carbohydrate supply (through the down-regulation of sucrose synthases and transporters), and cellular stability, which then cascades into pollen abortion and failed fertilization (Huang et al., 2019; Khan et al., 2023; Bakhsh et al., 2025). This logic is further supported by *Arabidopsis* GWAS, which shows that fertility reduction under 35 °C is profoundly stage-specific, with distinct genetic loci regulating pre-anthesis (meiotic) vs post-anthesis (fertilization and early embryo) sensitivity (Bac-Molenaar et al., 2015). This stage-dependency is importance for breeding. Pre-anthesis loci harbor candidates potentially involve in protecting meiosis, tapetum function, and pollen viability. In contrast, candidates on post-anthesis loci are more relevant to fertilization, embryo development, pod retention, and seed filling. To optimize HS-tolerance breeding program, selection strategies require the integration of pollen fertility, pod abortion, and seed set metrics rather than relying on as single reproductive trait (Bac-Molenaar et al., 2015; Batool et al., 2025; Kourani et al., 2025).

Proteostasis and ubiquitin-linked quality control connect “fertility loci” to heat survival: Heat-associated QTL intervals can be interpreted through a proteostasis framework, in which thermotolerant genotypes limit protein misfolding through HSF-HSP chaperone activity and ubiquitin/26S proteasome-mediated turnover (Khan et al., 2023; Batool et al., 2025). This mechanism is particularly vital in reproductive tissues, where proteome instability in the tapetum or microspore quickly translates into pollen sterility (Kourani et al., 2022; Khan et al., 2023; Bakhsh et al., 2025). This biological process is supported by identified chaperone candidates, such as HSP70, HSP90, and DnaJ-type proteins (e.g., *BnaA10g30100D*), which refold damaged proteins to maintain cellular homeostasis (Lohani et al., 2022; Kourani et al., 2025). Furthermore, F-box/E3 ligase family members (identified near major QTLs for sterile/aborted pods) are crucial for protein quality control during microsporogenesis and embryo development by targeting toxic, unfolded proteins for degradation (Rahaman et al., 2018; Lim et al., 2022). The *BnTR1* gene serves as a strong example of this pathway-level connection. As a membrane-bound RINGv E3 ligase induced by heat, it enhances thermotolerance by managing protein fate and activating stress-response pathways linked with Ca^2+^ signaling and heat shock factor (HSF) transcription (Liu et al., 2014; Huang et al., 2017; Kourani et al., 2022). This module links QTL/GWAS candidate regions to a direct cellular mechanisms. HSP70, HSP90, small HSPs, and DnaJ/HSP40 proteins buffer heat-induced protein misfolding, while F-box/E3 ligase and ubiquitin-proteosome components remove damaged proteins. These processes are especially important in tapetum, microspores, and early embryos, where proteome instability rapidly becomes pollen sterility or pod abortion (Rahaman et al., 2018; Lim et al., 2022; Lohani et al., 2022; Kourani et al., 2025).

Ca^2+^ signaling acts as an early “wiring layer” that feeds into transcription and protection: Several candidates encoding channel proteins, kinases, and other Ca^2+^-linked regulators appear to act in a coordinated manner as the plant’s first response to thermal shifts (Ahmad et al., 2021; Lohani et al., 2021; Batool et al., 2025). Ca^2+^-spikes are among the earliest stress signals, triggered by the changes in plasma membrane fluidity, and they rapidly reshape gene expression and protective programs, including the activation of HSF/HSP network and downstream metabolic adjustments (Ahmad et al., 2021; Kan et al., 2023). In rapeseed, these “wiring” components include cytosolic nucleotide-gated channels (CNGCs) and annexins (ANNs), which facilitate the inward flux of Ca^2+^ to initiate heat shock response (Ahmad et al., 2021; Lohani et al., 2021). Mechanistic work on *BnTR1* further supports the role of membrane-associated regulators in tuning Ca^2+^-linked stress signaling, as it has been shown to modulate the activity of calcium channels to influence heat acclimation and minimize growth inhibition during extreme temperature (Liu et al., 2014; Huang et al., 2017).

ABA-linked “decision making” and active reproductive reprogramming: Under HS, plant do not merely suffer physical injury; they engage in active physiological reprogramming to re-balance the trade-off between vegetative growth and reproductive survival through complex hormonal signaling and phosphorylation switches (Lim et al., 2022; Bakhsh et al., 2025). ABA contributes to stress-induced growth-reproducible reprogramming by regulating PYR/PYL-PP2C-SnRK2 signaling (Fidler et al., 2022). In the absence of stress, clade A PP2C phosphatases act as negative regulators by inhibiting SnRK2 kinases; however, during HS, ABA-bound receptors inhibit these PP2Cs, releasing SnRK2s to activate downstream TFs and ion channels (Fujii et al., 2009; Umezawa et al., 2009; Soon et al., 2012). In *B. napus*, the PYL gene family work has highlighted stress-responsive members, including BnPYL1–2 and BnPYL7-2, as critical candidates for heat acclimation and the regulation of productive outcomes (Di et al., 2018; Zandalinas et al., 2020). This active reprogramming through the ABA pathway helps the plant conserve energy and prioritize survival over immediate growth (Lim et al., 2022). This creates a breeding trade-off. ABA-linked heat acclimation can protect tissues by limiting damage and coordinating stress-response signaling, but excessive growth restraint may reduce biomass, reproductive duration, or seed filling under moderate heat. Therefore, PYL/PP2C/SnRK2-related candidates should be evaluated together with pod retention, photosynthesis, biomass, and seed yield rather than treated as universal positive regulators (Fujii et al., 2009; Umezawa et al., 2009; Soon et al., 2012; Di et al., 2018; Zandalinas et al., 2020; Fidler et al., 2022).

Metabolic stability and source-sink control of carbon flow: Metabolic stability and source-sink coordination are fundamentally linked to “pod abortion” and “seed set” loci, as heat frequently reduces fertility by making energy supplies limiting at critical stages of pollen development, fertilization, and early embryogenesis (Huang et al., 2022; Ibrahim et al., 2024). High temperatures disrupt the carbon flow by downregulating enzymes such as sucrose synthase and invertase, leading to a low sucrose-to-starch turnover and a subsequent lack of soluble carbohydrates required for pollen viability (Zandalinas et al., 2018; Parihar et al., 2025). This makes candidate genes such as pyruvate kinase (involved in energy metabolism and linked to early embryo abortion) and SWEET-like sugar transporters (crucial for sugar allocation to reproductive sinks) biologically coherent as targets for HS-responsive QTLs (Rahaman et al., 2018; Isoda et al., 2022; Wang et al., 2022a; Hu et al., 2024). Furthermore, rapeseed studies confirm a major metabolic rewiring under HS characterized by the transcriptional deregulation of the BnWRI1 pathway (Huang et al., 2019, 2022). This suppression of *BnWRI1* inhibits the *de novo* fatty acid biosynthesis pathway, preventing the successful incorporation of carbohydrates into triacylglycerols (TAG) and resulting in significantly decreased seed oil accumulation (Huang et al., 2019; Batool et al., 2025; Kourani et al., 2025). The above source-sink regulation could be considered as a bridge between reproductive protection and seed-quality resilience. Heat can reduce pollen viability by limiting carbohydrate supply, but it can also reduce seed oil accumulation by disrupting WRI1-linked fatty-acid biosynthesis and carbon allocation to developing seeds. Thus, SWEET transporters, pyruvate kinase candidates, and BnWRI1-related pathways should be evaluated as a combined carbon-allocation module under HS (Huang et al., 2019; Hu et al., 2024; Kourani et al., 2025; Parihar et al., 2025).

Redox regulation and reactive-carbonyl detoxification are integral to reproductive resilience: Heat stress also induces metabolic dysfunction, including ROS accumulation and methylglyoxal-mediated reactive carbonyl stress (Ahmad et al., 2021). High temperatures elevates ROS and concurrently increase reactive carbonyl stress, specifically through the accumulation of methylglyoxal (MG), a potent cytotoxin primarily generated during the accelerated carbohydrate and lipid metabolism that occurs in seeds under thermal stress (Yan et al., 2016). In *B. napus* seeds, a Glyoxalase I protein (specifically the *BnGLYI-3* variant) has been identified as a critical heat-induced responder. Comparative proteomics reveals that while this protein expression is diminished in the heat-sensitive cultivars, it is significantly elevated in thermotolerant seeds, identifying MG detoxification as a primary layer of thermotolerance biology (Yan et al., 2016). Functional characterization shows that BnGLYI-3 variant (from tolerant lines) possesses specific amino acid variations at position 9 and 93 that enhance its catalytic efficiency compared to the sensitive BnGLYI-2 version (Yan et al., 2016). This detoxification capacity fits into a broader, integrated model of reproductive protection, proteostasis, and metabolic homeostasis (Lohani et al., 2022). By clearing MG, the plant prevents the denaturation of vital proteins and the disruption of cellular membranes during the precise developmental windows when reproduction is most vulnerable (Kourani et al., 2022). Furthermore, this pathway is intrinsically linked to the broader redox network. For example, seeds overexpressing BnGLYI-3 maintain significantly higher Superoxide Dismutase (SOD) and Ascorbate Peroxidase (APX) activities, which collectively quench ROS bursts and allow the reproductive machinery, such as pollen tube growth and early embryogenesis, to remain functional under severe HS (Yan et al., 2016; Lohani et al., 2021; Kourani et al., 2022).

In conclusion, HS tolerance in *B*. *napus* is fundamentally a mechanism of reproductive-resilience, structurally supported by proteostasis, source-sink regulation, lipid metabolism, and redox detoxification. Because, heat-associated QTLs mainly target pollen sterility, steril or aborted pods, pod number, plant architecture, and yield-related traits under controlled and field conditions (Rahaman et al., 2017; 2018). QTL/GWAS loci define reproductive and architectural risk points, while candidate genes define modules for tapetum and pollen protection, proteostasis, ABA-linked stress regulation, source-sink balance, lipid metabolism, and redox detoxification. These modules determine pod retention, seed set, seed-yield recovery, and oil accumulation under heat (Rahaman et al., 2017; Zhu et al., 2017; Rahaman et al., 2018; Lohani et al., 2021; Kourani et al., 2025). The most useful breeding targets will be loci or candidates where marker association, expression response, physiological relevance, and yield-related performance converge. However, there are several unresolved HS-associated intervals on A03, A05, C01, C03, A04, and C04 can be prioritized for fine mapping, haplotype testing, and functional validation across controlled and field heat environments (Tidy et al., 2025).

### 2.4 Cold/freezing stress

#### 2.4.1 Ecotype differentiation and physiological basis of cold tolerance

Cold, chilling, and freezing stress substantially restrict the geographical distribution and productivity in *B. napus* (Luo et al., 2021; Qin et al., 2022; Wu et al., 2025; Zheng et al., 2025). Long-term exposure to distinct winter environments has driven the evolution of three major ecotypes, such as winter oilseed rape (WOR), semi-winter oilseed rape (SWOR), and spring oilseed rape (SOR) (Rakow, 2007; Wei et al., 2017a; Wrucke et al., 2019). WOR types possess strong vernalization requirements and enhanced freezing tolerance, enabling overwinter survival in colder climates, whereas SWOR types exhibit moderate cold tolerance with partial vernalization dependence, and SOR types lack vernalization requirements and are generally insensitive (Wang et al., 2011). These ecotypic differences highlight the close association between cold acclimation, developmental regulation, and freezing survival in *B. napus* (Horvath et al., 2020).

Cold resilience in *B*. *napus* can be summarized in three linked stages. For example, low-temperature germination and early vigor, cold acclimation before freezing, and survival or recovery after freezing injury (Qin et al., 2022; Zheng et al., 2025). These stages, shares some molecular components, including ABA signaling, ROS protection, and membrane stabilization, but they are not identical traits (Chao et al., 2021; Zheng et al., 2025). Therefore, candidates detected for low-temperature germination should not automatically be treated as overwintering genes, and genes associated with freezing damage should be evaluated separately from those controlling vernalization or flowering time adaptation (Horvath et al., 2020; Qin et al., 2022).

#### 2.4.2 QTL/GWAS findings

In QTL/GWAS studies under cold/freezing in *B. napus*, mapping results often points to two key findings, such as (i) cold tolerance is highly polygenic and environment-dependent, and (ii) certain chromosomes recur as hotspots among the different experiments and population. Phenotyping is often based on traits that capture freezing injury at the cellular and whole-plant level, such as REL, an indicator of membrane damage) and composite indices like freezing damage index (FDI). Because they provide quantitative measures of freezing induced injury that can be compared across genotypes (Wrucke et al., 2019; Chao et al., 2021; Zheng et al., 2025). In a QTL mapping study using a RIL population derived from cold-tolerant (GZ hui, WOR) and cold-sensitive (10B, SWOR) lines identified 28 QTLs controlling FDI (Qin et al., 2022). Moreover, a major effect locus associated with frost tolerance was identified on chromosome A02 under controlled simulated freezing conditions (Wrucke et al., 2019). Field-based mapping analyses further detected 38 significant loci and one major and stable QTL on chromosome A04 (Wrucke et al., 2019). Further, combining QTL mapping and WGS analyses led to the identification of an “anchor” region that is consistently detected is qFDI.C02.1 (located around 0.5-5.9 Mb on chromosome C02), identified as a co-detected locus across environments and markers, supporting the idea that at least some cold-tolerance determinants are reproducible rather than purely site-specific (Qin et al., 2022). In addition, this QTL overlapped with homoeologous exchange (HE), a meiotic process in allopolyploid organisms where chromosomes from different ancestral subgenomes (homeologs) pair and exchange segments (Mason and Wendel, 2020), emphasizing the role of structural variations (SVs) in CS responses. Notably, qFDI.C02.1 locus is absent in the cold-sensitive SWOR line 10B due to HE, directly linking genomic SV with phenotypic cold sensitivity (Hu et al., 2021; Qin et al., 2022). The qFDI.C02.1 region should be emphasized as more than a marker-trait association. Because this locus overlaps with homologous exchange and is absent in the cold-sensitive SWOR line 10B, it provides structural-variant-supported evidence for cold sensitivity. This makes qFDI.C02.1 one of the strongest genomic region for further validation, particularly for separating copy-number, presence/absence, and expression effects (Hu et al., 2021; Qin et al., 2022). Mapping efforts have also explored morphological adaptations to cold, such as leaf petiole angle (LPA), identifying 45 significantly associated QTLs that link leaf architecture to overwintering success (Hu et al., 2021). Furthermore, recent GWAS for seedling-stage cold tolerance grades has pinpointed a major peak on A08 centered on the *BnaA8.MYB60* transcription factor (Wu et al., 2025). Variation in this locus regulate cold resilience by modulating malondialdehyde (MDA) content, thereby protecting membrane integrity under sub-zero temperatures (Wu et al., 2025).

GWAS has expanded the catalog of cold-responsive loci. For example, GWAS analysis using diverse panels (157–228 accessions) detected ten QTLs linked with REL, collectively explaining ~43% the phenotypic variation (Wrucke et al., 2019). A GWAS using a panel of 399 accessions (primarily WOR, canola) identified 13 significant markers across nine chromosomes that are targeting freezing survival scores and chlorophyll fluorescence traits (e.g., Fv/Fo) after cold acclimation, showing that top loci can relate to both freezing injury/tolerance and photosynthetic performance under CS (Chao et al., 2021). Beyond initial freezing survival, GWAS has identified five loci specifically associated with the genetically distinct process of deacclimation (Horvath et al., 2020). Candidate genes in these regions, including *Phytochrome Kinase Substarte 4* (*PSK4*) and *Early Flowering 6* (*ELF6*), suggest that the intensity of freezing tolerance loss during warm spells is heavily governed by light signaling and circadian regulatory networks (Horvath et al., 2020). More recently, integrative approaches have strengthened confidence in GWAS signals by pairing association mapping with expression evidence. For instance, a study using 289 core germplasms from 36 countries and employed a combination of GWAS with RNA-Seq analyses to refine candidate genes underlying freezing tolerance. From association mapping data, they identified a total of 45 significant SNPs distributed on the chromosomes across both A and C subgenomes, and were linked with REL as a marker trait (Zheng et al., 2025). This type of GWAS and RNA-Seq integration is especially valuable prioritizing the cold-responsive candidate genes. Candidate genes should therefore be ranked by 1) convergence among genomic co-localization with REL, FDI, Fv/Fo, or survival traits, 2) differential expression during cold or freezing treatment, and 3) biological function in membrane stability, ROS detoxification, CBF/DREB transcription, or vernalization-linked development (Wrucke et al., 2019; Horvath et al., 2020; Chao et al., 2021; Qin et al., 2022; Zheng et al., 2025). Crucially, low temperature tolerance extends beyond overwinter survival. Early-stage vigor and low-temperature germination are equally genetically tractable traits.

QTL mapping in combination with GWAS work has identified loci on chromosomes A07 (q0WRTA07 and q0WRLA07), A09 (qLTGA9-1), and C01 (qLTGC1-1) for low-temperature germination traits, and seed vigor (Luo et al., 2021; Zhu et al., 2021; Qin et al., 2022; Zheng et al., 2025). These findings indicate that early-stage performance can contribute meaningfully to enhance tolerance in cold-prone regions. High-resolution QTL-seq has further refined the seed vigor landscape, delineating a 341.86 kb interval on A09 and a 1.31 Mb interval on C01 for low-temperature germination (Zhu et al., 2021) (**Figure 1**, colored navy blue). The cold QTL/GWAS landscape therefore supports two complementary breeding routes. The first route targets overwintering survival through freezing-damage and REL loci such as A02, A04, and C02 (Wrucke et al., 2019; Qin et al., 2022; Zheng et al., 2025). The second route targets early establishment through low-temperature germination loci on A07, A09, and C01 (Luo et al., 2021; Zhu et al., 2021; Qin et al., 2022; Zheng et al., 2025). These routes should be treated separately in breeding because strong overwintering capacity may not always predict rapid low-temperature germination.

#### 2.4.3 Key candidate genes

Membrane stabilization and lipid remodeling: Maintenance of membrane phase stability is essential for CS tolerance. Cold injury often begins with membrane rigidification and phase transitions, so genes involved in lipid composition and membrane maintenance are frequently nominated. Several CS linked QTLs on chromosome A07 harbor genes involved in lipid remodeling and membrane protection (Horvath et al., 2020). *ALA12*, an aminophosphate ATPase, contributes to phospholipid flipping across membranes, preserving bilayer asymmetry and fluidity under CS (Gomès et al., 2000). Another gene encoding lipoxygenase (LOX) enzyme that participates in fatty acid oxygenation, modulating membrane lipid composition and signaling during cold acclimation (Kang et al., 2021; Zheng et al., 2025). Additional candidates from cold-associated intervals strengthen this membrane-centered interpretation. *FAD5* orthologs, ALA-type phospholipid transporters, *LOX3*, *COR413-PM4*, *LTI29*/dehydrin, and HXXXD-type acyltransferase candidates point to lipid remodeling, membrane stabilization, and cryoprotection. ALA12 and LOX candidates are linked to A07 cold-associated QTLs and lipid remodeling functions, while *BnaA02g29330D* encodes HXXXD-type acytransferase is linked to cell wall or cuticular modification under CS (Wrucke et al., 2019; Horvath et al., 2020; Kang et al., 2021; Zheng et al., 2025). These genes are potentially involved in a membrane-protection module linked to REL, FDI, and post-freezing recovery. Because, REL and FDI measure freezing induced membrane injury, and cold-driven membrane disruption interacts with ROS damage during freezing and recovery (Wrucke et al., 2019; Chao et al., 2021; Zhang et al., 2025b). Most remain predicted or expression-supported candidates in *B*. *napus* and require validation.

ROS detoxification and cellular protection: Freezing and freeze-thaw cycles generate oxidative bursts, so loci near cold-tolerance peaks often include antioxidant and stress-protection candidates (e.g., catalase/ROS detox machinery and chaperones/proteostasis functions). Two catalase genes, such as *BnaA07g11360D* (*CAT1*) and *BnaA07g11370D* (*BnCAT3.A07*), detoxify hydrogen peroxide generated during freezing-induced ROS production (Chao et al., 2021). These are biologically plausible because limiting oxidative damage helps protect membranes and proteins during freezing stress (Chao et al., 2021; Raza, 2021). The ROS-protection group should be expanded to include APX1-like candidates, glutathione/peroxidase-related genes, HSP100/ClpB, ubiquitin-conjugating enzymes, and protein phosphatase candidates detected near freezing-associated regions. *APX1*-like candidates, including *BnaA09g49190D* and *BnaC04g24470D*, are supported by RNA-Seq evidence in leaf tissues and are linked to H_2_O_2_ detoxification during freezing stress (Zheng et al., 2025). *CAT1* and *CAT3*, including *BnaA07g11360D* and *BnaA07g11370D*, detoxify H_2_O_2_ generated during freezing-induced ROS production (Chao et al., 2021), while *HSP100*/*ClpB* supports proteostasis by dissolving protein aggregates during freezing-thaw cycles (Hong and Vierling, 2000). Protein phosphatase candidates, including *PP2C*/*HAB1* and PP2C49, are supported by RNA-Seq evidence in tolerant cultivars and are linked to freezing-stress responses (Zheng et al., 2025). These genes connect oxidative detoxification with proteostasis during freeze thaw-cycles. CAT1, CAT3, APX1, and HSP100/ClpB should therefore be framed as stronger candidates because their functions directly explain lower membrane leakage and improved cellular survival after freezing.

Hormone signaling and growth restraint: ABA signaling-related genes are crucial for cold adaptation, growth restraint, and survival programming under low-temperature (Raza, 2021; Kourani et al., 2022). Key candidates near QTLs (qLTGA9–1 and qLTGC1-1) include cytochrome P450 family proteins, involved in ABA degradation, and Phosphatase 2C (PP2C) genes such as *BnaA09g39770D (AtPP2C49)* and *BnaA07g30430D (AtHAB1)*, which regulate ABA signal transduction (Shinozaki and Yamaguchi-Shinozaki, 2007; Zhu et al., 2021; Zheng et al., 2025). Cold-associated ABA and growth-regulatory candidates can be considered as balancing genes rather than simple tolerance genes. PP2C/HAB1-type genes, *NCED6* is linked to ABA biosynthesis and *ARR18* to cytokinin signaling in freezing-associated intervals (Fiebelkorn et al., 2018; Zhu et al., 2021; Zheng et al., 2025). *PSK4* and *FLC* orthologs connect cold response with growth regulation, phytochrome-linked development, vernalization requirement, and flowering-time adaptation (Horvath et al., 2020; Hu et al., 2021; Qin et al., 2022). However, excessive growth restraint can delay flowering or reduce early biomass, so these candidates therefore require environment-specific evaluation. In addition, *PSK4*, associated with phytochrome signaling and auxin/BR responsiveness, links light perception and hormonal growth control to cold-regulated development (Kim et al., 2017). Calcium signaling also contributes to cold perception, as evidence by EF-hand Ca^2+^-binding proteins (Volkov et al., 2006) and membrane magnesium transporters induced under low temperatures (Qin et al., 2022).

Cold-response transcriptional control: A central class of candidates in cold tolerance are transcriptional regulators and their downstream targets, especially AP2/DREB-type regulators presents the core transcriptional module through linking the canonical cold-response networks underlying cold acclimation (Zhao et al., 2016; Zheng et al., 2025). These candidates are crucial because they can act as “switches” that coordinate broad defense-related networks rather than single enzymes (Chao et al., 2021; Zheng et al., 2025). In *B. napus*, AP2/DREB family members are highly enriched near cold-associated QTLs, reflecting strong conservation of these transcriptional regulators during CS (Horvath et al., 2020). The transcriptional layer should include CBF/DREB, *WRKY33*/*WRKY25*, *ATAF2*, *NF-YA9*, and *NF-YB3* candidates. CBF/DREB genes from the central cold-acclimation switch by activating COR and LEA-type downstream genes and CBF overexpression in *B*. *napus* induced cold-responsive orthologs such as Bna115 and Bna28 and improves freezing tolerance (Jaglo et al., 2001; Savitch et al., 2005; Horvath et al., 2020; Zheng et al., 2025). WRKY33/WRKY25, ATAF2, NF-YA9, and NF-YB3 are supported by GWAS/RNA-Seq overlap or cold responsive expression, indicating that they act as broader transcriptional regulators rather than single-enzyme candidates (Wei et al., 2022; Zheng et al., 2025). WRKY and NAC-family candidates may connect cold response with ROS, defense signaling, and structural gene regulation, while NF-Y candidates may provide broader stress-transcription control (Kim et al., 2024; Zheng et al., 2025). These genes should not be considered independently, instead, they should be presented as a transcriptional hierarchy that links cold perception to downstream membrane, osmotic, and antioxidant protection.

Developmental regulators tied to overwintering (FLC neighborhood): In *B. napus*, cold tolerance often intersects with vernalization-linked development. For instance, candidates identified at a significant marker localized on Cnn_random are related to vernalization and FT (Horvath et al., 2020). One such gene, encoding Phytochrome Kinase Substrate 4 (PSK4), is involved in phytochrome signaling and is highly expressed in response to auxin and brassinolide, indicating its significant role in growth regulation. Phytochrome signaling is crucial in cold-mediated gene expression (Kim et al., 2017). Another gene that was detected in LD region is Early Flowering 6 (ELF6), which is involved in circadian cycle and influences acclimation processes (Fowler et al., 2005; Horvath et al., 2020). BnaFLC orthologs (e.g., *BnaC02g00490D*) link low-temperature tolerance with vernalization requirements, reinforcing the ecotype-level differences between WOR and SWOR lines established in the drought-adaptation trade-off (Hu et al., 2021; Qin et al., 2022). *FLC*, *PSK4*, and *ELF6* provide a developmental layer that distinguishes cold tolerance from simple cellular stress resistance (Horvath et al., 2020; Hu et al., 2021; Qin et al., 2022). These genes help explain why WOR, SWOR, and SOR ecotypes differ in freezing survival, vernalization requirement, and flowering behavior. Their breeding value depends on matching alleles to the target production system (Wang et al., 2011; Horvath et al., 2020). Their breeding value depends on matching alleles to the target production system. Strong vernalization-linked freezing tolerance may be useful in winter rapeseed, but the same developmental program may be undesirable in spring or short-season environments.

Overall, high-confidence cold-responsive candidates could be prioritized by convergence of QTL/GWAS evidence, expression response, and pathway role. These include CBF/DREB transcriptional regulators and COR/LEA/dehydration cryoprotective genes, which form the central cold-acclimation module in *B*. *napus* (Jaglo et al., 2001; Savitch et al., 2005; Zheng et al., 2025). They also include CAT/APX/HSP100-linked ROS and proteostasis genes, lipid-remodeling genes such as FAD5, ALA12, LOX3, and COR413-PM4, and developmental regulators such as FLC, PSK4, and ELF6 (Horvath et al., 2020; Chao et al., 2021; Zheng et al., 2025). However, many individual Bna gene copies remain predicted or expression-supported rather than experimentally validated.

#### 2.4.4 Pathway-level insights

CBF-centered cold acclimation as the “core module”: A widely supported model is that CBF TFs bind CRT/DRE promoter elements and activate cold-responsive (COR) genes, providing osmoprotection, membrane stabilization, and improved stress resilience. This CBF-mediated COR activation is explicitly appreciated as a central cold acclimation route in canola-focused work and is also supported by earlier mechanistic evidence that the CBF cold-response pathway operates in *B. napus* (Jaglo et al., 2001; Chao et al., 2021). The CBF module should be presented as the transcriptional entry point, not the whole cold-tolerance mechanism. CBF/DREB genes activate COR, LEA, dehydrin, and osmoprotective genes such as *Bna115* and *Bna28* while improving freezing tolerance (Jaglo et al., 2001; Savitch et al., 2005). However, the final phenotype depends on whether this transcriptional response is coordinated with membrane-lipid remodeling, antioxidant protection, and developmental timing, including FAD5/ALA/LOX-type lipid candidates, CAT/APX-linked ROS detoxification, and FLC/PSK4/ELF6-linked developmental regulators (Horvath et al., 2020; Chao et al., 2021; Zheng et al., 2025).

Membrane remodeling and ROS protection are coupled: Cold-driven membrane disruption and oxidative stress reinforce each other. For instance, membrane damage worsens ROS leakage and signaling imbalance, while ROS accelerates lipid peroxidation. That is why mapping peaks often co-locate candidates for lipid remodeling and antioxidant defenses. These functions work together to preserve cellular integrity during freezing and recovery (Chao et al., 2021; Raza, 2021). This coupling explains why cold-associated QTLs often contain both lipid-related and antioxidant-related genes. Membrane rigidification increases leakage and ROS formation, while ROS accelerated lipid peroxidation unless detoxification systems such as CAT, APX, GPX, and HSP-linked proteostasis are activated (Chao et al., 2021; Zheng et al., 2025). Therefore, REL and FDI phenotypes should be interpreted as combined outputs of membrane stability, redox buffering, and protein protection, rather than as simple injury score (Wrucke et al., 2019; Chao et al., 2021; Qin et al., 2022; Zheng et al., 2025).

Cold tolerance is unusually intertwined with development: Unlike drought or salinity (where FT effects are often “escape strategies”), freezing tolerance is tightly linked with vernalization response and overwinter physiology, which helps explain why developmental regulators (e.g., FLC-associated components) frequently appear in cold-tolerance discussions (Huang et al., 2021; Raza, 2021). This developmental control creates a major breeding trade-off. Strong cold acclimation and vernalization responsiveness can improve overwintering survival, but they may delay flowering or reduce adaptation to spring-sown or short-season systems (Wang et al., 2011; Horvath et al., 2020). Conversely, fast germination and rapid early growth under low temperature may support establishment but they do not necessarily predict freezing survival. Because, low-temperature germination and early-vigor loci are mapped separately from freezing-damage and overwintering-related loci (Qin et al., 2022; Zheng et al., 2025). Therefore, CS breeding should separate three target profiles, such as low-temperature germination, cold acclimation, and overwintering survival.

A comprehensive cold-resilient framework in *B*. *napus* integrates precise cold perception through membrane and Ca^2+^-linked signaling, transcriptional activation through CBF/DREB, WRKY, NAC, and NF-Y regulators. It should also include cellular protection through COR/LEA/dehydrin proteins, lipid remodeling, antioxidant enzymes, and HSP/proteostasis system, together with developmental tuning through FLC, PSK4, and ELF6 (Horvath et al., 2020; Chao et al., 2021; Zheng et al., 2025). This framework links loci to pathways and phenotypes and identifies the main research gaps. These gaps include resolving causal genes within recurrent A02, A04, C02, A07, A09, and C01 loci. They also include testing individual Bna homeologs, validating candidate alleles across WOR, SWOR, and SOR backgrounds, and evaluating whether improved freezing survival carries penalties for flowering time, biomass, or seed yield (Horvath et al., 2020; Qin et al., 2022; Zheng et al., 2025).

### 2.5 Flooding/waterlogging stress

#### 2.5.1 QTL/GWAS findings

WS limits oxygen diffusion into the rhizosphere, so roots rapidly face hypoxia (or, under severe flooding, anoxia). Oxygen deprivation suppresses mitochondrial respiration, limits ATP production, and disrupts carbohydrate metabolism, and ROS can spike during both flooding and post-stress reoxygenation (Sasidharan et al., 2017; Li, 2022). These metabolic disturbances necessitate adaptive strategies that balance energy conservation, redox homeostasis, growth regulation to ensure survival under prolonged or transient flooding (Voesenek and Bailey‐Serres, 2015; Meng et al., 2022). Plants typically cope via two contrasting survival strategies under WS, such as low-O_2_ quiescence syndrome (LOQS) and low-O_2_ escape syndrome (LOES) (Voesenek and Bailey‐Serres, 2015). LOQS is characterized by growth suppression, metabolic down-regulation, and energy conservation, traits commonly associated with WS tolerant *Brassica* genotypes. In contrast, LOES involves rapid shoot elongation to restore contact with the atmosphere, a strategy more typical of flood adapted species but energetically costly and generally unfavorable in *B. napus.* Thus, in *Brassica* crops, understanding the genetic basis of LOQS-associated traits is central to improving WS tolerance (Mickelbart et al., 2015; Voesenek and Bailey‐Serres, 2015).

To identify WS tolerance related loci through QTL/GWAS approaches, physiologically relevant traits include seedling death rate (SDR), relative root length (RRL), relative hypocotyl length (RHL), and relative fresh weight (RFW). These traits collectively reflect early vigor, biomass retention, and recovery capacity following flooding (Ding et al., 2020a; Wang et al., 2020b). Using a RIL population of about 200 lines grown under controlled WS conditions, 17 consensus QTLs associated with SDR were identified. Among these, cqSDR.C3-2, cqSDR.C8-1, and cqSDR.C8–2 were repeatedly detected across environments, with cqSDR.C3–2 emerging as a major locus, explaining up to 23.3% of phenotypic variation. This QTL is likely linked to root system regulation and seedling survival under hypoxia, consistent with its strong and stable phenotypic effects (Wang et al., 2020b). Further analyses using reciprocal introgression lines derived from WS sensitive (GH01) and WS tolerant (ZS9) parents detected 66 QTLs linked with RRL, RHL, and RFW. Importantly, six shared loci (qWTA7-1, qWTA7-2, qWTC1, qWTC6, qWTC7-1, qWTC7-2) were common across both tolerant and sensitive backgrounds, which highlights their robustness and suitability for MAS (Ding et al., 2020a). GWAS has broadened the scope. A panel of 520 accessions yielded 26 significant SNPs associated with SDR, mainly on chromosomes A04 and C04. Notably, these GWAS hits did not overlap the RIL QTLs. However, these results were consistent with WS tolerance being regulated by polygenic and environment-sensitive variations, with major loci detected in bi-parental populations and numerous small-effect loci revealed by GWAS (population type, stress protocol, and recovery conditions) (Wang et al., 2020b). Another GWAS using 300 accessions, focused on shoot fresh weight (SFW) under WS and recovery conditions, identified 43 significant SNPs, with a strong enrichment on chromosome C07, pointing to C07 as a recurring region for biomass maintenance and post-flood recovery (Guo et al., 2022). This chromosome consistently emerges as a key region associated with post-flooding recovery and biomass maintenance. Recent advances in UAV-based field phenotyping have improved the resolution and throughput of WS tolerance assessment by capturing canopy biomass dynamics and recovery trajectories or “response curves” over time. This approach reported many loci, and emphasized a smaller set of highly reliable loci, including 13 loci consistently detected across multiple indices of WS, highlighting how temporal, non-destructive phenotyping detects dynamic stress responses that are difficult to capture through conventional measurements (Li et al., 2022) (**Figure 1**, colored pink) (**Supplementary Table 1**). In a comparative genomic context, GWAS of 387 Arabidopsis accessions under complete submergence identified 35 significant SNPs associated with seven physiologically important traits, including survival score, chlorophyll retention, and starch-related responses (Meng et al., 2022). This study highlighted mitochondrial *ACONITASE3* as a candidate for acclimation to submergence stress. However, results from a model plant can be used to guide candidate-pathway interpretation, rather a direct breeding markers for B. napus. Because, rapeseed WS studies currently prioritize SDR, RRL, RHL, RFW, SFW, and recovery-related loci under hypoxia and post-flood recovery (Ding et al., 2020a; Wang et al., 2020b;Guo et al., 2022).

Taken together, the B. napus WS QTL/GWAS landscape supports a recovery-centered framework in which stable SDR loci, shared root and hypocotyle growth loci, and C07-enriched SFW associations. In addition, UAV-derived temporal loci should be prioritized for marker development, fine mapping, and candidate-gene validation (Ding et al., 2020a; Wang et al., 2020b;Guo et al., 2022; Li et al., 2022).

#### 2.5.2 Key candidate genes

In **Supplementary Table 1**, multiple markers show association with WTI and SDR traits, especially the C07-enriched WTI block and A04 SDR markers (Wang et al., 2020b;Guo et al., 2022). However, these markers are unresolced candidates that can be subjected as breeding regions for MAS or haplotype testing, not as validated genes. In contrast, loci for RFW, RRL, and RHL contain more interpretable candidates, including oxidoreductase-related genes (e.g., OTLD1, RPN1B, XRN3, CBL3 and FERONIA), and GPI-anchored adhesion-related genes (Ding et al., 2020a). These candidates potentially fit the main WS modules of redox control, protein/RNA turnover, Ca^2+^ signaling, cell-wall sensing, and recovery growth.

ROS detoxification/redox control (hypoxia + re-oxygenation): Effective ROS management is essential under hypoxia and reoxygenation. In stable QTL clusters, several candidates encode antioxidant and redox-regulatory proteins (Ding et al., 2020a). *BnCAT3.A07*, a catalase gene, contributes to hydrogen peroxide detoxification under WS. *BnaA02g07580D*, encoding a glutaredoxin, supports thiol-based redox regulation during hypoxia and especially during re-oxygenation (Ding et al., 2020a; Li et al., 2022). Additional peroxidase superfamily members (e.g., *BnSKS17.A07*) further reinforce the antioxidant capacity under WS (Ding et al., 2020a). The enriched QTL/GWAS findings further supports this module because RFW and RHL loci include oxidoreductase-related candidates such as *BnaA07g29460D*, *BnaA07g31960D*, *BnaC06g35970D*, and *BnaC07g38970D*. However, their causal roles of these redox-associated candidates under hypoxia and re-oxygenation remain unconfirmed and require expression or functional validation (Ding et al., 2020a).

Protein/RNA turnover and cellular housekeeping: Hypoxia induces extensive protein and RNA damage, necessitating the effective degradation pathways. *BnOTLD1.A07* (ubiquitin-related)*, BnRPN1B.C01* (proteasome subunit), and *BnXRN3.C06* (RNA exonuclease), are implicated in turnover of damaged proteins/RNAs and reset metabolism during prolonged WS and recovery (Ding et al., 2020a).

thylene signaling and hypoxia response regulators: Comparative transcriptome analyses between WS tolerant genotype 126 and sensitive genotype 85 revealed the stronger induction of *BnEIN3* and *BnERF73* in the tolerant lines aligns with the ERF-VII-mediated hypoxia sensing mechanism detailed in Section 3.4. In addition, the tolerant genotype also exhibits better physiological stability (Li et al., 2025a). Thus, *BnEIN3* and *BnERF73* are additionally expression-supported hypoxia-response candidates, whereas marker-only ERF or ethylene-related genes in QTL intervals are still considered as predicted candidates.

Ca2+/energy signaling nodes: Calcium signaling is also critical. UAV-GWAS candidate mining highlighted a *BnaA02g07660D*, encoding CIPK15 in a robust region. *CIPK15*, together with *BnaCBL3.C07* integrate Ca^2+^ signals with downstream metabolic reprogramming (Ding et al., 2020a; Li et al., 2022). This function aligns with the CIPK15-SnRK1-linked “energy/sugar sensing” role, that is well characterized in flooding tolerance in rice (Guo et al., 2019), broadly connected to hypoxia adaptation.

Membrane and lipid remodeling (damage limitation + recovery): Maintenance of membrane intengrity under hypoxia is regulated by genes (identified through GWAS peaks on C07) squalene monooxygenase and GDSL esterase/lipase, which modulate the lipid composition and membrane stability during WS. These functions are particularly important during recovery, when membrane damage accumulates due to oxidative stress (Guo et al., 2022).

Overall, WS-responsive candidates fall into three distinct evidence-based tiers. Class 1 includes stronger convergence candidates, such as *BnEIN3*, *BnERF73*, *CIPK15*, *BnaCBL3.C07*, *BnCAT3.A07*, glutaredoxin-related candidates, and peroxidase-related candidates. Because, they connect QTL/GWAS evidence with transcriptomic, redox, Ca^2+^, or hypoxia-response functions (Ding et al., 2020a; Guo et al., 2022; Li et al., 2022). Class II includes QTL-supported recovery candidates such as *OTLD1*, *RPN1B*, *XRN3*, *FERONIA*, GPI-anchored adhesion-related genes, oxidoreductase-related candidates, squalene monooxygenase, and GDSL esterase/lipase genes (Ding et al., 2020a). Class III includes marker-supported WTI and SDR regions, especially the C07 WTI block and A04 SDR markers, where causal genes remain unresolved (Wang et al., 2020b;Guo et al., 2022).

#### 2.5.3 Pathway-level insights

Under WS conditions, the most consistent phenotype is not rapid shoot elongation, but survival followed by recovery. This fits a quiesence-leaning strategy in which tolerant genotypes restrict unnecessary growth, conserve carbohydrates, maintain root and shoot biomass, and restart growth after drainage (Voesenek and Bailey‐Serres, 2015; Sasidharan et al., 2017; Wang et al., 2020b;Yemelyanov et al., 2023). In this framework, QTLs for SDR, RRL, RHL, FW, and waterlogging tolerance index (WTI) converge on four interacting biological modules, such as hypoxia/ethylene signaling, energy and sugar sensing, redox protection, and membrane/cell-wall recovery (Ding et al., 2020a; Wang et al., 2020b;Guo et al., 2022; Li et al., 2022).

Hypoxia/ethylene signaling: Hypoxia caused by waterlogging reduces mitochondrial respiration and ATP production. Ethylene accumulates rapidly under flooded conditions and acts upstream of hypoxia-responsive transcriptional reprogramming (Sasidharan et al., 2017; Hartman et al., 2021; Li et al., 2025a). Transcriptomic evidence showing stronger induction of *BnEIN3* and *BnERF73* in tolerant genotypes support their role as regulatory candidates linking oxygen limitation to adaptive root growth and stress-gene activation (Li et al., 2025a).

Energy and sugar sensing: Energy signaling provides the mechanistic bridge between hypoxia perception and survival. The UAV-GWAS-supported candidate *BnaA02g07660D*/*CIPK15* and the QTL supported *BnaC07g40720D*/*CBL3* point to a Ca^2+^-linked energy-sensing module (Ding et al., 2020a; Li et al., 2022). This module likely connects early flooding signals to SnRK1-related sugar and ATP management, thereby supporting anaerobic metabolism while preventing carbon exhaustion (Wurzinger et al., 2018; Li et al., 2022). This is a high-priority module for validation because it connects a mapped locus with a clear physiological phenotype, such as biomass retention and recovery after waterlogging (Li et al., 2022).

Redox protection: Redox buffering is required because both hypoxia and reoxygenation generate ROS. QTL-supported redox candidates, including *BnCAT3.A07*, *BnaA02g07580D* glutaredoxin, peroxidase-related genes, and oxidoreductase candidates such as *BnaA07g29460D*, *BnaA07g31960D*, *BnaC06g35970D*, and *BnaC07g38970D*, link mapped loci to antioxidant protection (Ding et al., 2020a; Li et al., 2022). Their likely function is to limit lipid peroxidation, protein oxidation, and membrane leakage during stress and recovery (Ding et al., 2020a; El-Khateeb et al., 2023; Zhang et al., 2025c). These candidates are therefore best classified as QTL/GWAS-prioritized and functionally plausible, but most still require direct validation through expression perturbation, near-isogenic lines, and genome editing (Ding et al., 2020a; Li et al., 2022).

Membrane/cell-wall recovery: Recovery depends on membrane, cell-wall, and protein/RNA homeostasis. Candidates genes such as *BnaA07OLTD1*, *BnaC01g09980D*/*RPN1B*, and *BnaC06g36330D*/*XRN3* suggest that tolerant genotypes actively remove damaged proteins and RNAs during prolonged waterlogging and recovery (Ding et al., 2020a). The C07-enriched signals, together with candidates such as *BnaC07g32280D*/*FERONIA*, indicate that cell-wall sensing, adhesion, and membrane repair are also central to post-flood recovery (Ding et al., 2020a; Guo et al., 2022; Li et al., 2024). This helps explain why C07 repeatedly appears as a recovery-associated region in GWAS for SFW and waterlogging tolerance index (Guo et al., 2022; Li et al., 2022). For breeding, these findings indicate that single-gene selection is unlikely to provide durable waterlogging tolerance. MAS should prioritize stable loci such as cqSDR.C8-2, shared qWT loci, and C07 recovery-associated haplotypes (Ding et al., 2020a; Wang et al., 2020b;Guo et al., 2022), whereas, genomic selection may better capture the many small-effect loci detected by GWAS and UAV-based temporal phenotyping (Wang et al., 2020b;Li et al., 2022). For genome editing, the defensible targets are regulatory nodes that connect mapped loci to recovery phenotypes. For example, *CIPK15*/*CBL3* for energy signaling, *EIN3*/*ERF73* for ethylene-hypoxia signaling, and selected redox or recovery genes for reoxygenation tolerance (Ding et al., 2020a; Li et al., 2022, 2025). A key trade-off must be considered. For instance, strong quiescence can improve survival under flooding, but excessive growth suppression may reduce post-stress biomass accumulation and yield. Conversely, escape-type elongation may restore oxygen contact but can deplete carbohydrates and weaken recovery in rapeseed (Voesenek and Bailey‐Serres, 2015; Sasidharan et al., 2017; Hartman et al., 2021).

Future work should therefore validate these candidates across developmental stages, flooding duration, soil types, and post-stress recovery windows, while measuring yield and oil-quality penalties (Wang et al., 2020b;Li et al., 2022; Zhang et al., 2025c). Although pathway-level convergence is strong, causal validation of individual Bna gene copies remains limited (Ding et al., 2020a; Guo et al., 2022; Li et al., 2022, 2025). A list of significant QTLs/SNPs collected from QTL mapping and GWAS studies targeting promising traits of all five stresses are presented in **Figure 1**.

## 3 Integration of QTL/GWAS-prioritized genes with transcriptomic, proteomic, and metabolomic evidence

### 3.1 Drought stress

To survive water scarcity, rapeseed initiates an integrated hierarchy of morphological adaptations and molecular signaling networks. These strategies range from drought avoidance, involving ABA-mediated stomatal closure and cuticular wax deposition, to DS tolerance, characterized by the accumulation of osmoprotectants like proline and the activation of ROS-scavenging system (Gupta et al., 2020; Fang et al., 2022; Lu et al., 2024). Because DS resistance is a polygenic trait governed by hundreds of small-effect loci, moving beyond phenotypic selection requires a multi-omics approach (Hu and Xiong, 2014). By integrating QTL mapping and GWAS with transcriptomics, proteomics and metabolomics, we can bridge the gap between genetic variation, such as favorable alleles in the *NCED3* or *NF-YA7* loci, and the functional metabolic shift required for climate resilience in *B. napus* (Lu et al., 2024; Wang et al., 2024a). A comprehensive summary of these QTL/GWAS-prioritized genes, their expression profiles, and biological roles across all five climate-related stresses is provided in **Table 1**.

**Table 1:** Expression profiles of QTL- and GWAS-prioritized genes responsive to climate-related stresses in *Brassica napus* L.

Stress	*B. napus* ID	*A. thaliana* ID	Gene expression regulation	Biological role	Citation	Evidence class
Drought	BnaA09g26040D	AT1G30500.2 (NF-YA7)	Up-regulated in drought sensitive lines (RNA-Seq/qRT-PCR), across tissues	Negatively regulates tolerance by repressing the BnaABF3/4 pathway, inhibiting ABA-induced stomatal closure, enhancing stomatal conductance and transpiration rate.	(Wang et al., 2024a)	Functionally supported
	BnaA10g21040D	AT5G11450.1 (PPD5)	Down-regulated in drought tolerant accessions (RT-qPCR)	Modulates H_2_O_2_ accumulation in guard cells, reducing expression promotes stomatal closure and enhances drought resistance.	(Shahzad et al., 2021)	Functionally supported
	BnaC06g14590D/BnaA03g17020D/BnaA03g17030D	AT3G53420.2/AT2G37170.1 (PIP2A/B)	Up-regulated in drought-tolerant lines (qRT-PCR)	Enhances drought tolerance through improved mineral transport, water uptake, and hydraulic conductivity.	(Shinozaki and Yamaguchi-Shinozaki, 2007; Khanzada et al., 2020)	Expression-supported
	BnaA05g05760D	AT2G39800.4 (P5CS1)	Up-regulated (RNA-Seq)	Increases synthesis of osmoprotectant L-proline, aiding in osmotic adjustment and ROS scavenging during drought.	(Guo et al., 2019; Shahsavari et al., 2025)	Functionally supported
	BnaC04g50970D	AT2G46680.1 (HB-7)	Up-regulation (qRT-PCR) in a drought-tolerant line	Induced by water deficit and ABA, involved in the DS response by regulating ABA-mediated pathways.	(Khanzada et al., 2020)	Expression supported
	BnaA02g00370D	AT5G10140.1 (FLC)	Up-regulation (RNA-Seq) in high yielding cultivars; Tissue seed	Regulates flowering time, with higher expression correlating to drought avoidance and enhanced yield under DS.	(Salami et al., 2024a)	Expression supported
Salt	BnaA04g08360D	AT5G37850.2 (SOS4)	Up-regulated, targeted for induction	Encodes pyridoxal kinase (SOS4), involved in vitamin B6 synthesis and regulation of Na^+^ and K^+^ homeostasis, enhancing salt tolerance.	(Wassan et al., 2021)	Expression supported
	BnaC08g36980D	AT1G18890.1 (CDPK1)	Up-regulated in tolerant lines (qRT-PCR), leaf tissue	Activated by Ca^2+^ influx during SS, CDPK1 links Ca^2+^ signaling to ion channel regulation, supporting salt tolerance.	(Wassan et al., 2021)	Expression supported
	BnaA03g01900D	AT5G06150.1 (CYC1BAT)	Up-regulated (GWAS/RNA-Seq/qRT-PCR)	Homolog of SOS3, senses salt-induced Ca^2+^ and activates SOS2 in SOS pathway to regulate Na^+^ exclusion and maintain ion balance.	(Ulfat et al., 2020; Zhang et al., 2022b)	Expression supported
	BnaA02g05340D	AT5G21482.1 (CKX7)	Up-regulated (RNA-Seq)	Overexpression increases salt sensitivity at the germination stage, affecting early salt tolerance mechanisms.	(Wassan et al., 2021; Zhang et al., 2022b, 2023)	Heterelogous proof-of-concept
	BnaA03g18760D, BnaA05g05760D, BnaC03g72600D, BnaC04g05620D	AT2G39800.4 (P5CS1)	Up-regulated (RNA-Seq/qRT-PCR), root tissue	enhances proline biosynthesis, which act as a compatible osmolyte to maintain osmotic balance under SS.	(Long et al., 2015; Wang et al., 2019a; Zhang et al., 2024)	Expression supported
	BnaC04g55570D, BnaA04g03460D	AT3G55610.1 (P5CS2)	Up-regulated (RNA-Seq/qRT-PCR), root tissue	Involved in proline biosynthesis under SS, contributing to osmotic regulation.	(Wang et al., 2019a)	Expression supported
	BnaA02g24470D, BnaA06g36020D	AT5G46350.1 (WRKY8)	Up-regulated (RNA-Seq), root tissue	Expressed under SS (24–72 hrs), potentially involved in managing SS responses.	(Wang et al., 2019a, 2022)	Expression supported
Cold	BnaC07g27590D; BnaA03g13820D, BnaA04g17420D, BnaA05g12160D, BnaC03g16740D, BnaC04g14500D, BnaC04g41050D	AT2G38470.1 (WRKY33); AT2G30250.1 (WRKY25)	Up-regulated (GWAS/RNA-Seq overlap)	WRKY33 is frequently upegulated during CS and plays key role in cold adaptation	(Wei et al., 2022)	Expression supported
	BnaA03g11520D/BnaC02g13250D	AT5G55180.1 (AFP12/13)	Up-regulated in freezing-resistant cultivars (RNA-Seq), leaf tissue	Involved in synthesis of proteins related to anti-freezing, enhancing cold tolerance	(Zheng et al., 2025)	Expression supported
	BnaA09g49190D/BnaC04g24470D	AT1G07890.6 (APX1), AT5G63620.1 (HER2)	Up-regulated (RNA-Seq), leaf tissue	Alleviate freezing-induced oxidative stress by detoxifying H2O2, crucial for maintaining cellular integrity.	(Zheng et al., 2025)	Expression supported
	BnaC08g37760D	AT1G17420.1 (LOX3)	Up-regulated (RNA-Seq), leaf tissue	Involved in lipid metabolism, catalyzing the oxygenation of polyunsaturated fatty acids, and promoting stress response via oxylipins.	(Zheng et al., 2025)	Expression supported
	BnaC03g43050D, BnaA08g22720D	AT3G22930.1 (CML11), AT1G18210.2 (CML27)	Up-regulated in freezing-resistant cultivars (RNA-Seq), leaf tissues	Key components of Ca^2+^ signaling pathway, important for stress signal transmission and response.	(Zheng et al., 2025)	Expression supported
	BnaC03g37030D; BnaA02g31900D, BnaA06g39340D, BnaA09g04520D, BnaC02g48560D, BnaC07g29150D, BnaC09g03940D	AT3G11020.1 (DREB2B); AT5G25610.1 (RD22)	Up-regulated in fast germinating genotypes (RNA-Seq)	AP2/ERF TFs regulate multiple signaling pathways, crucial for regulating stress response and cold tolerance.	(Guo et al., 2019; Luo et al., 2021)	Expression supported
	BnaA08g30910D, BnaA08g30950D, BnaAnng34260D	AT4G25490.1 (DREB1B/CBF1), AT4G25480.1 (DREB1A/CBF3), AT4G25470.1 (DREB1C/CBF2)	Down-regulated (RNA-Seq), leaf tissue	CBFs are involved in upregulating cold acclimation genes, enhancing freezing tolerance despite down-regulation.	(Horvath et al., 2020)	Expression supported
	BnaC03g03740D	AT5G08790.1 (ATAF2)	Up-regulated (RNA-Seq/GWAS overlap)	Regulates the expression of structural genes involved in CS pathways.	(Zheng et al., 2025)	Expression supported
	BnaC03g41820D	AT3G20910.1 (NF-YA9)	Up-regulated (RNA-Seq/GWAS overlap)	Enhances cold and other stress tolerance by modulating gene expression related to stress responses.	(Zheng et al., 2025)	Expression supported
	BnaC03g39380D	AT4G14540.1 (NF-YB3)	Down-regulated (RNA/GWAS overlap)	Negative regulator of stress responses; downregulation increases cold susceptibility.	(Zheng et al., 2025)	Expression supported
	BnaA01g24990D	AT3G21560.1 (UGT84A2)	Up-regulated in cold-tolerant cultivar Longyou-7 (qRT-PCR/RNA-Seq), tissue growth point	Regulates cold-stress tolerance by influencing the dynamics of cold-responsive gene expression.	(Ma et al., 2019)	Expression supported
	BnaA07g10370D	AT1G22600.1 (LEA)	Up-regulated in fast germinating genotypes (RNA-Seq)	Stabilizes cellular structures under cold and dehydration stress, improving osmotic stress tolerance.	(Luo et al., 2019)	Expression supported
	BnaA01g11770D, BnaA01g02080D	AT4G21930.1, AT4G35320.1 (CDPK15/DUF584, CDPK5)	Up-regulated in both freezing-tolerant and susceptible genotypes (Transcriptomics)	Induces Ca^2+^ signaling to trigger expression of genes involved in freezing tolerance.	(Wei et al., 2022)	Expression supported
	BnaA03g24370D, BnaC03g28980D	AT4G10380.1 (NIP5;1)	Up-regulated (RNA-Seq)	Aquporins involved in regulating water uptake and transport during germination under stress conditions	(Luo et al., 2019)	Expression supported
	BnaA07g30430D, BnaA09g39770D	AT1G72770.3 (PP2C/HAB1), AT3G62260.1 (PP2C49)	Up-regulated in tolerant cultivars (RNA-Seq), leaf tissue	Positively regulates protein phosphatase (e.g., serine/threonine) activities to improve freezing stress response.	(Zheng et al., 2025)	Expression supported
	BnaC02g00490D, BnaC09g46500D	AT5G10140.1 (FLC)	Up-regulated in WOR (T193) compared to SWOR (T268) (RNA-Seq/qRT-PCR), leaf petiole tissue	Genetic variation in FLC orthologs influences vernalization requirement, linking flowering regulation with cold adaptation.	(Hu et al., 2021; Qin et al., 2022)	Functionally supported
Heat	BnaHSP70 orthologs	AtHSP70s	Up-regulated (RNA-Seq), tissue silique, seed filling stage	Prevent protein aggregation and assist in protein refolding, conferring thermotolerance during reproductive development.	(Yu et al., 2014; Liang et al., 2019)	Expression supported
	BnaHSP18.2 orthologs	AtHSP18.2/21/101	Up-regulated	Maintain protein quality and repair heat-induced damage, essential for acquiring thermotolerance.	(Liang et al., 2019; Ling et al., 2021)	Functionally supported
	BnaA03g55280D, BnaA10g25000D, BnaC02g02300D,BnaAnng01320D	AT5G05410.1, AT5G05410, AT5G05410.1 (DREB2A/2B)	Up-regulated (Transcriptome/AS variants)	DREB2A/B TFs regulate downstream heat-responsive genes; alternative splicing variants enhance functional protein accumulation under HS.	(Sakuma et al., 2006b; Ling et al., 2021)	Expression supported
Waterlogging	BnaC03g41740D	AT3G20770.1 (EIN3)	Up-regulated in tolerant genotype 126 (RNA-Seq)	Rapid EIN3 accumulation under flooding triggers ethylene signaling, serving as a key regulator of hypoxia responses.	(Li et al., 2025a)	Expression supported
	BnaC06g24360D	AT1G72360.3 (ERF73)	Up-regulated in tolerant genotype 126 (RNA-Seq)	ERF-II TFs involved in hypoxia sensing and adaptive responses such as adventitious root formation.	(Li et al., 2025a)	Expression supported

#### 3.1.1 Osmolyte metabolism

Genes involved in osmotic adjustment play a pivotal role in DS tolerance by maintaining cellular integrity under water deficit (Sun et al., 2020; D’oria et al., 2022). For instance, *BnP5CS1* is markedly upregulated in *B. napus* under DS conditions (D’oria et al., 2022). Integrated multi-omics verify that *BnP5CS1* transcriptional programs correlate directly with the massive proline accumulation (Wang et al., 2019a; D’oria et al., 2022; Shahsavari et al., 2025). These studies based on transcriptomics, proteomics and biochemical analyses, confirm that increased transcript levels lead to higher P5CS enzymatic activity while a concomitant decrease in proline dehydrogenase (PDH) expression suppresses catabolism to stabilize the osmoprotectant pool (Kubala et al., 2015; Wang et al., 2019a). Furthermore, these genes are located within drought-associated QTL regions, with *BnP5CS1* specifically linked to eQTLs that regulate the metabolic reprogramming of proline biosynthesis in response to DS (Wang et al., 2019a; D’oria et al., 2022).

#### 3.1.2 Aquaporins and water transport

Aquaporins facilitates water transport across cell membranes, a process crucial for drought avoidance (Khanzada et al., 2020; Chen et al., 2022a). The gene *BnPIP2*, is linked to water transport and drought response as evidenced through high-resolution GWAS analysis (Khanzada et al., 2020). Transcriptomic analysis of *B. napus* under DS shows *BnPIP2* is highly expressed, while integrated proteomic data suggested that stress-responsive vesicle trafficking and post-translational phosphorylation are essential for the precise membrane translocation and water-channel activity of these proteins (Luo et al., 2015; Zhu et al., 2016; Khanzada et al., 2020). Further functional validation revealed that overexpression of the related aquaporin gene *BnPIP1* in tobacco significantly enhanced water transport efficiency and reduced wilting, confirming its conserved role in dehydration avoidance across species (Zhu et al., 2016; Kim et al., 2024). These findings are consistent with multiple QTL and GWAS results that identified loci on chromosomes A05 and C01, associated with water transport and root architecture (Khanzada et al., 2020; Lu et al., 2024).

#### 3.1.3 TF-mediating DS response

TFs are central regulators of DS responses. Integrated multi-omics studies further validate the regulatory hub that are identified through QTL mapping and GWAS. For example, transcriptomics confirms the stress-induction of *DREB2A* (*BnaC03g37030D*), while proteomics highlights its stabilization through inhibition of NRD-domain phosphorylation (Sakuma et al., 2006a, 2006; Shinozaki and Yamaguchi-Shinozaki, 2007; Kim et al., 2024). Similarly, omics data confirm that ABA-dependent factors, such as ABF/AREB, require SnRK2-mediated phosphorylation to trigger the metabolic shifts, specifically enhancing proline biosynthesis essential for osmotic adjustment (Liu et al., 2015; Li et al., 2021b; Kim et al., 2024). Both *DREB2A* and *ABF*/*AREB* genes are enriched in drought-responsive QTL regions on chromosomes C02 and A08 (Khanzada et al., 2020; Hosseini et al., 2025). These results further validate *Bna.FLC.A10* as a high-confidence target for manipulating the balance between developmental phenology and stress resilience (Fletcher et al., 2016).

#### 3.1.4 Hormonal signaling and ABA pathway

Hormonal regulation, particularly through ABA signaling, is crucial for DS adaptation, triggering stomatal closure, and enhancing root water uptake (Ilyas et al., 2021; Shahzad et al., 2021; D’oria et al., 2022). Transcriptomics and proteomics identify the systemic CLE25-BAM signaling module, which moves from roots to leaves to drive the rapid metabolic induction of *BnNCED3* and increase ABA levels (Gupta et al., 2020; Kim et al., 2024; Sato et al., 2024). Several ABA pathway genes identified in QTL and transcriptomic studies, including *AFP4*, *ABR1*, and *XERICO* (an E3 ligase promoting ABA accumulation), are essential for maintaining water balance (Wu et al., 2020; Ilyas et al., 2021). Integrated proteomics further reveals that SnRK2/OST1-mediated phosphorylation of BnaPPD5.A10 and *βCA1* specifically modulates H_2_O_2_ status in guard cells to facilitate drought avoidance via precise stomatal regulation (Wang et al., 2016; Shahzad et al., 2021).

In summary, the integration of QTL mapping and GWAS with transcriptomic, proteomic, and metabolomic data provides a systemic-level view of the genetic architecture underlying DS tolerance in *B. napus* (Hu and Xiong, 2014; Zhi et al., 2024). Key genes, such as *BnP5CS1*, *BnPIP2*, and *DREB2A*, regulate essential processes like increased proline accumulation for osmotic adjustment, increased cellular water permeability, and the post-translational stabilization of transcriptional hubs (Yu et al., 2005; Fàbregas and Fernie, 2019; Kim et al., 2024; Sato et al., 2024; Shahsavari et al., 2025). Thus, within the drought module, *BnP5CS1* and selected PIP aquaporins represent strong convergence candidates because they are supported by mapping, expression, physiological function, and metabolite-level outcomes. Whereas, *DREB2A*/*ABF*/*AREB*, *BnaPPD5*, and other ABA-related regulators are only either expression or proteomics-supported regulatory candidates, and their individual Bna copies are yet to be functionally validated.

### 3.2 Salt/salinity stress

Both transcriptomic and molecular studies reveal that *B. napus* adopts a coordinated set of strategies to cope with salinity, focusing primarily on rapid signal transduction, the maintenance of ionic homeostasis, and osmotic adjustment (Ulfat et al., 2020; Dai et al., 2024). The integration of QTL mapping, GWAS with transcriptomic, proteomic and metabolomic approaches provides a comprehensive view of the complex genetic networks and metabolic pathway, such as lipid and phenylpropanoid metabolism, that regulate these adaptive responses (Shu et al., 2022; Wang et al., 2022c; Zhang et al., 2023a; Zhou et al., 2025). This multi-omics synthesis facilitates the prioritization of candidate genes, allowing for a deeper understanding of how candidate loci functionally contribute to stress tolerance at the cellular and tissue levels (Zhang et al., 2024; Wang et al., 2025).

#### 3.2.1 Ionic homeostasis and Na^+^ exclusion

The SOS pathway is a core regulatory mechanism that controls Na^+^ exclusion at the plasma membrane (Ali et al., 2023). The SOS module (SOS3-SOS2-SOS1) facilitates Na^+^ efflux, which transcriptomic profiling shows is significantly upregulated in *B. napus* roots to maintain ionic balance under saline conditions (Feng et al., 2020; Ali et al., 2023; Liang et al., 2024). In *B. napus*, genes such as *BnaSOS3 BnaSOS3* (CBL4) and *BnaSOS4* are essential components of this pathway (Ulfat et al., 2020; Dai et al., 2024). Proteomic studies further reveal that their activity is supported by up-accumulated proteins involved in protein metabolism and damage repair (Jia et al., 2015). *BnaSOS3* perceives salt-induced cytosolic Ca^2+^ signals (Ismail and Horie, 2017; Yang and Guo, 2018; Köster et al., 2019; Dai et al., 2024; Liang et al., 2024), triggering downstream Na^+^ exclusion while metabolomic shifts in lipid metabolism (e.g., linoleic acid) and the accumulation of proline stabilize cellular membranes against ion toxicity (Shi et al., 2002a; Shah et al., 2021; Wang et al., 2022c). Through QTL and GWAS, *BnaSO3* has been prioritized as a key candidate gene, functionally validated by its essential role in maintaining a favorable Na^+^/K^+^ ratio during SS (Shah et al., 2021; Zhang et al., 2022b).

#### 3.2.2 Ca^2+^ signaling and early SS response

Early SS responses involve ionic shock, characterized by rapid Ca^2+^ influx and membrane depolarization (Peng et al., 2025). Beyond the induction of *AtGLR1.1* homologs, Na^+^ perceived by cell-surface GIPC sphingolipids that gate Ca^2+^ channels to trigger a cytosolic surge (Yu et al., 2020). In *B. napus*, this surge activates CDPKs (Ca^2+^-Dependent Protein Kinases, including *BnaCDPK1* homologs) and the SOS1 pathway, while proteomic studies identify up-accumulated defense proteins, such as Catalase-3 (CAT3) and HSP90, to mitigate early oxidative damage (Ludwig et al., 2004; Jia et al., 2015; Shah et al., 2021; Shu et al., 2022; Peng et al., 2025). Root transcriptome profiling of *B. napus* reveals the upregulation of various kinase signaling components, including MPKKs, RPKs, and CIPKs, at both early and late phases of SS (Feng et al., 2020; Wang et al., 2022b). Simultaneously, metabolomic analysis shows a concomitant shift in sugar and lipid metabolism, specifically the accumulation of linoleic acid, to stabilize cellular membranes and maintain ionic balance stress phases (Wang et al., 2022b, 2022).

#### 3.2.3 Osmotic adjustment and proline biosynthesis

Another key adaptive mechanism revealed by metabolomic profiling is osmotic adjustment, primarily through the different accumulation of proline and soluble sugars to maintain cell turgor (Shu et al., 2022; Dai et al., 2024; Zhou et al., 2025). Transcriptomic analysis of *B. napus* exposed to NaCl stress identifies *BnaP5CS1* and *BnaP5CS2* as crucial genes whose upregulation drives this biosynthesis in both leaves and roots (Wang et al., 2019a, 2025). Concurrently, the reduced expression of *BnProDH* helps divert metabolic flux toward osmoprotection, while proteomic data confirms that the up-accumulation of associated defense proteins and chaperones mitigates oxidative damage (Xue et al., 2009; Jia et al., 2015; Wang et al., 2019a, 2025). Additionally, exogenous application of Poly(γ-glutamic acid) has been shown to synergistically induce these biosynthetic genes (*BnaP5CS1* and *BnaP5CS2*) and repress degradation pathways, further enhancing proline accumulation and improving overall biomass and salinity tolerance in *B. napus* (Lei et al., 2016).

#### 3.2.4 TF-mediated regulation

TFs are central regulators of the SS response in *B. napus*, with transcriptomic and proteomic data showing they coordinate the expression of stress-related genes involved in ion transport, ROS detoxification, and hormone signaling (Shu et al., 2022; Ali et al., 2023). Transcriptome profiling reveals that WRKY TFs, such as *BnaWRKY40* (*AtWRKY33* homologs), are among the most significantly upregulated genes in salt-affected roots and leaves (Zhang et al., 2022b), regulating downstream genes involved in ion homeostasis and ABA signaling (Yan et al., 2014; Lan Thi Hoang et al., 2017; Gul et al., 2022; Wang et al., 2022b). The WRKY family has been consistently prioritized in QTL and GWAS studies as a key contributor to salt tolerance (Zhang et al., 2024). The NAC and MYB families also play significant roles. For example, *BnaNAC1* regulates membrane stability and senescence, while MYB TFs modulate metabolomic shifts in secondary metabolite biosynthesis (such as phenylpropanoids) and ROS scavenging (Yong et al., 2014; Kayum et al., 2015; Wang et al., 2022b; Zhou et al., 2025). Proteomic analysis validates these regulatory networks through the high accumulation of associated defense proteins like CAT3 and HSP90 (Shu et al., 2022; Wang et al., 2022b). Furthermore, the ERF family, including *BnaERF3* (*AtERF* homolog), was identified via GWAS to regulate ethylene-mediated stress signaling pathways, with functional confirming validation confirming its role in the SS response (Zhang et al., 2022b, 2023).

#### 3.2.5 Cytokinin signaling and stress response

Interestingly, GWAS and transcriptomic analyses prioritized *BnaCKX5*, a cytokinin oxidase gene, as a candidate gene regulator of salinity response (Zhang et al., 2022b, 2023). *BnaCKX5* regulates cytokinin homeostasis, a process supported by metabolomic profiling showing that SS markedly decreases endogenous cytokinin levels to modulate root architecture and developmental signaling (Feng et al., 2020; Zhang et al., 2023a). Overexpression of *BnaCKX5* in *Arabidopsis* results in increased sensitivity to salt and mannitol stress during germination, indicating that cytokinin levels serve as a metabolic “switch” rather than directly enhancing tolerance (Zhang et al., 2022b, 2023). This reflects a complex multi-omics regulatory network where root transcriptome data confirms *BnaCKX5* is significantly induced by salt and ABA to prioritize stress signaling over plant growth (Zhang et al., 2022b, 2023).

#### 3.2.6 Integration of pathways related to Na^+^ exclusion, osmotic stabilization, and transcriptional rewiring

The tolerance of *B. napus* to SS arises from the interplay of several mechanisms, including Na^+^ exclusion via the SOS pathway, osmotic stabilization through proline accumulation, and transcriptional rewiring involving key TFs (Wang et al., 2019a; Ali et al., 2023; Dai et al., 2024). Transcriptomic and metabolomic analyses confirm that these responses are supported by significant shifts in lipid, amino acid, and carbohydrate metabolism to maintain cellular turgor and membrane integrity (Wang et al., 2022c; Zhou et al., 2025). Proteomic profiling further reveals that up-accumulated defense proteins and chaperones, such as HSP90 and CAT3, mitigate oxidative damage and sustain protein metabolism (Shu et al., 2022). QTL mapping and GWAS provide invaluable insights into the genetic architecture of these responses, identifying major QTL hotspots on chromosomes A10 and A07 that control key traits related to SS tolerance (Lang et al., 2017; Zhang et al., 2022b). These findings underline the polygenic nature of adaptation, where the coordinated regulation of phenylepropanoid biosynthesis and ion homeostasis stabilizes the plant under stress (Wang et al., 2022c; Zhou et al., 2025).

In summary, QTL/GWAS, transcriptomic, proteomic, and metabolomic evidence converge on an ion-water-redox-regulatory module for salinity tolerance in *B*. *napus* (Wan et al., 2017; Wassan et al., 2021; Zhang et al., 2022b, 2022; Yang et al., 2024; Zhou et al., 2025). The strongest candidates are those linking stable marker-trait associations with stress-responsive expression and pathway function, including SOS/NHX/HKT-related ion-homeostasis genes, P5CS1/P5CS2-mediate proline biosynthesis, PIP/TIP aquaporins, and GPX/RABG3E/CDPK-linked redox and Ca^2+^ signaling (Zhang et al., 2001; Wan et al., 2017; Wang et al., 2019a; Wassan et al., 2021; Zhang et al., 2022b, 2022, 2024). However, most individual *Bna* copies remain express-supported or marker-supported rather than experimentally validated. Thus, these genes should be prioritized for allele mining, haplotype testing, and stage-specific validation before genome editing or in breeding program (Wan et al., 2017; Zhang et al., 2022b; Yang et al., 2024; Zhang et al., 2024).

### 3.3 Cold and heat stress

Temperature tolerance in *B. napus* is although two related but distinct multi-omics modules. Under CS, QTL/GWAS loci associated with REL, FDI, freezing survival, and low-temperature germination converge with transcriptomic and metabolomic evidence for CBF/DREB-COR signaling, membrane-lipid remodeling, ROS detoxification, and vernalization-linked developmental regulation (Wrucke et al., 2019; Horvath et al., 2020; Chao et al., 2021; Luo et al., 2021; Qin et al., 2022; Zheng et al., 2025). Under HS, reproductive QTLs and transcriptomic/proteomic datasets converge on HSF-HSP-DREB2A-mediated proteostasis, protein turnover, source-sink regulation, and redox detoxification (Yan et al., 2016; Rahaman et al., 2017; 2018; Huang et al., 2019; Liang et al., 2019; Huang et al., 2022; Kourani et al., 2022; Wang et al., 2023b). Thus, cold tolerance is mainly resolved through membrane stability and developmental acclimation, whereas heat tolerance is mainly resolved through reproductive protection and proteome stability.

#### 3.3.1 Cold/freezing stress tolerance

CS stress, including cold, chilling, and freezing, represents a significant environmental constraint for *B. napus*, influencing its geographical distribution and yield stability (Lei et al., 2019; Wei et al., 2022). Integrated multiomics analyses reveal that CS tolerance is driven by coordinated transcriptional, proteomic, and metabolic reprogramming, and QTL/GWAS-prioritized candidate genes often map to these same signaling and protective modules (Luo et al., 2021; Mehmood et al., 2021; Qin et al., 2022; Zheng et al., 2025). CS perception begins at the plasma membrane, where cold-induced rigidification triggers Ca^2+^ influx (potentially via sensors such as COLD1) and activates CDPK and MAPK cascades (including BnaCDPK1), which converge on cold-responsive transcriptional networks (Lei et al., 2019; Mehmood et al., 2021; Wei et al., 2021; Kim et al., 2024). Central to this response is the ICE-CBF-COR pathway (Lei et al., 2019; Mehmood et al., 2021). In this cascade, *CBF*/*DREB1* TFs (e.g., *BnaCBF1*, *BnaCBF2*, and *BnaCBF3*) binds DRE/CRT elements to induce broad suites of COR genes (Zarka et al., 2003; Vazquez-Hernandez et al., 2017; Dong et al., 2020; Mehrotra et al., 2020; Abdullah et al., 2022; Vyse et al., 2022; Kim et al., 2024). In parallel, TF families such as WRKY, NAC, ERF (e.g., *BnERF70*), MYB (e.g., MYB44), and NF-YB reinforce stress adaptation by integrating ROS signaling with hormonal control, particularly ABA (and sometimes JA) pathways. These transcriptional outputs promote the accumulation of cryoprotective proteins (COR and LEA proteins; e.g., *BnaLEA3, BnaA.LEA6.a*) and osmolyte biosynthesis enzymes such as P5CS for proline production (Czernik et al., 2020; Kim et al., 2024; Wang et al., 2024).

Consistent with these transcriptional shifts, the proteome shows broad remodeling characterized by the accumulation of HSPs, chaperones, and the ROS-detoxifying enzymes (SOD, POD, CAT) that stabilize proteins and maintain redox balance (Mehmood et al., 2021; Hu et al., 2025). Some aquaporins (e.g., BnaPIP2) are downregulated, especially at germination, potentially limiting uncontrolled water flux and dehydration during freezing (Luo et al., 2019). Metabolomic and lipidomic adjustment further support cellular stability through membrane lipid desaturation (e.g., via FAD3 and ADS2; increasing linoleic and α-linolenic acids) (Fang et al., 2021) and the accumulation of compatible solutes (glucose, fructose, sucrose) and proline) for osmoprotection (Lei et al., 2019; Niu et al., 2021; Liu et al., 2022, 2024).

Finally, regulatory control of CS is more complex than a single linear pathway (Zheng et al., 2022; Kim et al., 2024). Although ICE1 was proposed as a direct upstream regulator of CBF3/DREB1A, later analyses indicate ICE1 does not directly control CBF3 transcription under CS, implicating redundant regulators such as CAMTAs and circadian components (*CCA1*, *LHY*, *RVE*) (Wang et al., 2023a; Kim et al., 2024). Together, these layers, modulated further by dynamic DNA methylation, support membrane stability, osmotic adjustment, and antioxidant capacity, which are core determinants of CS resilience in *B. napus* (Lei et al., 2019; Niu et al., 2021; Zheng et al., 2022).

#### 3.3.2 Heat stress

In contrast to CS, HS tolerance in *B. napus* is primarily regulated through the HSF-HSP network, in which master regulators such as HSFA1 and HSFA2 drive the rapid and strong induction of HSP genes required for acquired thermotolerance. Major chaperone classes, including HSP70, HSP90, and small HSPs (e.g., HSP18.2/HSP20), preserve cellular proteostasis by preventing heat-induced protein misfolding and aggregation and promoting refolding (Verghese et al., 2012; Jacob et al., 2017; Wang et al., 2020a). Integrated multiomics further indicate support from the ER-localized unfolded protein response (UPR) and metabolomic adjustments, including the accumulation of saccharides and flavonoids, which contribute to ROS scavenging and membrane/osmotic protection (Lohani et al., 2022; Kourani et al., 2025). Transcriptomic response to HS are characterized by massive induction of HSP genes, which is essential for acquired thermotolerance (Bita and Gerats, 2013; Ohama et al., 2016; Ling et al., 2021; Boopathy et al., 2022). Notably, many HS-responsive transcripts, especially HSPs, undergo extensive alternative splicing (AS), which fine-tunes heat-responsive isoforms and may establish a priming-associated “splicing memory”, enhancing resilience during recurrent heat waves (Liu et al., 2013; Staiger and Brown, 2013; Ling et al., 2021; Wen et al., 2023).

Beyond the HSF-HSP module, the AP2/ERF TF *DREB2A* functions as is dual regulator of HS and WS responses, with its activity primarily controlled by post-translational modifications (Sakuma et al., 2006b; Morimoto et al., 2013; Mizoi et al., 2019; Han et al., 2022). HS prevents inhibitory phosphorylation within its Negative Regulatory Domain (NRD), stabilizing the protein and enabling rapid activation of thermotolerance-associated genes without requiring *de novo* transcription (Mizoi et al., 2019; Ding et al., 2020b; Kim et al., 2024). The HSF-HSP module remain central to this response. In *B. napus*, polyploidy has expanded this family to be the largest known in plants, with master regulators like *BnHSFA1a* and *HSF2* triggering a massive induction of HSP70, HSP90, and sHSPs (HSP20) (Kourani et al., 2022; Lohani et al., 2022; Quan et al., 2023). Integrated proteomics and peptidomics identify these chaperones, along with co-chaperones like *HOP3*, as immediate “first responders” in sensitive floral tissues to maintain proteostasis (Lohani et al., 2022; Rani et al., 2024; Hu et al., 2025; Markie et al., 2025). Simultaneously, metabolomic shifts result in the accumulation of saccharides and flavonoids, which provide essential osmotic protection and ROS scavenging to mitigate oxidative damage (Koscielny et al., 2018; Yu et al., 2023; Kourani et al., 2025).

Collectively, CS and HS responses in *B. napus* are governed by partially overlapping but stress-specific regulatory networks. The CBF-COR regulon predominates under CS (osmoprotetcion, membrane stabilization, antioxidant defense) (Kim et al., 2024; Qian et al., 2024), whereas the HSF-HSP-DREB2A module is central to HS tolerance (Sakuma et al., 2006b). Post-transcriptional mechanisms, including extensive AS, further refine these responses by establishing a “splicing memory” that enhances resilience during recurrent heat waves (Ling et al., 2021). The integration of QTL mapping, GWAS, and multiomics analyses consistently highlights these regulatory hubs as key genetic determinants of resilience, offering high-confidence targets for breeding climate-resilient rapeseed (Raza et al., 2021; Kim et al., 2024; Markie et al., 2025; Zheng et al., 2025).

### 3.4 Flooding/waterlogging stress

Integrated multiomics and QTL/GWAS identify candidate genes like *VHb*, *LEA4*, and *PGIP2* driving the WS tolerance in *B. napus* (Zhang et al., 2015; Hong et al., 2023; Li et al., 2024). A prominent example of this multi-omic approach is the use of parental resequencing and root transcriptomics to narrow 56 preliminary QTL candidates down to 12 high-confidence genes associated with early vigor and biomass retention. These prioritized genes are primarily involved in oxidation-reduction processes (e.g., BnCAT3.A07) and RNA/protein degradation (e.g., BnXRN3), highlighting the metabolic shift required to manage oxidative bursts and recycle damaged cellular components under hypoxia (Ding et al., 2020a). This response relies on two tightly coordinated strategies, including (i) rapid metabolic reprogramming, including anaerobic fermentation and antioxidant pathways, to sustain energy production under hypoxia conditions (Bailey-Serres and Colmer, 2014; Hong et al., 2023; Mudasir and Shahzad, 2025), and (ii) hormone-regulated morphological responses, quiescence or elongation-driven escape, fine-tuned by miRNA-mediated networks, depending on flooding severity and duration (Bailey-Serres and Colmer, 2014; Bandehagh et al., 2025; Song et al., 2025).

#### 3.4.1 Hypoxia sensing and metabolic reprogramming

Flooding-induced oxygen depletion severely restricts mitochondrial respiration, necessitating a rapid shift toward anaerobic energy metabolism. A central component is the induction of *PDC1* and *ADH1*, key enzymes in ethanolic fermentation enabling ATP production (Li et al., 2021a; Jardine and Mcdowell, 2023; Van Veen et al., 2025). In *Brassicaceae* model plant *Arabidopsis*, this response is governed by ERF-VII TFs, which function as primary oxygen sensors. Under normal conditions, these proteins are targeted for proteasomal degradation via the oxygen-dependent N-end rule pathway. However, hypoxia inhibits this pathway, leading to ERF-VII stabilization and nuclear accumulation (Schippers et al., 2024; Dalle Carbonare et al., 2025). Once stabilized, key factors such as *RAP2.12* and *ERF73*/*HRE1* directly activate a conserved suite of hypoxia-responsive genes, including *PDC1* and *ADH1* to sustain energy production through anaerobic fermentation (Licausi et al., 2010; Hess et al., 2011). Emerging multi-omics evidence from *B. napus* indicates induction of canonical hypoxia modules. iTRAQ-based proteomics identifies rapid shifts in proteins for DNA-dependent transcription and ethylene signaling (Xu et al., 2018). Metabolomic profiling highlights flavonoid biosynthesis (naringenin, epiafzelechin) and Vitamin B6 metabolism as key antioxidant pathways that manage ROS and maintain redox homeostasis (Hong et al., 2023). Furthermore, small-RNA sequencing reveals bna-miR172 and bna-miR169 families targeting ethylene-responsive factors to fine-tune growth (Song et al., 2025). QTL-linked genes like *VHb* further improve survival by enhancing oxygen uptake and anaerobic metabolic capacity (Liu et al., 2025). These expression patterns mirror the archetypal *Arabidopsis* response, supporting the conservation of the ERF-VII-centered regulatory framework in rapeseed and its evolutionary conservation across angiosperms (Zou et al., 2013; Guo et al., 2020; Ambros et al., 2022).

Apart from transcriptional control, hypoxia responses are tightly coupled to cellular energy and Ca^2+^ signaling. In rice, *CIPK15* links oxygen deficiency to sugar signaling by activating SnRK1, promoting carbohydrate mobilization to fuel anaerobic metabolism during flooding (Lee et al., 2009; Lin et al., 2014; Wurzinger et al., 2018). A proteomics study reveals a similar logic in *B. napus*, where protein phosphorylation and DNA-dependent transcription modules are rapidly rearranged to combat energy deficits (Xu et al., 2018; Ding et al., 2022). This provides a mechanistic explanation for why QTL and GWAS analyses frequently prioritize Ca^2+^/kinase signaling (e.g., CDPKs) and energy-ROS homeostasis genes like *VHb*, *LEA4*, or *P5CS1*, even when individual loci differ across populations, environments, and phenotyping platforms (Zhang et al., 2015; Ding et al., 2020a; Li et al., 2022). Furthermore, metabolomic profiling identified flavonoid biosynthesis (naringenin, epiafzelechin) and vitamin B6 metabolism (pyridoxal phosphate) as critical antioxidant buffers that maintain redox balance and cellular stability (Hong et al., 2023; Zhang et al., 2025a). Together, these findings highlight the integration of conserved hypoxia-sensing pathways with quantitative genetic variation underlying WS tolerance in *Brassica* (Bandehagh et al., 2025; Zhang et al., 2025a).

#### 3.4.2 Hormonal control of quiescence versus escape strategies

ERF-VII TFs integrate ethylene, ABA, and gibberellin (GA) signaling to regulate morphological responses during prolonged submergence (Van Veen et al., 2014; Giuntoli and Perata, 2018; Yu et al., 2019; Hartman et al., 2021; Loreti and Perata, 2023). In rice, the ERF-VII gene *Submergence1A* (*Sub1A*) exemplifies the LOQS (Lin et al., 2023a; Loreti and Perata, 2023). Transcriptomic studies show *Sub1A* is induced by ethylene to restrict shoot elongation by suppressing GA responsiveness and down-regulating *α*-amylases, thereby conserving carbohydrate reserves for post-submergence recovery (Jung et al., 2010; Fukao et al., 2012; Schmitz et al., 2013; Alpuerto et al., 2022). In contrast, deepwater rice adopts a LOES (escape strategy), in which ethylene accumulation under submergence condition activates the upstream EIN3, which transcriptionally induced the ERF-VII TFs *SNORKEL1* (*SK1*) and *SNORKEL2* (*SK2*) (Fukao et al., 2006; Hattori et al., 2009; Kazan, 2015; Chen et al., 2021a; Deng et al., 2022; Li et al., 2025). These factors promote rapid shoot elongation by enhancing GA biosynthesis and responsiveness, in part through the upregulation of GA20-oxidase genes like *SD1* (Qin et al., 2013; Leydon et al., 2025). Elevated levels subsequently activate cell-cycle regulators, including CYCB1;5, stimulating cell division and elongation to re-establish contact with atmosphere (Lopez-Juez et al., 2008; Achard et al., 2009; Lee et al., 2012; Camut et al., 2021). This coordinated hormonal and cell-cycle regulation enables rapid internodal elongation, allowing deepwater rice to re-establish contact with the atmosphere under prolonged flooding (Wang and Komatsu, 2022; Combs-Giroir and Gschwend, 2024).

In *B. napus*, multi-omics integrative analysis identifies similar hormonal crosstalk, where small-RNA sequencing reveals miRNA families (such as *bna-miR172* and *bna-miR169*) targeting ethylene-responsive factors to modulate growth (Song et al., 2025). Proteomics analyses further detects a rapid rearrangement of proteins involved in DNA-dependent transcription and protein phosphorylation to fine-tune these adaptive strategies (Xu et al., 2018). Furthermore, metabolomic profiling highlights flavonoid biosynthesis and cell wall reinforcement, specifically through polygalacturonase-inhibiting protein 2 (PGIP2), as critical components maintaining structural integrity during these developmental shifts (Hong et al., 2023; Li et al., 2024).

**Figure 2:**
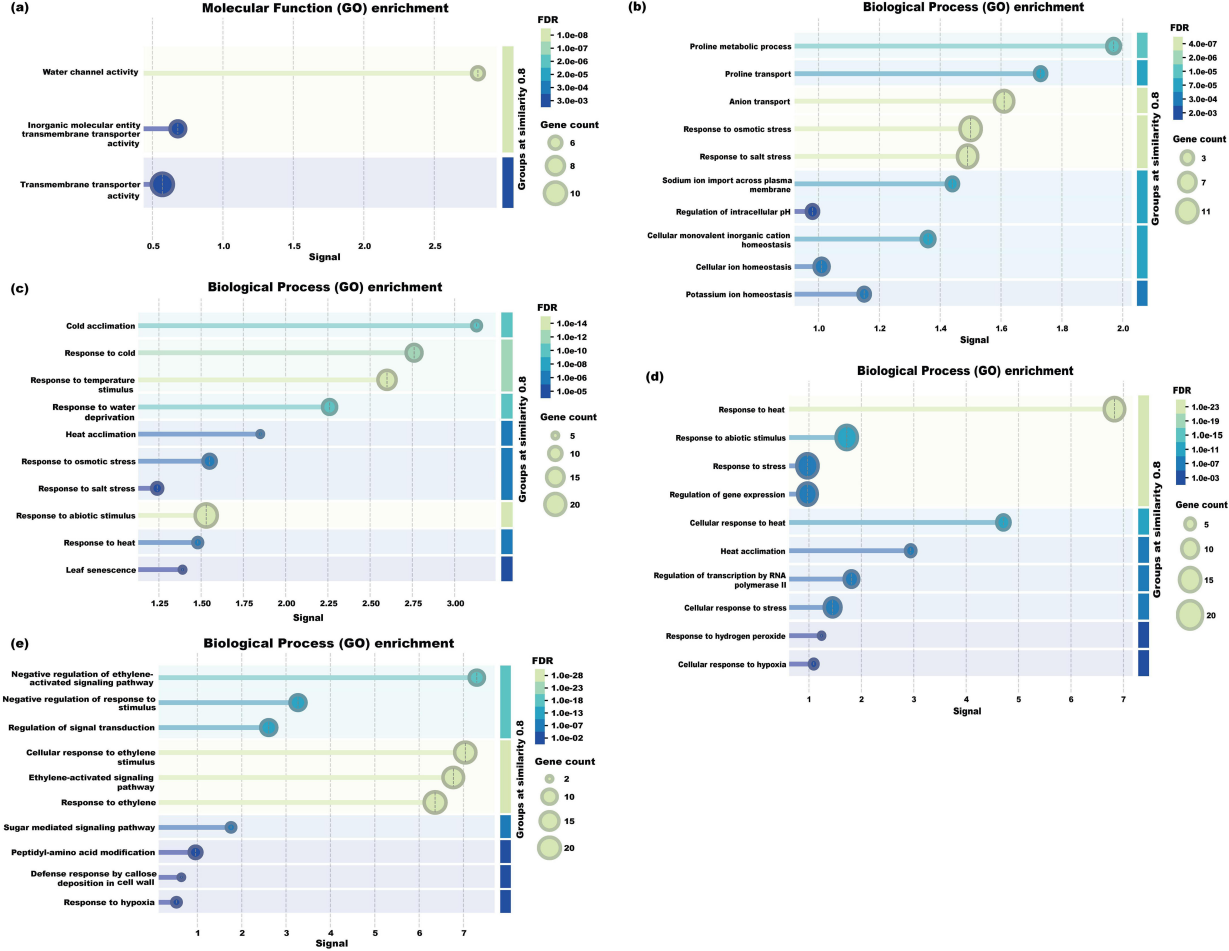
Gene ontology (GO) enrichment of QTL- and GWAS-prioritized genes regulating climate-related stress responses. **(a)** Represents GO categories related to the molecular function of genes conferring drought tolerance. The prominent terms are related to water channel activity and transmembrane transporter activity. **(b)** Important GO categories related to salinity-responsive genes include proline metabolism and transport, ion transport across plasma membrane, and cellular ion homeostasis. **(c)** In response to freezing stress, the prominent GO terms include cold acclimation and cold response, response to temperature stimulus, response to water deprivation and ABA, and response to lipid. **(d)** Under HS, GO terms include response to heat/stress, gene expression regulation, cellular response to heat, heat acclimation, regulation of transcription by polymerase II, and response to H_2_O_2_. **(e)** Waterlogging induces GO terms related to the cellular response to ethylene signaling, response to stimuli, signal transduction, sugar-mediated signaling pathway, callose deposition and hypoxia response. GO categories for molecular function (DS, as biological process data was unavailable) and biological process (salinity, cold, heat and waterlogging) were downloaded from STRING database (https://string-db.org/cgi/network) using QTL- and GWAS-prioritized genes in **Table 1** (expanded to approximately 100 edges and a PPI enrichment p-value of <1.0e-16).

**Figure 3:**
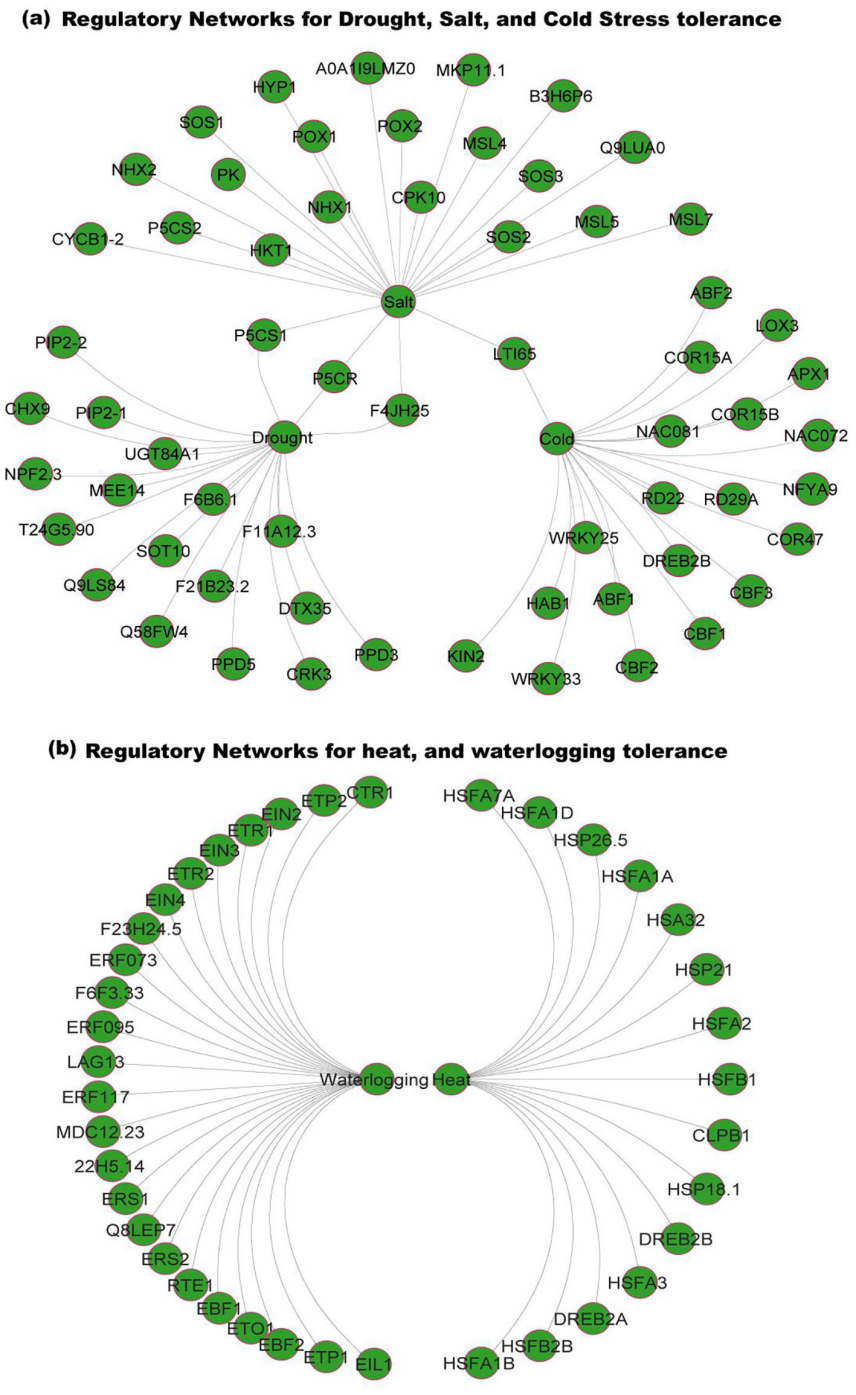
Gene regulatory network involved in climate-related stress tolerance mechanisms in *B. napus*. **(a)** Illustrates the regulatory networks of genes involved in drought, salt, and cold tolerance. Common regulatory pathways, such as proline biosynthesis, ethylene signaling, and TFs, are presented for their roles in these stresses. Genes like *P5CSA*/*P5CS1* (delta1-pyrroline-5-carboxylate) and *P5CR* (pyrroline-5-carboxylate reductase), and *F20O9.10* play central roles in osmotic adjustment under drought and salt conditions by regulating proline accumulation. The shared regulatory network *P5CS1*, *P5CR*, and *F20O9.10* is essential for maintaining cellular integrity. Ethylene-responsive genes, such as *EIN2*, *EIN3*, *ERS1*, *ERS2*, *ETR1*, *ETR2* and *RTE1*, regulate plant responses to all three stresses (drought, salt, and cold). The TFs from the AP2/ERF family, such as *DREB2A* (drought and heat), *DREB1A/C* (*CBF3*/*CBF1*, CS), and *WRKY33* and *WRKY25*, are implicated in regulating the transcription of genes involved in multiple stresses. The shared roles of *LTI65*/*RD29B* for cold and DS are also noted. Other genes like *MSL4/5/7* (mechanosensitive channels) and *NHX1* (sodium/proton antiporters) play roles in ion homeostasis and membrane stability during SS and CS. The SOS (Salt Overly Sensitive) genes, such as *SOS1* (*NHX7*, Na^+^/H^+^ antiporter), SOS2 (*CIPK24*, protein kinase) and *SOS3* (*CBL4*, Ca^2+^ binding protein), are central to maintaining cellular ionic balance under SS. *SOS1* helps extrude excess Na^+^ from the cell, while *SOS2* and *SOS3* form a signaling complex to activate SOS1, contributing to salt tolerance by controlling ion transport. *P5CS2* (*P5CSB*)/*F6B6.1* (auxin canalization protein) contributes to drought and SS responses by regulating cellular processes such as ion transport, auxin signaling, and root architecture. **(b)** Key genes involved in HS tolerance mechanisms include *HSFA1A*, *HSFA1B*, *HSFA2*, *HSFA3*, *HSFB1*, and *HSFB2B*, which regulate the expression of heat shock proteins (HSPs) and facilitate thermotolerance. These HS TFs (HSFs) interact with HSPs like *HSP18.1*, *HSP21*, and *HSP26.5* to mediate HS response by maintaining protein stability and prevent aggregation during HS. The interplay between *DREB2A* and *HSFA3* in regulating thermotolerance is also highlighted. For waterlogging tolerance, ethylene-responsive genes such as *CTR1*, *EIN3*, *EIN2*, *ETR1*, and *EFB1/EFB2* are involved in regulating plant responses to waterlogging. Ethylene acts as a key signal for the plant’s adaptation to hypoxia and waterlogged conditions. The gene network involving *ERS1* and *ERF73* plays a critical role in stress-induced root growth and membrane stability under WS. *ERF* TFs, including *ERF95* and *ERF117*, also share regulatory roles in both heat and WS responses. *RTE1*, a negative regulator of ethylene signaling, is involved in fine-tuning the ethylene response during both heat and WS. Similarly, *F23H24.5* (AP2/ERF) and *F6F3.33* (CBL-interacting serine/threonine kinase) are crucial for membrane integrity and stress signaling during these two stress conditions. For cytoscaping the networks, significant nodes were downloaded from STRING database (https://string-db.org/cgi/network) using QTL- and GWAS-prioritized genes in **Table 1** (expanded to approximately 100 edges and a PPI enrichment p-value of <1.0e-16).

Across stresses, the integrated datasets support a small number of recurrent biological modules rather than independent gene lists. Drought and salinity share osmotic adjustment, aquaporin-mediated water transport, ABA/ROS signaling, and proline metabolism, but salinity additionally requires ion-homeostasis modules involving ROS/NHX/HKT-type components (Wan et al., 2017; Wang et al., 2019a; Khanzada et al., 2020; Wassan et al., 2021; Zhang et al., 2024). Cold and heat share transcriptional stress regulation and redox protection. Specifically, cold tolerance is more robustly linked with CBF/COR-mediated acclimation, membrane remodeling, and vernalization-associated development. Whereas, heat tolerance is dominated by reproductive protection, HSF-HSP proteostasis, and carbon-allocation stability (Rahaman et al., 2017; 2018; Liang et al., 2019; Horvath et al., 2020; Chao et al., 2021; Zheng et al., 2025). Waterlogging is distinct because hypoxia imposes energy limitation, making ethylene signaling, fermentation, CIPK/SnRK1-like energy regulation, and recovery-associated redox/cell-wall repair central to tolerance (Ding et al., 2020a; Li et al., 2022; Hong et al., 2023; Li et al., 2024, 2025). These cross-stress modules provides the basis for prioritizing candidates for MAS, genomic selection, allele pyramiding, and genome editing. For functional classification and gene networks involved in the regulation of the five climate-related stresses, see **Figure 2** (GO categories) and **Figure 3** (cytoscape networks of top-selected nodes).

## 4 Functional validation and genetic engineering strategies for climate-resilient *B. napus*

To establish a breeding program aimed at enhancing the capacity of *B. napus* to withstand against multiple climate-related stresses, including drought, salt, cold, heat, and water-logging, overexpression of climate-stress responsive genes is a promising strategy. Through genetic engineering, transgenic plants with overexpressed genes can be incorporated into molecular breeding programs to ultimately develop climate-resilient *B. napus* varieties (Zhang et al., 2001; Kumar and Srivastava, 2016; Kumar et al., 2018).

### 4.1 Drought and salt tolerance mechanism

**Figure 4:**
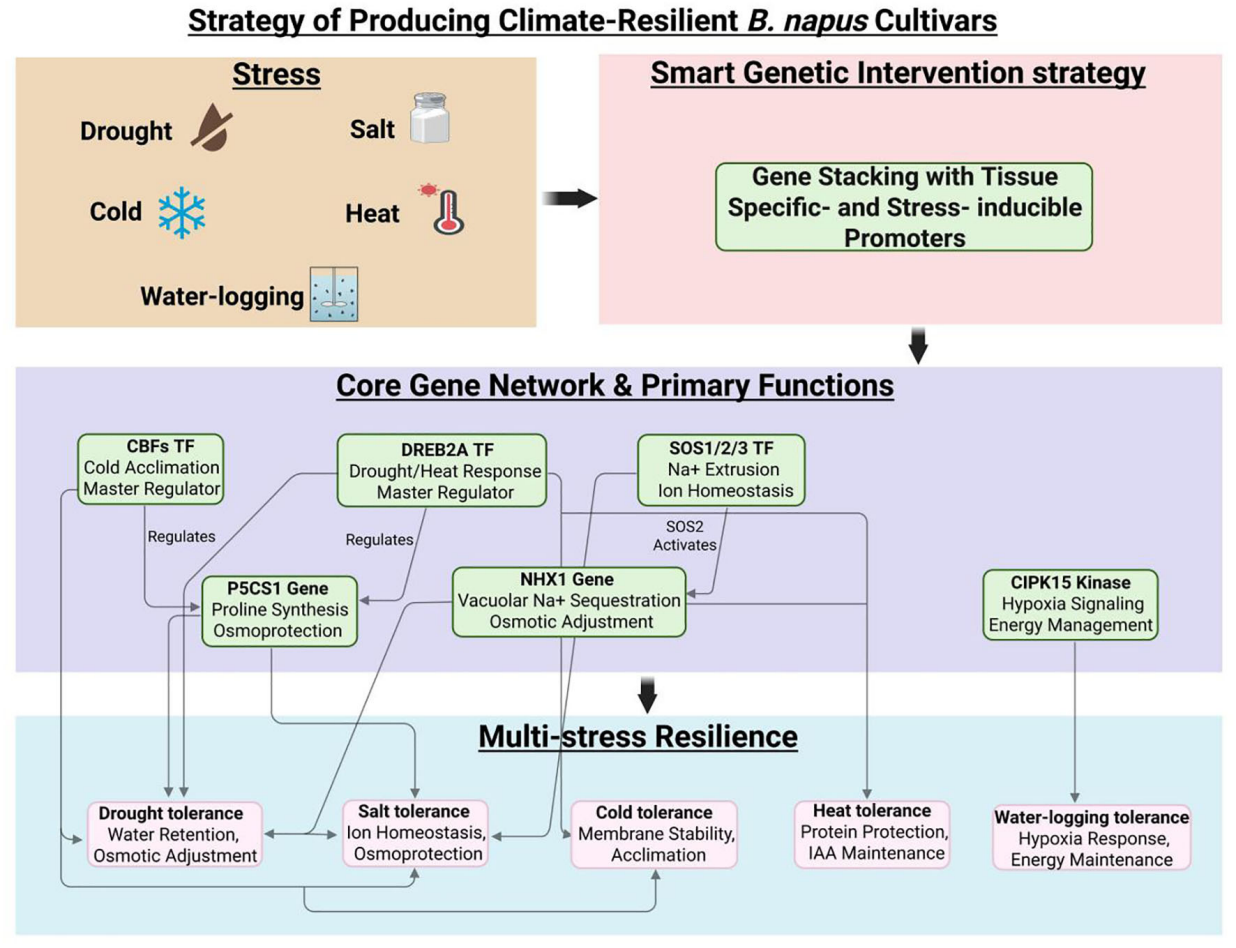
Conceptual genetic intervention strategy for enhancing multi-stress resilience in *B. napus.* This image summarizes candidate intervention modules in which selected TFs, ion transporters, osmoprotectant genes, and energy-signaling kinases may be deployed through MAS, allele pyramiding, promoter engineering, or genome editing. *CBFs* (*C-repeat Binding Factors*) and *DREB2A* (*Dehydration Responsive Element Binding Protein 2A*) are proposed to promote *P5CS1* (Δ1-Pyrroline-5-Carboxylate Synthase 1) gene expression, increasing proline accumulation and supporting osmotic adjustment and cellular protection under dehydration-related stresses (e.g., drought, cold and heat). For salinity tolerance, SOS2 is shown as a key component of NHX1 activity. Under waterlogging-induced hypoxia, the kinase CIPK15 is proposed to contribute to stress survival by supporting energy and signaling adjustments under oxygen limitation. Ultimately, these intervention modules operate through highly interconnected osmotic, redox, and energy-signaling networks, rather than functioning as isolated linear cascades.

**Figure 5:**
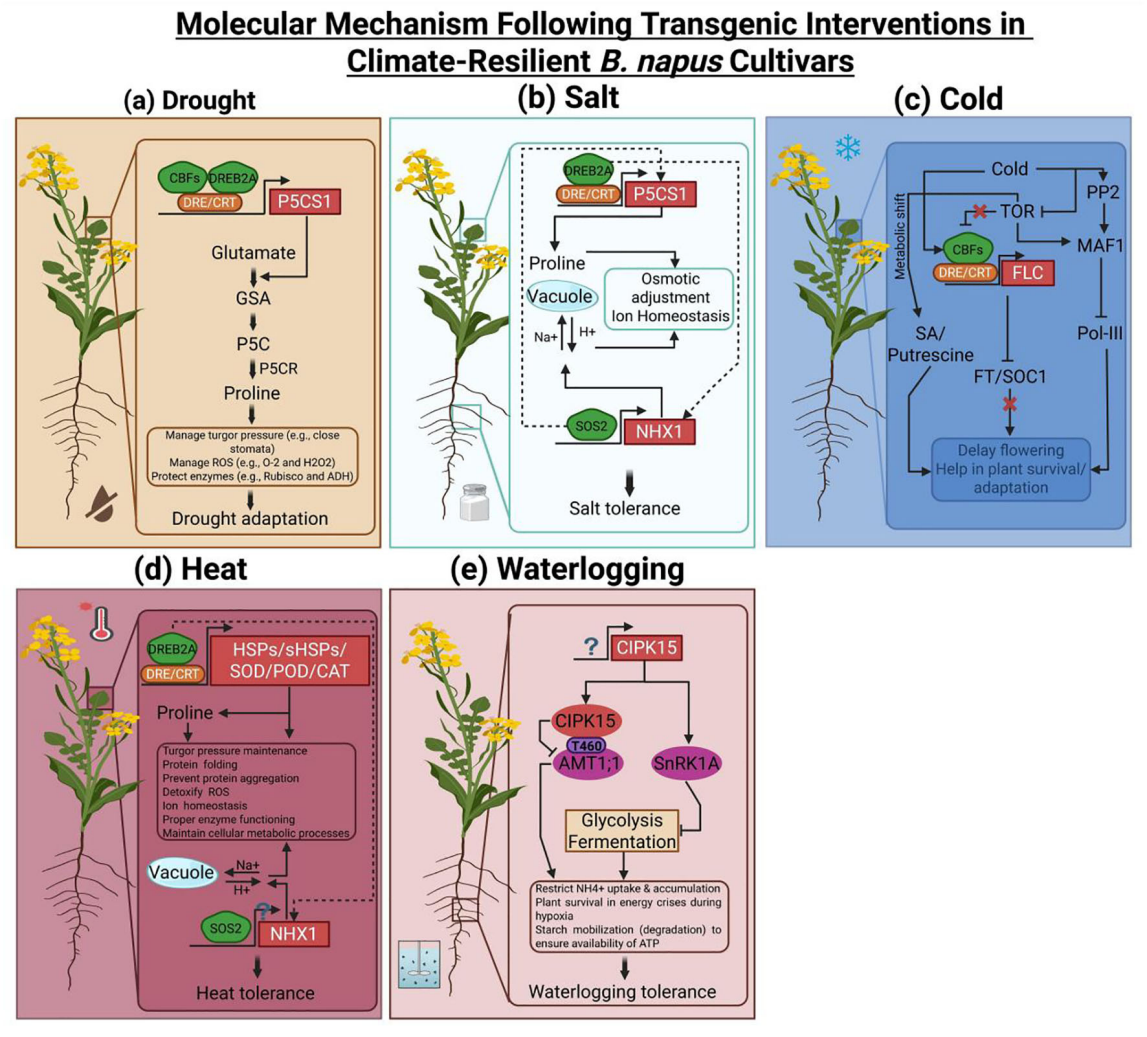
Proposed molecular mechanisms underlying transgenic interventions for climate-resilience in *B. napus*. **(a)** Under DS, TFs *CBFs* (C-repeat Binding Factors) and *DREB2A* (Dehydration Responsive Element Binding Protein 2A) are proposed to activate *P5CS1* (Δ1-pyrroline-5-carboxylate synthase 1) gene via binding to DRE/CRT promoter elements, promoting proline biosynthesis. Accumulated proline contributes to osmotic adjustment, ROS scavenging and cellular protection under dehydration. **(b)** During SS, *DREB2A*-mediated induction of *P5CS1*, together with SOS signaling pathway and vacuolar NHX1 activity, is proposed to support osmotic balance and Na^+^ sequestration, thereby enhancing ionic homeostasis. **(c)** Under CS, *CBF*-dependent signaling is suggested to coordinate growth restraint and cold acclimation through modulation of developmental and metabolic pathways, contributing to freezing tolerance. **(d)** In response to HS, *DREB2A* and HSF-HSP network induce the expression of molecular chaperones and antioxidant enzymes, supporting protein stability and redox homeostasis under elevated temperatures. **(e)** Under WS-induced hypoxia, Ca^2+^-dependent signaling pathways, including *CIPK15* (Calcineurin B-like (CBL)-interacting protein kinase 15), are proposed, based largely on the studies in rice and *Arabidopsis*, to regulate energy homeostasis through interactions with AMT1;1 (Ammonium Transporter 1;1) and SnRK1A (Snf1-related protein kinase 1A), promoting metabolic adjustment and ATP maintenance under oxygen-limited conditions.

Although DS and SS share overlapping physiological and molecular responses, DS tolerance primarily relies on osmotic adjustment and water transport efficiency, whereas SS tolerance additionally requires strict regulation of ionic homeostasis and Na^+^ detoxification (Munns, 2002; Ashraf and Mcneilly, 2004; Batool et al., 2023). In *B. napus*, both stresses are complex, multigenic traits, and QTL mapping, GWAS, multiomics profiling, and transgenic validation have collectively prioritized several candidate genes suitable for overexpression-based improvement strategies (Wan et al., 2017; Wang et al., 2017; Zhang et al., 2024; Salami et al., 2024a) (**Figures 4**, **5a, b**).

#### 4.1.1 Drought stress: osmotic adjustment and water transport

Among the most consistently prioritized DS responsive genes are those involved in osmoprotectant biosynthesis and water transport, particularly *BnaP5CS1* and *BnPIP2* family members (Yu et al., 2005; Chen et al., 2022a; Yang et al., 2022). Functional validation of the *BnaP5CS1* hub has demonstrated that overexpressing plants exhibit significantly increased RWC and reduced lipid peroxidation. These studies directly link the gene’s enzymatic activity to enhanced survival under acute water deficit, indicating its status as a high-confidence candidate for molecular breeding (Sharma et al., 2011; Chakraborty et al., 2012a; Amini et al., 2015; Mohan et al., 2025). Importantly, QTL and GWAS studies in *B. napus* frequently identified loci associated with DS related traits (e.g., relative water content, biomass retention, and yield stability) that colocalize with genes involved in proline metabolism, reinforcing *BnaP5CS1* as a high confidence candidate for genetic improvement (Wang Y et al., 2020b).

Concurrently, The PIP family represents another major class of DS associated targets. *BnaPIP2* and related *PIP* isoforms facilitate transmembrane water transport, thereby regulating root hydraulic conductivity, stomatal conductance, and whole-plant water status. Enhanced or stress-regulated expression of *PIPs* has been linked to reduced wilting, improved WUE, and faster recovery after dehydration (Wang et al., 2019b; Patel and Mishra, 2021; Sun et al., 2024). Notably, heterologous overexpression of *BnaPIP1* in *N. benthamiana* significantly enhanced drought tolerance by improving water retention and leaf turgor under water deficit conditions (Yu et al., 2005). Although demonstrated in a hetrologous system, this result underscores the functional conservation of PIP aquaporins and strongly support their candidature for targeted manipulation in *B. napus*.

Collectively, the convergence of QTL/GWAS signals, transcriptomic induction under DS, and functional overexpression studies position *BnaP5CS1* and *BnaPIP* genes as robust molecular targets for engineering drought-resilient *B. napus* cultivars (Banerjee and Roychoudhury, 2020; Raza et al., 2020; Haghpanah et al., 2024).

#### 4.1.2 Salt stress: ionic homeostasis and oxidative stress regulation

In contrast to DS, SS tolerance requires not only osmotic adjustment but also precise regulation of Na^+^ uptake, transport, sequestration, and detoxification. Genetic studies in *B. napus* and related species consistently prioritize genes from the SOS, HKT, and NHX families as central regulators of SS tolerance (Zhang et al., 2001). The SOS pathway functions as a core Ca^2+^-dependent signaling module that senses SS and promotes Na^+^ extrusion (Chakraborty et al., 2012b; Cui et al., 2020; Dai et al., 2024). This pathway comprises the Ca^2+^ sensor SOS3 (CBL4), the protein kinase SOS2 (CIPK24), and the plasma membrane Na^+^/H^+^ antiporter SOS1. Upon salt-induced Ca^2+^ influx, SOS3 activates SOS2, which subsequently phosphorylates and activates SOS1, enhancing Na^+^ efflux and preventing cytosolic Na^+^ toxicity (Sathee et al., 2015; Köster et al., 2019). Multiple transgenic studies have demonstrated that overexpression of SOS pathway components significantly improve salt tolerance, highlighting their functional importance in maintaining ionic homeostasis under saline conditions (Kumar et al., 2009; Yang et al., 2009).

In addition to Na+ extrusion, vacuolar sequestration of Na+ is a critical tolerance mechanism mediated NHX antiporters (Cui et al., 2020). In *B. napus*, transgenic overexpression overexpression of *AtNHX1* has resulted in enhanced salt tolerance, improved biomass accumulation, and increased yield components under salinity, demonstrating the translation relevance of NHX-mediated compartmentalization strategies (Zhang et al., 2001). Beyond ion transport, regulatory genes play an essential role in mitigating salt-induced oxidative stress. Several BnaWRKY TFs have been prioritized by transcriptional analyses and GWAS for salt-responsive traits. These WRKYs modulate salt tolerance by regulating downstream antioxidant enzymes (e.g., SOD, CAT, POD) and stress-responsive genes thereby limiting ROS accumulation and protecting cellular structures under high salinity (Dai et al., 2024). Functional studies indicate that overexpression of *BnaWRKYs* enhances ROS scavenging capacity and stabilizes cellular metabolism during SS, further supporting their potential as regulatory targets (Shu et al., 2022).

#### 4.1.3 Perspective for genetic improvement

Drought and salinity tolerance in *B. napus* are controlled by partially overlapping but mechanistically distinct modules. In drought improvement, potential candidates for prioritizing are those involved in osmotic adjustment, water transport, stomatal regulation, and phenology-related trade-offs. For salinity improvement, candidates must additionally target ionic homeostasis, vacuolar Na^+^ sequestration, and ROS detoxification (Wan et al., 2017; Wang et al., 2019a; Khanzada et al., 2020; Shahzad et al., 2021; Wassan et al., 2021; Zhang et al., 2024). Among these candidates, *AtNHX1*/NHX-types antiporters have the strongest transgenic support for salinity tolerance in *B. napus*. However *BnaP5CS1*, PIP aquaporins, SOS-pathway components, and *BnaWRKY* TFs should be treated as high-priority candidates whose utilization requires gene-copy-specific validation (Zhang et al., 2001; Wan et al., 2017; Wassan et al., 2021; Zhang et al., 2022b, 2024). MAS and haplotype selection are appropriate for stable QTL/GWAS-supported loci, while genome editing should focus on validated *Bna* gene copies or promoters with clear effects on stress tolerance and limited yield penalty.

### 4.2 Cold and heat tolerance mechanism

#### 4.2.1 Cold tolerance

Cold and freezing cold-tolerance in *B. napus* is largely controlled by the CBFs/DREBs transcription regulon, which functions as a central regulatory hubs linking cold perception to large-scale transcriptional reprogramming (Jaglo et al., 2001; Savitch et al., 2005) (**Figures 4**, **5c**). QTL mapping, GWAS, and transcriptome analyses consistently highlight cold-responsive loci enriched for CBF-regulated genes, supporting the prioritization of CBFs as high confidence candidates for genetic manipulation. In *B. napus*, constitutive expression of *Arabidopsis AtCBF1*, *AtCBF2*, and *AtCBF3* results in strong upregulation of orthologous cold-responsive genes, including *Bna115* (ortholog of *AtCOR15A*) and *Bna28* (ortholog of *AtCOR6.6*), even in the absence of cold acclimation (Jaglo et al., 2001). This regulon-engineering approach enhances freezing tolerance by approximately 2.6 °C in non-acclimated plants and by more than 4 °C following acclimation, demonstrating that activation of CBF pathway is sufficient to confer substantial cold tolerance in rapeseed (Jaglo et al., 2001). Functional validation using *B. napus* homologs further strengthens this conclusion. Overexpression of *BnaCBF5* and *BnaCBF17* confers constitutive freezing tolerance, increasing the lethal temperature (LT_50_) from -3.7 °C in wild type plants to -10.2 °C in the transgenic lines (Savitch et al., 2005). This enhanced tolerance is associated with coordinated activation of downstream protective mechanisms, including the accumulation of cryoprotective polypeptides, elevated levels of proline, and increased concentration of soluble sugars such as sucrose, raffinose, glucose, and fructose (Gilmour et al., 2000). These metabolic adjustments are driven, in part, by upregulation of enzymes involved osmoprotectant biosynthesis, including P5CS, there stabilizing membranes and mitigating freezing-induced cellular damage. Despite their strong protective effects, constitutive CBF overexpression is often associated with growth retardation and delayed flowering. To address this limitation, the use of CS inducible or stress-responsive promoters has been proposed and experimentally validated as an effective strategy to preserve agronomic performance while maintaining enhanced freezing tolerance (Jaglo et al., 2001; Abdullah et al., 2022). Together, these findings establish CBF pathway manipulation as an experimentally supported approach that require further validation for developing cold-resistant *B. napus* cultivars, consistent with QTL.GWAS evidence identifying CBF-associated loci as key determinants of winter hardiness.

#### 4.2.2 Heat tolerance

In contrast to CS, HS tolerance in *B. napus* is primarily mediated by the HSF-HSP regulatory network, which maintains proteostasis under elevated temperatures (**Figures 4**, **5d**). Genome-wide analyses reveal that *B. napus* contains 64 HSF, reflecting extensive gene family expansion following allopolyploidy and underscoring the importance of transcriptional control in thermotolerance (Zhu et al., 2017; Kourani et al., 2022). QTL and GWAS studies for heat-related traits frequently co-localize with regions enriched in HSFs, HSPs and upstream regulatory TFs, reinforcing their prioritization for functional validation. Among non-HSF regulators, *DREB2A*, plays pivotal role integrating heat, dehydration, and SS (Lata and Prasad, 2011; Wang et al., 2023b). Under non-stress conditions, inhibitory phosphorylation within its negative regulatory domain (NRD) suppresses activity; HS alleviates this repression, allowing rapid accumulation of functional DREB2A protein. Overexpression of *DREB2A* or its constitutive form (*DREB2A-CA*) significantly enhances thermotolerance by inducing a broad suite of *HSP* genes and activating *HSF3A* (Lim et al., 2007; Qin et al., 2007; Lata and Prasad, 2011), thereby linking the DREB2A pathway directly to the canonical heat shock response (Feng and Xia, 2025). As downstream effectors, HSPs, including HSP70 and small HSPs, function as molecular chaperones that prevent protein denaturation folding and aggregation during HS, ensuring cellular viability (Waters and Vierling, 2020; Cantila et al., 2024; Batool et al., 2025). Transcriptomic analyses consistently show strong induction of these genes in heat-tolerant *B. napus* genotypes, supporting their role as core determinants of thermotolerance.

Additional regulatory layers are provided by protein-turnover mechanisms. Ectopic expression of *BnaTR1*, a membrane-associated E3 ubiquitin ligase, significantly enhances heat tolerance in *B. napus* by modulating the transcription of HSFs (e.g., HSF1A) and improving the protein quality control under high temperatures (Liu et al., 2014). This highlights the importance of proteostasis regulation beyond HSP induction and provides an additional, genetically tractable target for improving heat tolerance.

#### 4.2.3 Perspective for genetic improvement

Cold and heat tolerance in *B. napus* are governed by distinct but partially connected transcriptional and proteostasis networks. Cold tolerance is strongly supported by functional evidence for CBF/BnaCBF5/BnaCBF17-mediated cold acclimation, but constitutive activation can cause growth retardation and delayed flowering. So, stress-inducible or tissue-specific promoters are preferable for deployment (Jaglo et al., 2001; Savitch et al., 2005; Abdullah et al., 2022). Heat tolerance is more closely linked with HSF-HSP-DREB2A, BnaTR1, and reproductive-stage proteostasis. However, the phenotypic impact of these candidates requires rigorous evaluation through metrics such as pollen fertility, pod retention, and seed set, rather than relying exclusively on vegetative survival (Liu et al., 2014; Rahaman et al., 2017; Zhu et al., 2017; Rahaman et al., 2018; Liang et al., 2019; Wang et al., 2023b). Consequently, regulators such as CBFs, DREB2A, HSFs/HSPs, and BnaTR1 achieved validated, high priority status when mapping, expression profiling, functional assays, and yield-related phenotypes converge.

### 4.3 Flooding/waterlogging tolerance mechanism

Improving tolerance to hypoxia and partial submergence caused by waterlogging through the overexpression of WS-responsive genes represents a promising strategy for developing tolerant *B. napus* cultivars (Mickelbart et al., 2015; Voesenek and Bailey‐Serres, 2015) (**Figures 4**, **5e**). Unlike complete submergence, waterlogging primarily induces root-zone hypoxia, resulting in restricted oxygen diffusion, impaired mitochondrial respiration, and severe metabolic constraints (Fukao et al., 2019; Pan et al., 2021; Daniel and Hartman, 2024). Although hypoxia signaling mechanisms are evolutionary conserved, their regulatory hierarchies and phenotypic outputs can vary across species (Lee and Bailey‐Serres, 2021; Gibbs et al., 2025), necessitating functional validation in *B. napus*.

#### 4.3.1 ERF-VII TFs as central regulators of hypoxia

Tolerance building on the ERF-VII-centered regulatory framework previously detailed (see Section 3.4), these TFs act as upstream transcriptional regulators for hypoxia and submergence tolerance (Voesenek and Bailey‐Serres, 2015). Once stabilized under low-oxygen conditions, they directly induce genes involved in glycolysis and ethanolic fermentation, including *PDC1* and *ADH*, thereby sustaining ATP production when oxidative phsophorylation is compromised (Zhou et al., 2011; Voesenek and Bailey‐Serres, 2015; Loreti and Striker, 2020; Combs-Giroir and Gschwend, 2024). Emerging transcriptomic evidence from *B. napus* indicates a strong induction of fermentation-related genes and ERF-associated regulatory modules under waterlogging, supporting the conservation of this regulatory mechanism in rapeseed (Guo et al., 2020; Ambros et al., 2022).

#### 4.3.2 Proof-of-concept from heterologous ERF-VII overexpression

The functional relevance of ERF-VII regulators has been demonstrated through heterologous expression studies, providing proof-of-concept for their conserved roles across plant species. Notably, overexpression of the rice ERF-VII gene *OsSUB1A* in tobacco significantly enhanced tolerance to hypoxic conditions, as evidenced by increased ADH and PDC activities and improved survival (Fukao and Bailey-Serres, 2008). In addition to metabolic reprogramming, *OsSUB1A*-overexpressing plants showed marked activation of antioxidant enzymes, including SOD, APX, and CAT. This coordinated regulation is critical because hypoxia and subsequent reoxygenation generate substantial oxidative stress (Fukao and Bailey-Serres, 2008; Zhou et al., 2011). While these studies highlight the functional conservation of ERF-VII regulators, they serve as a proof-of-concept rather than direct translational evidence for *B. napus*, necessitating further species-specific validation.

#### 4.3.3 Hormonal regulation and growth restraint under waterlogging

In addition to metabolic and redox regulation, ERF-VII-dependent hypoxia responses modulate hormonal signaling, particularly GA. Reduced GA-mediated elongation under low-oxygen conditions conserves energy reserves and enhances survival during prolonged waterlogging, reflecting a quiscence-based strategy rather than escape growth (Schmitz et al., 2013; Voesenek and Bailey‐Serres, 2015). This growth restraint is particularly relevant for *B. napus*, which lacks the extreme elongation capacity observed un flood-adapted species such as deepwater rice.

#### 4.3.4 CIPK15 signaling and integration with QTL/GWAS evidence

Beyond ERF-VII-mediated transcriptional control, Ca^2+^-dependent signaling pathways play a complementary role in hypoxia tolerance. In rice, CIPK15 links hypoxia-induced Ca^2+^ signaling with energy homeostasis by activating SnRK1, thereby promoting carbohydrate mobilization to fuel anaerobic metabolism (Lee et al., 2009; Fagerstedt et al., 2024). In *B. napus*, GWAS and expression analyses have identified *CIPK15* homologs as candidate genes associated with WS tolerance, particularly for traits related to seedling survival and post-stress recovery (Li et al., 2022). Although functional validation in rapeseed is still limited, the convergence of mapping signals and conserved molecular function robustly supports *CIPK15* as a promising target for further characterization and potential overexpression-based improvement.

#### 4.3.5 Perspective for genetic improvement

Waterlogging tolerance cannot be reduced to a single-gene over-expression paradigm. The strongest current evidence supports a pathway-level model involving ERF-VII/EIN3/ERF73-mediated hypoxia signaling, PDC/ADH-linked fermentation, antioxidant protection, growth restraint, and CIPK15/SnRK1-like energy regulation (Lee et al., 2009; Voesenek and Bailey‐Serres, 2015; Ding et al., 2020a; Li et al., 2022, 2025). However, much of functional evidence still comes from conserved mechanisms or heterologous systems, while direct validation in *B*. *napus* remains limited (Fukao and Bailey-Serres, 2008; Voesenek and Bailey‐Serres, 2015; Li et al., 2022). Therefore, waterlogging candidates should first be tested through *B*. *napus* gene-copy-specific expression analysis, near-isogenic lines, CRISPR/Cas or promoter-editing approaches, and recovery-stage phenotyping. For breeding purposes, researchers should prioritize survival, biomass recovery, root function, seed yield, and oil quality to avoid selecting quiescence alleles that improve survival but reduce post-stress productivity (Voesenek and Bailey‐Serres, 2015; Wang et al., 2020b;Li et al., 2022).

Overall, this integrated molecular framework, encompassing transcriptional regulators, ion transporters, osmoprotectant biosynthesis, and energy-sensing kinases, provides a promising conceptual basis for improving abiotic stress resilience in *B. napus*. However, functional validation in rapeseed and assessment of trait stability across diverse environment remain critical future steps.

## 5 Conclusions and future perspectives

Integrating QTL mapping, GWAS, transcriptomic, proteomic, metabolomic, and functional evidence has clarified the genetic and molecular basis of climate-resilience in *B. napus*. Importantly, stress tolerance is not controlled by isolated genes, but by interacting modules that connect genetic loci to physiological outcomes. Drought and salinity converge on ABA signaling, proline biosynthesis, aquaporin-mediated water transport, ROS buffering, and, in salinity, SOS/NHX/HKT-linked ion homeostasis. Cold tolerance is centered on CBF/DREB-COR signaling, membrane stabilization, ROS detoxification, and vernalization-linked developmental regulation. Heat tolerance is dominated by reproductive protection, HSF-HSP-DREB2A-mediated proteostasis, source-sink regulation, and redox detoxification. Waterlogging tolerance depends on hypoxia and ethylene signaling, redox protection, cell-wall and membrane recovery, and CIPK15/SnRK1-like energy regulation.

The evidence also shows that candidate genes must be ranked by validation strength. Positional genes located within QTL/GWAS intervals are useful for hypothesis generation, but they should not be treated as causal targets without expression, pathway, or functional support. Expression-supported candidates such as stress-responsive TFs, transporters, redox enzymes, and metabolic genes are stronger, but they still require allele-level or gene-copy-specific validation in *B*. *napus*. Experimentally supported genes, including selected CBF homologs and NHX-type antiporters, provide promising applicable targets, although their effects may depend on promoter choice, genetic background, developmental stage, and stress severity.

For breeding, the most useful targets are not single genes but combinations of loci that jointly improve stress response and recovery. MAS and haplotype selection are appropriate for stable QTL/GWAS loci and recurrent genomic hotspots, whereas genomic selection may better capture polygenic small-effect variation, especially for drought, salinity, and waterlogging recovery. Genome editing, base editing, prime editing, and promoter engineering should be reserved for candidates with strong convergence among mapping evidence, stress-responsive expression, biological function, and phenotype-level validation.

Important trade-offs remain unresolved. Early flowering can help escape terminal drought but may reduce biomass or yield potential when drought timing varies. Strong quiescence can improve waterlogging survival but may reduce post-stress recovery and productivity. Constitutive CBF regulation can improve freezing tolerance but may delay flowering or restrict growth. Similarly, manipulation of ABA, ROS, aquaporin, and ion-homeostasis pathways may improve stress tolerance in one context while creating penalties in growth, seed yield, or oil quality in another. These trade-offs must be evaluated under field-relevant conditions rather than inferred from single-stress seedling assays.

Future research should prioritize four areas. First, candidate genes identified through QTL/GWAS and transcriptomic overlap should be validated using near-isogenic lines, mutants, transgenic assays, CRISPR/Cas editing, and promoter-specific perturbations in *B*. *napus*. Second, combined-stress experiments, such as drought-heat, salinity-heat, and waterlogging-heat, should be used because field stress responses often differ from single-stress responses. Third, temporal multi-omics should be integrated with UAV-based and AI-assisted phenotyping to connect molecular signatures with recovery dynamics, yield, and oil quality. Finally, validated alleles should be incorporated into breeding pipelines through MAS, genomic selection, allele pyramiding, and genome editing to develop high-yielding, climate-resilient *B*. *napus* cultivars.
